# Sex-specific systemic and brain metabolic responses to a standardized ketogenic diet in mice

**DOI:** 10.1038/s41684-026-01732-7

**Published:** 2026-05-12

**Authors:** Charlotte Lorin, Giacomo Diaceri, Miguel Garcia, Emmanuelle Logette, Henry Markram, Daniel Keller

**Affiliations:** 1 Blue Brain Project, Swiss Federal Institute of Technology Lausanne (EPFL), Campus Biotech, Geneva, Switzerland; 2 Center of PhenoGenomics, Swiss Federal Institute of Technology Lausanne (EPFL), Lausanne, Switzerland; 3 Flow Cytometry Core Facility, Swiss Federal Institute of Technology Lausanne (EPFL), Lausanne, Switzerland

**Keywords:** Fat metabolism, Cellular neuroscience, Astrocyte, Neuronal physiology

## Abstract

Ketogenic diets (KDs) are widely used in preclinical research to investigate metabolism and neurological function, yet many studies lack methodological consistency and frequently exclude female animals. Here we investigated sex-specific systemic and brain metabolic responses to a standardized KD in mice, highlighting the need to include both sexes. Using the widely used C57BL/6J mouse strain and the Bio-Serv KD, systemic and brain cell metabolism were examined in both sexes. Significant sex-based metabolic differences, probably influenced by hormones, were observed: females were leaner, exhibited higher interindividual variability in weight loss, higher baseline ketone (β-hydroxybutyrate) levels and a faster but less pronounced drop in glycemia compared with males. By contrast, cerebral metabolism appeared stable across sexes, with no significant differences detected in isolated brain cells, suggesting that sex-specific systemic adaptations are counterbalanced at the brain level to maintain functional stability. Regarding brain cell analysis, a lactate gradient from astrocytes to neurons was observed, reflecting preferential astrocytic lactate production and neuronal utilization, along with distinct glucose and glutamine distributions. Overall, our findings validate an animal model of sustained, stable ketosis and emphasize the importance of including both sexes in KD research, providing a foundation for studying sex-specific metabolic adaptations and informing potential personalized dietary strategies.

## Abstract (web summary)

This study examines sex-specific metabolic responses to a standardized ketogenic diet in mice, finding marked systemic differences but stable brain metabolism across sexes, underscoring the need to include both males and females in studies.

## Main

The ketogenic diet (KD) was introduced in the early twentieth century to treat drug-resistant epilepsy in humans^1–4^. Since 2008, its benefits have extended beyond epilepsy, impacting conditions such as obesity^5^, overweight^6^, diabetes^7^, neurodegenerative diseases^8^, cancers^9^, migraine^10^, bipolar disorder and schizophrenia^11^. These pathologies are major global economic and health concerns^12,13^, and KD could offer a complementary approach to the current treatments. KD has been associated with weight loss, improved glycemic control in obesity and diabetes^14,15^, reduced tumor growth in cancer^16–18^, improved quality of life^19,20^, extended survival^21,22^ and enhanced effectiveness of radiotherapy and chemotherapy^23–25^.

The KD for rodents consists of protein, which typically accounts for 8–12% of total calories; carbohydrates, which are restricted to 2–5%; and fat, which constitutes 70–80% of total calories^26,27^. This substantial reduction in carbohydrates in the diet depletes glycogen, induces gluconeogenesis and triggers ketogenesis, producing ketone bodies (KBs) such as β-hydroxybutyrate (β-HB), acetoacetate (AcAc) and acetone as primary energy sources^14,28^. KB production mainly occurs in the liver through β-oxidation of fatty acids (FAs) derived from dietary fat and lipolysis of triglyceride (TG), promoted by reduced insulin levels^14,29–32^. Acetone is eliminated by breathing and in urine^31,33,34^, while AcAc and β-HB enter systemic circulation via monocarboxylate transporters 1 and 2 (MCT1 and MCT2)^25,31,35^, for mitochondrial catabolism, generating adenosine triphosphate (ATP) through the tricarboxylic acid cycle^29,31^.

Sex hormones may impact KB production^36–40^. Estrogen inhibits lipogenesis and promotes FA oxidation^40,41^, while progesterone enhances TG synthesis^40,42^. Testosterone stimulates lipolysis, with efficacy fluctuating according to the body fat distribution, either subcutaneous or visceral^43–45^. Thus, estrogen and testosterone are hypothesized to increase KB production, while progesterone could reduce it. However, the precise underlying mechanisms remain largely unexplored, and no studies so far have specifically investigated how hormonal fluctuations across the menstrual cycle impact KB levels. Further research is needed to clarify these potential interactions and their physiological relevance.

**Fig. 1:**
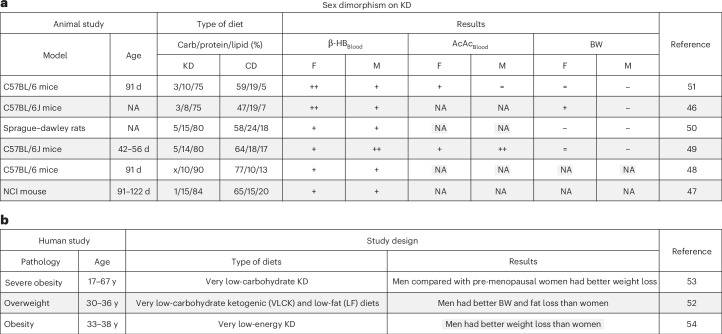
Overview of sex dimorphism research under KD. **a**, Animal studies. The table categorizes key parameters, including the animal model, age (days, d), macronutrient composition (%) in carbohydrate (Carb), protein, and lipid of both the CD and KD. The table also presents the impacts of KD on blood levels of β-HB, AcAc and BW, with changes indicated by increase (+, ++), decrease (−, −−), steady-state (=) or unknown effects (NA, not applicable) versus CD diet, according to sex, female (F) and male (M). **b**, Human studies. The table categorizes participant pathologies, age (years, y), the types of diets investigated, and the main results obtained.

Given the growing popularity of KD, understanding sex differences in KD outcomes is critical. However, only a few studies have examined these effects (Fig. 1), with most focusing exclusively on male animals across multiple models, ages and diets, leading to inconsistent data and interpretation^46–54^. In this study, we sought to standardize a KD mouse model by including both sexes to enhance data reproducibility, reliability and comparability. To this end, the impacts of food sources (Safe and Bio-Serv) were first examined as potential variability factors. Our findings revealed that food quality affects metabolism, emphasizing the importance of researchers ensuring the diet is consistent and appropriately formulated, before and during the dietary intervention. Moreover, a sex-based dimorphism was discovered in systemic KD responses but not in those of neuroglial cells, suggesting that underlying regulatory mechanisms maintain a brain molecular balance regardless of sex and diet in healthy individuals.

## Results

### Metabolic response of male mice to the Safe KD and CD

**Fig. 2:**
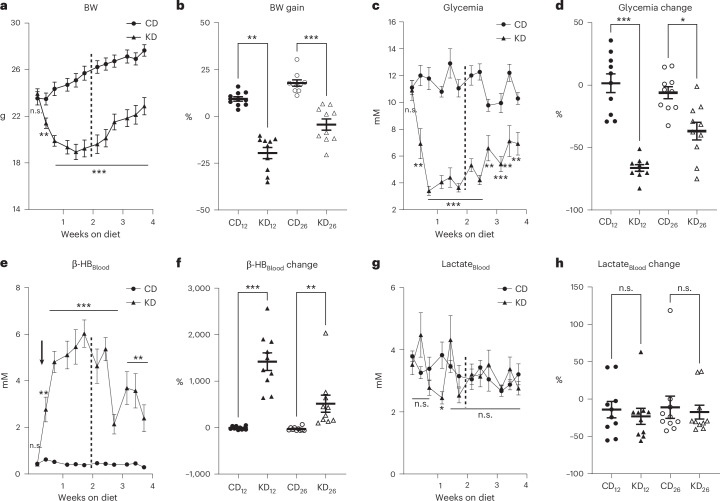
Metabolic ketosis biomarkers in male mice on Safe’s KD. **a**, BW (g); RM ANOVA yields *P* = 1.0 × 10^−5^ between KD and CD. **b**, BW gain (%) between CD-fed (black circle) and KD-fed (black triangle) mice at 12 and 26 days. **c**, Glycemia (mM), RM ANOVA yields *P* = 9.1 × 10^−9^ between KD and CD. **d**, Glycemia change (%) between CD-fed (black circle) and KD-fed (black triangle) mice at 12 and 26 days. **e**, Blood β-HB (mM) measured over 1 month in mice fed either the CD (black circle) or the KD (black triangle). RM ANOVA yields *P* = 3.2 × 10^−10^ between diets. **f**, Blood β-HB change (%) between CD-fed (black circle) and KD-fed (black triangle) mice at 12 and 26 days. **g**, Blood lactate (mM) measured as in **e**. RM ANOVA yields *P* = 0.046 between diets. **h**, Blood lactate change (%) between CD-fed (black circle) and KD-fed (black triangle) mice at 12 and 26 days. The dotted black lines mark the end of the second week of the dietary intervention. *N* = 10 male mice per diet group. Data are presented as mean ± s.e.m.; ***P < 0.001, **P < 0.01, *P < 0.05, not significant (n.s.), *P* > 0.05 (Supplementary Tables 1–8).

As a preliminary investigation for choosing foods, Safe’s diets, the usual brand used in our animal facility, were evaluated. They were tested exclusively on male mice to facilitate comparison with existing literature. After 1 month, Safe’s control diet (CD) induced an 18.0% ± 1.7% physiological increase in body weight (BW) and only a 6.1% ± 4.8% decrease in glycemia (mean ± standard error of the mea (s.e.m.)). By contrast, after 2 weeks on Safe’s KD (marked by dotted lines in Fig. 2), BW and glycemia decreased by 20.0% ± 3.0% and 66.3% ± 2.6% on day 12, respectively, and then slowly increased to a reduction of 4.4% ± 3.0% and 36.8% ± 7.1% on day 26 (Fig. 2a–d, Supplementary Figs. 1 and 2 and Supplementary Tables 1–4). Similarly, blood β-HB levels remained low and stable on CD, ranging from 0.4 ± 0.03 to 0.3 ± 0.03 mM during the 4 weeks. By contrast, blood β-HB increased 182-fold on KD, before dropping after the second week (marked by dotted lines in Fig. 2), from 6.0 ± 0.6 to 2.4 ± 0.6 mM (Fig. 2e,f, Supplementary Fig. 3 and Supplementary Tables 5 and 6). Finally, blood lactate levels, often used as a marker of cellular hypoxia and multiple organ failure^55^, were measured to reveal the metabolic adaptation to the KD^56^. They remained stable across both diets, from 3.8 ± 0.2 to 3.2 ± 0.3 mM on CD and from 3.5 ± 0.3 to 2.8 ± 0.2 mM on KD (Fig. 2g,h, Supplementary Fig. 4 and Supplementary Tables 7 and 8). Repeated-measures (RM) analysis of variance (ANOVA) revealed a statistically significant difference between the KD and CD time course for BW, glycemia and β-HB (Fig. 2a,c,e, *P* values in caption). The *P* value for the lactate effect was statistically significant (Fig. 2g, *P* values in caption).

Safe’s KD induced ketosis, as indicated by decreased BW, lower glycemia and increased β-HB levels during the first 2 weeks compared with Safe’s CD group. However, these parameters unexpectedly reversed after this period. Upon investigation, the food appeared to be stratified into layers (see ‘Diets’ in Methods for details and photos), suggesting a heterogeneous nutrient composition that probably contributed to the observed metabolic inconsistency. Consequently, Safe’s KD was not tested on female mice. To ensure consistent nutrient delivery and reproducible metabolic effects, subsequent investigations in both sexes used only Bio-Serv’s KD, which is well mixed, standardized and used in preclinical KD studies.

### BW dynamics under the Bio-Serv CD and KD

**Fig. 3:**
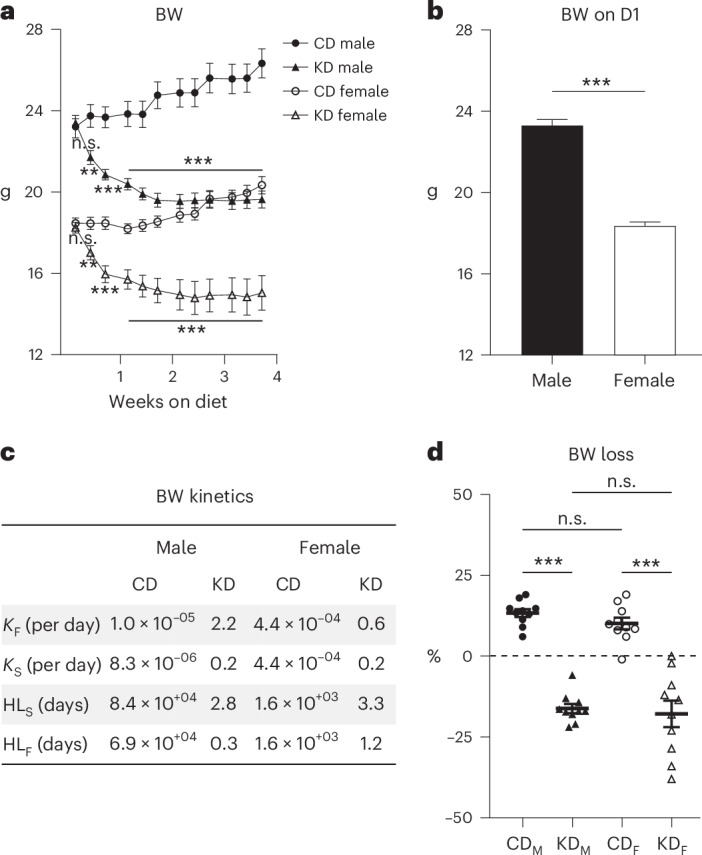
BW analysis of mice on Bio-Serv chow. **a**, BW (g) of male (black symbols) and female (white symbols) mice fed CD (circles) or KD (triangles) over 1 month. RM ANOVA yields *P* = 8.4 × 10^−6^ between CD and KD for males and *P* = 8.6 × 10^−5^ between CD and KD diets for females. **b**, Initial BW on day 1 (D1, g) compared between male (black bar) and female (white bar) mice on Safe’s CD. **c**, Kinetics parameters characterizing the BW over the 4-week dietary intervention, including fast and slow decay constants (*K*_F_, *K*_S_, per day), and half-lives fast and slow (HL_F_, HL_S_, days). **d**, BW loss (%) from the first to the last weightings in CD-fed males (black circle, CD_M_), KD-fed males (black triangle, KD_M_), CD-fed females (white circle, CD_F_) and KD-fed females (white triangle, KD_F_). *N* = 10 mice per diet group. Data are presented as mean ± s.e.m.; ***P < 0.001, **P < 0.01, not significant (n.s.), *P* > 0.05 (Supplementary Tables 9–14).

Before starting the specific regimens, all mice were acclimated and fed the usual Safe’s CD from the animal facility (Fig. 3a,b, Supplementary Figs. 5 and 6 and Supplementary Tables 9–12). Following this period, mice were switched to either Bio-Serv’s CD or KD. Both sexes displayed steady weight gain on Bio-Serv’s CD during the 4 weeks. For males, *K*_fast_ and *K*_slow_ rate constants were 1 × 10^−5^ and 8.3 × 10^−6^ per day, respectively, with a half-life_fast_ (HL_F_) and half-life_slow_ (HL_S_) of 6.9 × 10^4^ and 8.4 × 10^4^ days, respectively. For females, *K*_fast_ and *K*_slow_ were 4.4 × 10^−4^ per day, and HL_F_ and HL_S_ were similar at 1.6 × 10^3^ days (Fig. 3c, Supplementary Fig. 7a and Supplementary Table 13). Conversely, on KD, a rapid initial weight loss with *K*_fast_ of 2.2 and 0.6 per day and HL_F_ of 0.3 and 1.2 days, for males and females, respectively, was followed by a slower loss with *K*_slow_ of 0.2 per day for both sexes and HL_S_ of 2.8 and 3.3 days, for males and females, respectively (Fig. 3c, Supplementary Fig. 7a and Supplementary Table 13). Ultimately, both sexes showed similar percent weight reductions on KD compared with the Bio-Serv’s CD group (Tukey test, *P* = 0.96) of 16.2% ± 1.4% and 17.8% ± 4.1%, respectively (Fig. 3d), although females showed greater data variability with a standard deviation (s.d.) of 12.9% compared with 4.5% for males (Fig. 3d and Supplementary Table 14). RM ANOVA of the time course showed statistically significant differences between the CD and KD diets for both males and females (Fig. 3a, *P* values in caption).

### Impacts of Bio-Serv diets on blood glucose regulation

**Fig. 4:**
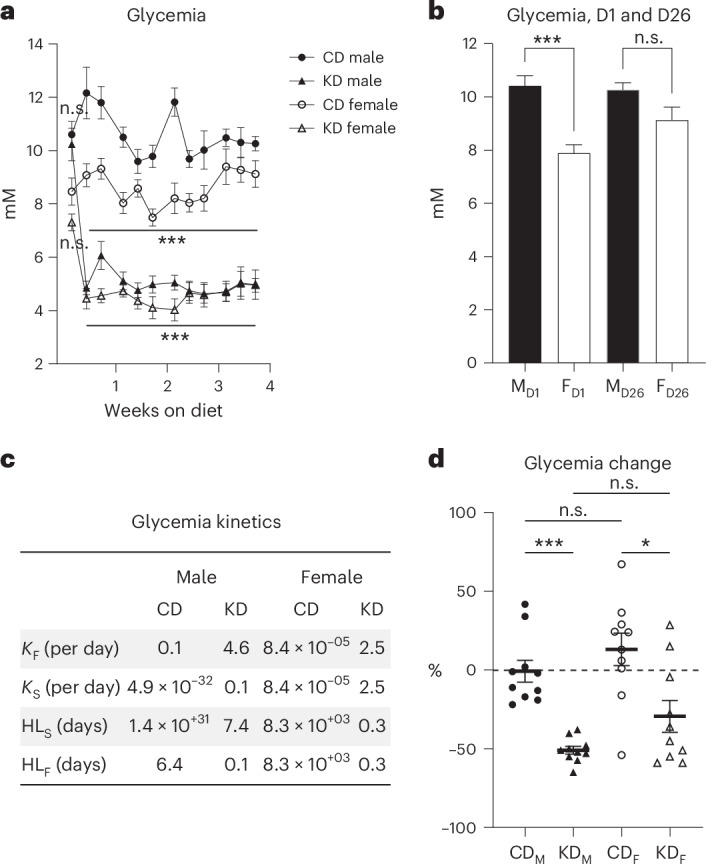
Glycemia analysis of mice on Bio-Serv chow. **a**, Glycemia (mM) in male (black symbols) and female (white symbols) mice fed CD (circles) or KD (triangles) over 1 month. RM ANOVA yields *P* = 3.2 × 10^−12^ between CD and KD diets for males and *P* = 1.8 × 10^−5^ between diets for females. **b**, Comparison of glycemia (mM) on days 1 (D1) and 26 (D26) between male (M, black bar) and female (F, white bar) mice. Day 1 data represent mice fed Safe’s CD, while day 26 data represent mice fed Bio-Serv CD. **c**, Kinetics parameters characterizing glycemia changes over the 4-week dietary intervention, including fast and slow decay constants (*K*_F_, *K*_S_, per day), and half-lives fast and slow (HL_F_, HL_S_, days). **d**, Glycemia change (%) from the first to the last measurement in CD-fed males (black circle, CD_M_), KD-fed males (black triangle, KD_M_), CD-fed females (white circle, CD_F_) and KD-fed females (white triangle, KD_F_). *N* = 10 mice per diet group. Data are presented as mean ± s.e.m.; ***P < 0.001, *P < 0.05, not significant (n.s.), *P* > 0.05 (Supplementary Tables 15–19).

Given the reduced carbohydrate intake on Bio-Serv KD (2%) versus CD (69%), glycemia was a key parameter to monitor ketosis (Fig. 4a, Supplementary Figs. 8 and 9 and Supplementary Tables 15 and 16; diet composition is detailed in ‘Diets’ in Methods). Surprisingly, on the first measure, males and females on Safe’s CD exhibited different blood glucose concentrations with 10.4 ± 0.4 and 7.9 ± 0.3 mM, respectively (mean ± s.e.m., Tukey test, *P* = 9.4 × 10^−6^), whereas after 1 month on Bio-Serv CD, glycemia stabilized at 10.3 ± 0.3 mM for males and 9.1 ± 0.5 mM for females (Tukey test, *P* = 0.99) (Fig. 4b and Supplementary Table 17). Glycemia remained stable on CD with for males, *K*_fast_ and *K*_slow_ rate constants of 0.1 and 4.9 × 10^−32^ per day, respectively, and HL_F_ and HL_S_ of 6.4 and 1.4 × 10^31^ days, respectively. For females, *K*_fast_ and *K*_slow_ were 8.4 × 10^−5^ per day, with HL_F_ and HL_S_ of 8.3 × 10^3^ days (Fig. 4c, Supplementary Fig. 7b and Supplementary Table 18). However, on KD, glycemia followed a two-phase decline, with a rapid early decrease characterized by *K*_fast_ of 4.6 and 2.5 per day and HL_F_ of 0.1 and 0.3 days for males and females, respectively, followed by a slower stabilization, with *K*_slow_ of 0.1 and 2.5 per day and HL_S_ of 7.4 and 0.3 days, for males and females, respectively (Fig. 4c, Supplementary Fig. 7b and Supplementary Table 18). After 4 weeks on KD, males and females experienced glycemic reductions of 50.9% ± 2.5% and 29.4% ± 10.1%, respectively, although the dispersions of the CD and KD female datasets were, respectively, 1.5 and 4 times more variable than the corresponding male groups with an s.d. of 32.5% for CD females compared with 22.0% for CD males, and 31.8% for KD females compared with 7.8% for KD males (Fig. 4d and Supplementary Table 19). In addition, females increased their glycemia on CD by the same proportion as males on CD (Tukey test, *P* = 0.74; Fig. 4d and Supplementary Table 19). RM ANOVA of the time course showed statistically significant differences between CD and KD diets for both males and females (Fig. 4a, *P* values in caption).

### Sex-specific β-HB responses to Bio-Serv diets

**Fig. 5:**
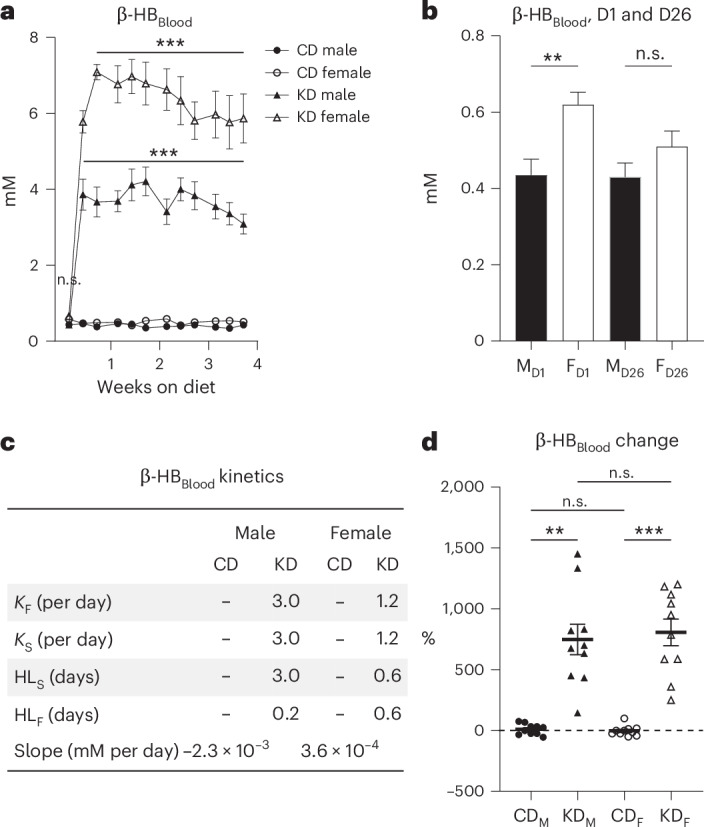
Blood β-HB analysis of mice on Bio-Serv chow. **a**, Blood β-HB (mM) in male (black symbols) and female (white symbols) mice fed CD (circles) or KD (triangles) over 1 month. RM ANOVA yields *P* = 9.9 × 10^−12^ between CD and KD diets for males and *P* = 1.6 × 10^−10^ between diets for females. **b**, Comparison of blood β-HB (mM) on day 1 (D1) and 26 (D26) between male (M, black bar) and female (F, white bar) mice. Day 1 data represent mice fed Safe’s CD, while day 26 data represent mice fed Bio-Serv CD. **c**, Kinetics parameters characterizing blood β-HB changes over the 4-week dietary intervention, including fast and slow decay constants (*K*_F_, *K*_S_, per day), half-lives fast and slow (HL_F_, HL_S_, days) and slopes (mM per day). **d**, Blood β-HB change (%) from the first to the last measurement in CD-fed males (black circle, CD_M_), KD-fed males (black triangle, KD_M_), CD-fed females (white circle, CD_F_), and KD-fed females (white triangle, KD_F_). *N* = 10 mice per diet group. Data are presented as mean ± s.e.m.; ***P < 0.001, **P < 0.01, not significant (n.s.), *P* > 0.05 (Supplementary Tables 20–24).

Blood β-HB levels, a key ketosis biomarker, were also monitored (Fig. 5a, Supplementary Figs. 10 and 11 and Supplementary Tables 20 and 21). During the acclimatization period, the β-HB level in males was 0.4 ± 0.04 mM, while it was significantly higher in females with 0.6 ± 0.03 mM (mean ± s.e.m., Fig. 5b; Tukey test, *P* = 1.2 × 10^−3^, and Supplementary Table 22). After 1 month on Bio-Serv CD, concentrations equalized at 0.4 ± 0.04 mM for males and 0.5 ± 0.04 mM for females (Tukey test, *P* = 0.26). On CD, β-HB remained stable, with minimal slope changes of −2.3 × 10^−3 ^mM per day for males and 3.6 × 10^−4 ^mM per day for females (Fig. 5c, Supplementary Fig. 7c and Supplementary Table 23). By contrast, on KD, both sexes experienced rapid β-HB increases, with, for males, *K*_fast_ and *K*_slow_ rate constants of 3.0 per day, HL_F_ of 0.2 days and HL_S_ of 3.0 days. For females, *K*_fast_ and *K*_slow_ were 1.2 per day, and HL_F_ and HL_S_ were 0.6 days (Fig. 5c, Supplementary Fig. 7c and Supplementary Table 23). Both sexes on KD had a similar β-HB increase of 747.2% ± 13.9% for males and 806.3% ± 109.3% for females (Fig. 5d; Tukey test, *P* = 0.96, and Supplementary Table 24). The β-HB data in KD males showed a ninefold higher variability than in CD males, with an s.d. of 397.3% compared with 44.1%, respectively. Similarly, β-HB variability in KD females was eightfold higher than in CD females, with an s.d. of 345.7% for KD females compared with 42.4% for CD females (Fig. 5d and Supplementary Table 24). Of note, on CD, basal β-HB level in females decreased by 2.7% ± 13.4%, whereas in males it increased by 9.5% ± 13.9% (Tukey test, *P* = 0.99), thereby rendering their systemic concentrations ultimately equivalent (Fig. 5b,d and Supplementary Tables 22 and 24). RM ANOVA of the time course showed statistically significant differences between CD and KD diets for both males and females (Fig. 5a, *P* value in caption).

### Blood lactate stability across Bio-Serv diets

**Fig. 6:**
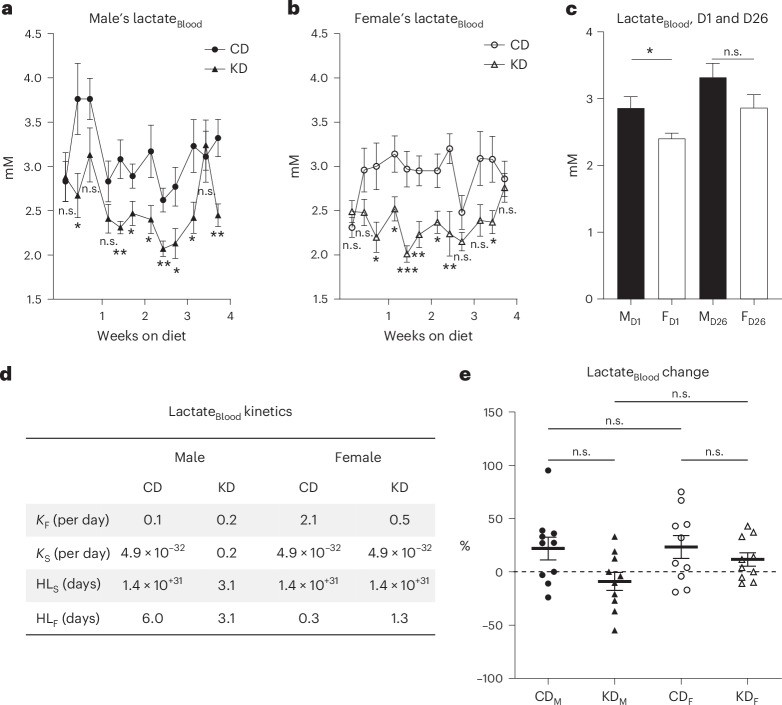
Blood lactate analysis of mice on Bio-Serv chow. **a**,**b**, Blood lactate (mM) in male (black symbols, **a**) and female (white symbols, **b**) mice fed CD (circles) or KD (triangles) over 1 month. RM ANOVA yields *P* = 3.5 × 10^−3^ between diets for males and *P* = 1.3 × 10^−4^ between diets for females. **c**, Comparison of blood lactate (mM) on days 1 (D1) and 26 (D26) between male (M, black bar) and female (F, white bar) mice. Day 1 data represent mice fed Safe’s CD, while day 26 data represent mice fed Bio-Serv CD. **d**, Kinetics parameters characterizing blood lactate changes over the 4-week dietary intervention, including fast and slow decay constants (*K*_F_, *K*_S_, per day), and half-lives fast and slow (HL_F_, HL_S_, days). **e**, Blood lactate change (%) from the first to the last measurement in CD-fed males (black circle, CD_M_), KD-fed males (black triangle, KD_M_), CD-fed females (white circle, CD_F_) and KD-fed females (white triangle, KD_F_). *N* = 10 mice per diet group. Data are presented as mean ± s.e.m.; ***P < 0.001, **P < 0.01, not significant (n.s.), *P* > 0.05 (Supplementary Tables 25–29).

On Safe’s CD, males displayed significantly higher levels of blood lactate with 2.9 ± 0.2 mM compared with females with 2.4 ± 0.1 mM (mean ± s.e.m.; Tukey test, *P* = 0.02, Fig. 6a–c, Supplementary Figs. 12 and 13 and Supplementary Tables 25 and 26). After 1 month on Bio-Serv CD, lactate concentrations were similar, with 3.3 ± 0.2 mM for males and 2.9 ± 0.2 mM for females (Fig. 6c; Tukey test, *P* = 0.28, and Supplementary Table 27). Lactate remained stable over time in CD males, with *K*_fast_ and *K*_slow_ rate constants of 0.1 and 4.9 × 10^−32^ per day, and HL_F_ and HL_S_ of 6.0 and 1.4 × 10^31^ days, respectively (Fig. 6a,d). For females, the concentration increased slightly with *K*_fast_ and *K*_slow_ of 2.1 and 4.9 × 10^−32^ per day, and HL_F_ and HL_S_ of 0.3 and 1.4 × 10^31^ days, respectively (Fig. 6a,d, Supplementary Fig. 7e,f and Supplementary Tables 25 and 28). On KD, lactate levels slowly decreased without significant changes for either sex after 4 weeks (Tukey test, *P* = 0.99). In males, *K*_fast_ and *K*_slow_ were 0.2 per day, and HL_F_ and HL_S_ were 3.1 days. For females, *K*_fast_ was 0.5 per day, *K*_slow_ 4.9 × 10^−32^ per day, HL_F_ 1.3 days, and HL_S_ 1.4 × 10^31^ days (Fig. 6d, Supplementary Fig. 7e,f and Supplementary Table 28). Their respective s.d. values were similar, with 33.9% for CD males, 26.9% for KD males, 33.8% for CD females and 19.8% for KD females (Fig. 6e and Supplementary Table 29). RM ANOVA of the time course showed a statistically significant difference between CD and KD diets for both males and females (Fig. 6a,b, *P* value in caption).

### Physiological impacts of the KD on neurons and astrocytes

**Fig. 7:**
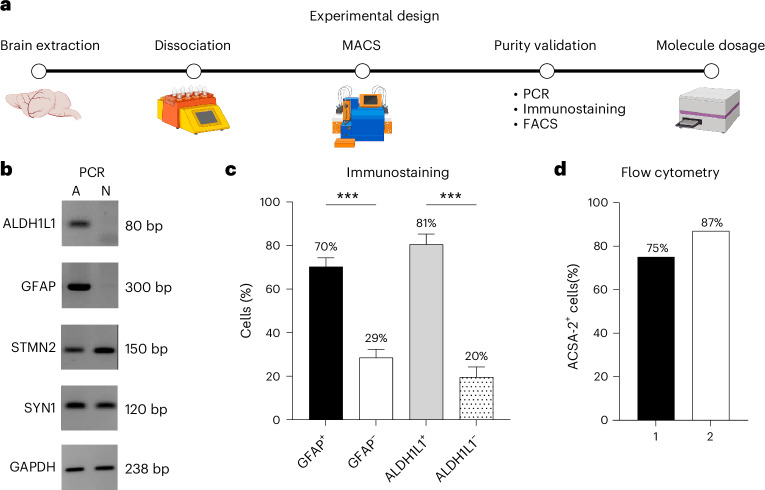
Neuroglial cell isolation and purity validation. **a**, Stepwise workflow illustrating the process for dissociating and isolating neurons and astrocytes of brains extracted from mice fed Bio-Serv CD or KD. The purity of the cell fractions obtained after magnetic-activated cell sorting (MACS), was controlled using PCR, immunostaining and flow cytometry analysis. Finally, multiple metabolite levels were measured in each cell population. Purity assessment of isolated astrocyte (A) and neuron (N) fractions. FACS, fluorescence-activated cell sorting. **b**, PCR analysis of specific glial mRNAs, ALDH1L1 (80 bp) and GFAP (300 bp), and neurons mRNAs, STMN2 (150 bp) and SYN1 (120 bp). Glyceraldehyde-3-phosphate dehydrogenase (GAPDH, 238 bp) served as a positive control. **c**, Immunostaining assessment of the extent of neuronal contamination in astrocyte fractions by quantifying the percentage of cells positively (+) and negatively (−) stained for GFAP (GFAP^+^, GFAP^−^) and ALDH1L1 (ALDH1L1^+^, ALDH1L1^−^) relative to the total cell population within the ROI (*x* axis). **d**, Flow cytometry analysis confirmed immunostaining results by quantifying cells positively stained with the ACSA-2 antibody (ACSA-2^+^) in the astrocytic fraction. For PCR, *N* = 1 for both astrocyte (A) and neuron (N) fractions. For immunostaining, four ROIs from two samples were analyzed. Data are presented as mean ± s.e.m.; ***P < 0.001 (Supplementary Table 30). For flow cytometry analysis, two samples were analyzed. Illustrations created in BioRender; Lorin, C. https://biorender.com/o7d9y2o (2026).

After validating Bio-Serv CD and KD in C57BL/6J mice as effective for sustaining ketosis, the KD’s effects on neuroglial cells were examined for potential sex differences. Brain cells were dissociated from mice fed CD or KD for 1 month (Fig. 7a) and sorted into astrocyte and neuron populations, with purity analyzed by polymerase chain reaction (PCR) (Fig. 7b). The astrocyte (A) fraction contained glial messenger ribonucleic acids (mRNAs) aldehyde dehydrogenase 1 family member L1 (ALDH1L1) and glial fibrillary acidic protein (GFAP), but was contaminated with neuronal mRNAs stathmin 2 (STMN2) and synapsin I (SYN1). Conversely, the neuron (N) samples displayed only STMN2 and SYN1 mRNAs (Fig. 7b). To further quantify the contamination in the astrocytic fractions, immunostaining and flow cytometry analysis were performed. GFAP and ALDH1L1 immunostaining showed that 70–81% of the A fraction were astrocytes (Fig. 7c, Supplementary Fig. 14 and Supplementary Table 30), confirmed by flow cytometry analysis using the astroglia-specific marker astrocyte cell surface antigen-2 (ACSA-2; Fig. 7d and Supplementary Fig. 15). On average, 77% of the cells in the glial extract were identified as astrocytes.

**Fig. 8:**
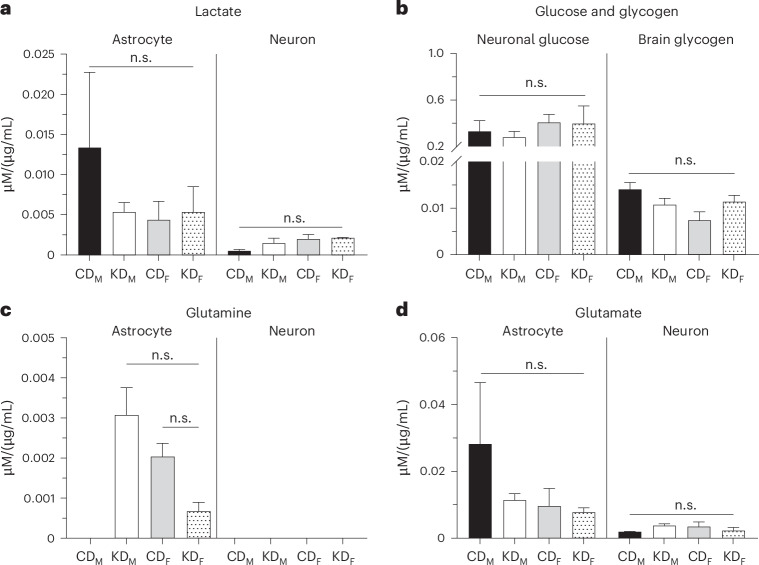
Metabolite levels in neuroglial cells from mice fed Bio-Serv chow. **a**, Lactate levels in astrocytes and neurons. In astrocytes, Dunn’s multiple comparison test yields *P* = 0.9 for CD-fed males (CD_M_, black bar) versus KD-fed males (KD_M_, white bar), CD_M_ versus CD-fed females (CD_F_, gray bar), CD_M_ versus and KD-fed females (KD_F_, dotted bar), KD_M_ versus CD_F_, KD_M_ versus KD_F_, and CD_F_ versus KD_F_. In neurons, Dunn’s multiple comparison test yields *P* = 0.9 for CD_M_ versus KD_M_, *P* = 0.3 for CD_M_ versus CD_F_, *P* = 0.1 for CD_M_ versus KD_F_, and *P* = 0.9 for KD_M_ versus CD_F_, KD_M_ versus KD_F_, and CD_F_ versus KD_F_. **b**, Neuronal glucose and brain glycogen levels. In neurons, Dunn’s multiple comparison test yields *P* = 0.9 for CD_M_ versus KD_M_, CD_M_ versus CD_F_, CD_M_ versus KD_F_, KD_M_ versus CD_F_, KD_M_ versus KD_F_, and CD_F_ versus KD_F_. In brains, Dunn’s multiple comparison test yields *P* = 0.9 for CD_M_ versus KD_M_, *P* = 0.1 for CD_M_ versus CD_F_, and *P* = 0.9 for CD_M_ versus KD_F_, KD_M_ versus CD_F_, KD_M_ versus KD_F_, and CD_F_ versus KD_F_. **c**, Glutamine levels in astrocytes and neurons. In astrocytes, Dunn’s multiple comparison test yields *P* = 0.9 for KD_M_ versus CD_F_, KD_M_ versus KD_F_, and CD_F_ versus KD_F_. **d**, Glutamate levels in astrocytes and neurons. In astrocytes and neurons, Dunn’s multiple comparison test yields *P* = 0.9 for CD_M_ versus KD_M_, CD_M_ versus CD_F_, CD_M_ versus KD_F_, KD_M_ versus CD_F_, KD_M_ versus KD_F_, and CD_F_ versus KD_F_. All metabolite concentrations were normalized to protein content in each sample (µM/(µg/mL)). *N* = 3 samples per experimental condition, except for neuronal lactate, glucose, glutamine and glutamate from KD-fed males *N* = 2. Data are presented as mean ± s.e.m.; not significant (n.s.), *P* > 0.05 (Supplementary Tables 31–34).

Subsequently, metabolite concentrations were analyzed to assess KD and sex effects within each brain cell type. In astrocytes and neurons, Dunn’s multiple comparison did not show a statistically significant difference in lactate levels between CD and KD diets for both males and females (Fig. 8a, *P* value in caption, and Supplementary Table 31). In astrocytes, CD males and females had 1.3 × 10^−2^ ± 9.4 × 10^−3 ^µM/(µg/mL) and 4.3 × 10^−3^ ± 2.3 × 10^−3 ^µM/(µg/mL) of lactate, respectively, while KD males contained 5.3 × 10^−3^ ± 1.2 × 10^−3 ^µM/(µg/mL) and females 5.3 × 10^−3^ ± 3.2 × 10^−3 ^µM/(µg/mL) (mean ± s.e.m., *P* values in captions, Fig. 8a, Supplementary Fig. 16a and Supplementary Table 31). On CD neurons, males had 5.0 × 10^−4^ ± 2.0 × 10^−4 ^µM/(µg/mL) of lactate, and females 2.0 × 10^−3^ ± 5.9 × 10^−4 ^µM/(µg/mL). On KD, males held 1.5 × 10^−3^ ± 6.5 × 10^−4^, and females 2.1 × 10^−3^ ± 6.7 × 10^−5 ^µM/(µg/mL). On average, both sexes combined, lactate concentrations were 4.5 times higher in astrocytes than in neurons, with 8.8 × 10^−3^ ± 4.8 × 10^−3^ on CD, and 5.3 × 10^−3^ ± 1.5 × 10^−3 ^µM/(µg/mL) on KD in astrocytes, compared with 1.2 × 10^−3^ ± 4.3 × 10^−4^ on CD, and 1.9 × 10^−3^ ± 2.7 × 10^−4 ^µM/(µg/mL) on KD in neurons (data not shown).

Interestingly, glucose was detected only in neurons, with similar levels across sexes and diets. CD males had 0.3 ± 0.1 µM/(µg/mL) and females 0.4 ± 0.1 µM/(µg/mL), and KD males contained 0.3 ± 0.1 µM/(µg/mL) and females 0.4 ± 0.2 µM/(µg/mL) (Fig. 8b, left, *P* value in caption, Supplementary Fig. 16b and Supplementary Table 32). Likewise, the glycogen level measured in the total brain tissue was of 1.4 × 10^−2^ ± 1.5 × 10^−3 ^µM/(µg/mL) in CD males, 7.3 × 10^−3^ ± 1.9 × 10^−3 ^µM/(µg/mL) in CD females, and 1.1 × 10^−2^ ± 1.5 × 10^−3 ^µM/(µg/mL) in KD males and females (Fig. 8b, left, *P* value in caption, Supplementary Fig. 16b,c and Supplementary Table 32).

Glutamine, present only in astrocytes, was undetected in CD males but found in females with 2.0 × 10^−3^ ± 3.3 × 10^−4 ^µM/(µg/mL) and in KD males with 3.1 × 10^−3^ ± 6.9 × 10^−4 ^µM/(µg/mL), which was significantly higher than in KD females with only 6.7 × 10^−4^ ± 2.3 × 10^−4 ^µM/(µg/mL) (Fig. 8c, Supplementary Fig. 16d and Supplementary Table 33). In astrocytes, Dunn’s multiple comparison did not show a statistically significant difference in glutamine levels between CD and KD diets for both males and females (Fig. 8c, *P* value in caption, and Supplementary Table 33).

Glutamate was higher in astrocytes than in neurons with 2.8 × 10^−2^ ± 1.8 × 10^−2 ^µM/(µg/mL) in CD males, 9.6 × 10^−3^ ± 5.3 × 10^−3 ^µM/(µg/mL) in CD females, 1.1 × 10^−2^ ± 2.0 × 10^−3 ^µM/(µg/mL) in KD males, and 7.7 × 10^−3^ ± 1.4 × 10^−3 ^µM/(µg/mL) in KD females (Fig. 8d, left, Supplementary Fig. 16e and Supplementary Table 34). Conversely, in neurons, CD males had 1.9 × 10^−3^ ± 1.5 × 10^−4 ^µM/(µg/mL), CD females 3.4 × 10^−3^ ± 1.5 × 10^−3 ^µM/(µg/mL), KD males 3.8 × 10^−3^ ± 5.5 × 10^−4 ^µM/(µg/mL) and KD females 2.3 × 10^−3^ ± 9.6 × 10^−4 ^µM/(µg/mL) (Fig. 8d, right, Supplementary Fig. 16e and Supplementary Table 34). On average, the glutamate level was higher in astrocytes with 1.4 × 10^−2^ ± 5.5 × 10^−3 ^µM/(µg/mL) compared with neurons with 2.8 × 10^−3^ ± 7.0 × 10^−4 ^µM/(µg/mL) (data not shown). In astrocytes and neurons, Dunn’s multiple comparison did not show a statistically significant difference in glutamate levels between CD and KD diets for both males and females (Fig. 8d, *P* value in caption, and Supplementary Table 34). Overall, due to the limited number of samples available to analyze each metabolite, further validation will be needed.

## Discussion

Most KD studies use male animals without justifying excluding females^57,58^, even though both men and women worldwide follow this diet^59^. Including both sexes in research ensures reliable and accurate findings^11,60^. However, comparing results between KD studies can be challenging due to differences in animal models, strains and diet composition^48–52,61^. Given the importance of reproducibility in scientific research for result validation, insight discoveries and biomedical research translation, uniformity in KD studies is critical. This study aimed to establish a standardized mouse model sustaining ketosis over long periods using a quality KD. Consistent with previous reports, our data confirm that diet composition and formulation critically influence the ability to induce and maintain ketosis and extend prior work by demonstrating pronounced sex-dependent systemic responses under standardized conditions^26,57,62–64^. C57BL/6J mice were selected for their identical genetics and phenotypes, as confirmed by our findings of consistent BW across individuals. The use of this common strain minimizes disparities and facilitates comparisons with other studies.

The next step was selecting a KD. Safe was chosen as the primary diet supplier owing to its routine use in our facility, with Bio-Serv, a cited provider in the literature, tested for comparison. Bio-Serv’s CD contained slightly more carbohydrates and fewer proteins and lipids, but both CDs were classic pellets, placed in conventional trays of housing cages. Safe’s KD was a hard block, while Bio-Serv’s was a creamy paste. Both were placed in feeders on the cage bedding. The different textures and placements compared with the CD could affect behavior, perhaps reducing climbing^51,65–69^. Indeed, the paste KD persistently greased the animals’ fur and led to reduced grooming^70^. In addition, its consistency prevented food hiding, as it was absorbed by the litter^68^, and lessened gnawing, potentially causing malocclusion and malnourishment^71^. While no dental issues were observed during the 1-month study, longer interventions should include proper enrichments such as wood blocks, cardboard tubes or coconut shells to prevent such complications and ensure unbiased results^72,73^. Furthermore, while the declared saturated FA content of the Safe’s KD indicates that all saturated FAs are long-chain, the exact ratios of individual FAs in Bio-Serv’s KD were not reported. Differences in the ratio of specific FA, along with variations in diet texture and form, may influence metabolic and behavioral outcomes. In addition, even though food intake was not directly measured in this study, we acknowledge that caloric consumption may influence metabolic outcomes, and that sex-related differences in feeding behavior could have contributed to the observed variation in ketone production. Accurate quantification of food intake was challenging due to the paste-like texture and high lipid content of KD, which caused the food to soften or melt upon contact with the animals’ warm body and fur, resulting in unquantifiable losses. Future studies may benefit from refined approaches to assess food intake while maintaining animal welfare, for example, by presenting the chow in ceramic containers for limited periods to reduce food loss and enable more precise measurement, or comparing powdered versus pelleted CDs^51^.

Safe’s KD successfully induced ketosis, as indicated by decreased BW, lower glycemia and increased β-HB levels^57^. Moreover, blood lactate remained stable, suggesting a preference for ketone utilization over glucose metabolism and indicating an adaptation to the ketogenic nutritional state. However, after 2 weeks, these parameters unexpectedly reversed. Upon investigation, stratifications in the Safe’s KD within the original container were identified, with a top yellow layer presumably rich in lipids and a lower brown layer probably containing higher levels of carbohydrates and proteins. The issue was that the fresh food pieces provided to the mice were not composed of both layers simultaneously. Initially, only pieces from the top of the container were used, consisting solely of the yellow layer. Over time, as this yellow layer was depleted, and the underlying brown layer became exposed and subsequently distributed to the animals; this occurred approximately 2 weeks after the container was first started. Because the food was portioned and replaced weekly from the same container, these stratifications were present from the beginning of each feeding period rather than developing over time within the cage environment. Importantly, this suggests that the observed metabolic shift was not due to oxidation or environmental conditions but rather to an inherent inconsistency in the pre-prepared diet. However, as the exact composition of these layers was not analyzed, further validation is needed to confirm these observations. A potential alternative would have been to homogenize the food before distribution, but this was not feasible as the issue was identified only after data analysis. While the manufacturer declared this food production issue to be exceptional, it underscores the necessity for stringent quality controls to ensure diet homogeneity before and during experimental interventions^74,75^. By contrast, Bio-Serv’s KD maintained ketosis throughout the study, making it a more reliable choice for establishing a standardized ketosis model.

Sex-based metabolic differences were observed. At baseline, on Safe’s CD, females were leaner, with lower glycemia and higher blood β-HB, probably influenced by sex-specific hormone differences. Estrogen favors subcutaneous fat deposition, which is easier to lose than visceral fat, predominant in males^75,76^, protects β-cell from apoptosis, stimulates insulin secretion and sensitivity^77–79^, and promotes FA β-oxidation, contributing to increased KB production^40,41,43–45^. However, after 1 month on Bio-Serv’s CD, the sex differences in glycemia and β-HB levels decreased, probably due to slight variations in macronutrient composition between the two CD formulations, with Bio-Serv containing 3% more carbohydrates and 1% fewer lipids than Safe.

On Bio-Serv’s KD, both males and females exhibited similar BW kinetic and loss. While this suggests comparable overall physiological adaptations, it does not necessarily imply identical underlying molecular mechanisms. Previous works have attributed KD-related weight loss to appetite reduction, visceral fat reduction, lean mass preservation and increased resting metabolic rate^26,62–64^. However, body composition was not assessed, which is a limitation. Future studies should include this analysis to determine the extent to which lean or fat mass contributes to weight changes observed under KD. Notably, higher interindividual variability in female BW data suggests that the estrous cycle may influence weight loss. Because the females in our study were not hormonally synchronized, metabolic responses to KD may have varied depending on cycle stage at diet initiation^36–40^. Estrogen inhibits lipogenesis and promotes FA oxidation, enhancing KB production^40,41^, whereas progesterone reduces KB synthesis by favoring TG generation^40,42^. This hormonal interplay may have contributed to the observed variability. Future studies involving hormonal synchronization or estrous cycle monitoring are necessary to confirm these effects^80^. Sexual dimorphism was also evident in the glycemic response to KD. Females experienced a faster, yet less pronounced, glucose drop, probably due to estrogen’s effect on insulin sensitivity^81^. In addition, variability in glycemia data may have resulted from ad libitum feeding, leading to differences in postprandial timing for measurements. Implementing a time-restricted feeding regimen or performing fasting blood glucose measurements could help standardize glucose assessments in future studies, reducing confounding effects, particularly given that KD inherently mimics a fasting state^82,83^. Regarding β-HB production, females exhibited a faster increase in response to KD, although both sexes reached comparable levels by the end of the study. In addition to estrous cycle fluctuations, individual differences in hepatic adaptation to ketosis could explain this variation^36–42^. Additional research is needed to better understand sex-specific liver adaptations to KD. Beyond metabolic parameters, behavioral changes between diets were not assessed, which represents another limitation. KD can influence activity levels, mood and cognitive function, which are important considerations when selecting an optimal dietary model. Future studies should include behavioral assessments to provide a more comprehensive understanding of KD’s impact on animal well-being and function. Overall, our study demonstrated that C57BL/6J mice fed Bio-Serv KD successfully achieved sustained ketosis, supporting its efficacy as a reliable dietary model for metabolic studies^84,85^. However, future research addressing body composition, behavioral outcomes, hormonal synchronization and molecular mechanisms underlying sex-specific responses to KD will be essential for refining our understanding of metabolic sex differences in ketogenic adaptations.

Following diet intervention, astrocytes and neurons were isolated from the mouse brains to analyze key metabolic molecules^86^. Lactate, an essential neuronal energy source^87^, exhibited a gradient from astrocytes to neurons, reflecting higher levels in astrocytes relative to neurons, consistent with the astrocyte-to-neuron lactate shuttling, well described in other findings^88^, and validating our analytical method. However, the limited sample size of our study precludes definitive conclusions. Glucose was detected exclusively in neurons, where it was transported from the interstitium to generate ATP^88,89^, while in astrocytes, it rapidly entered glycolysis or was stored as glycogen. Although this distribution is consistent with established cellular metabolic specialization, we cannot exclude the possibility that rapid astrocytic glucose utilization or technical limitations during cell isolation may have contributed to the apparent absence of detectable glucose in astrocytes. Interestingly, despite the marked differences in dietary carbohydrate intake between CD and KD, neuronal glucose levels were comparable between CD- and KD-fed animals. One potential explanation is the activation of gluconeogenesis under low-carbohydrate conditions, which may sustain a minimal but steady glucose supply through endogenous synthesis from lactate, glycerol (from TG breakdown) and glucogenic amino acids such as alanine and glutamine^90,91^. Glycogen levels, measured directly in brain tissues, reflected astrocytic content^92^. Similarly, glutamine was found only in astrocytes, aligning with its established role in glutamate synthesis^93^. However, as with glucose, we cannot exclude that technical factors related to cell isolation, metabolite turnover or detection sensitivity may have influenced the apparent cell-type specificity of glutamine. Glutamate was measured at higher concentrations in astrocytes, despite neurons typically containing ten times more^94^. This discrepancy may stem from experimental cell isolation^95^, which can lead to synaptic terminal loss and extracellular glutamate release. Importantly, no significant sex- or diet-related differences were observed in brain cell metabolism. Despite systemic metabolic differences identified in whole-tissue analyses, cerebral metabolism appeared stable across conditions, suggesting a centrally regulated mechanism that maintains essential brain functions independently of dietary or hormonal influences.

This study successfully validated an animal model of sustained, stable and healthy ketosis. Our findings highlight systemic sexual dimorphism, probably driven by sex steroid hormones, reinforcing the necessity of including female subjects in KD studies. The absence of significant metabolic differences in isolated brain cells suggests that sex-specific systemic adaptations may be counterbalanced at the cerebral level, ensuring functional stability. A deeper understanding of sex-specific physiological responses could inform personalized dietary strategies, particularly in medical treatments. Future studies with larger sample sizes and advanced methodologies are warranted to confirm these findings and further explore the mechanisms governing brain metabolic homeostasis.

## Methods

### Animals

A total of 60 healthy C57BL/6J mice (strain code: 632, Charles River Laboratories), males (*N* = 40) and females (*N* = 20), of 8-week-old, were used to investigate the impact of the KD. Mice of equivalent weight were housed in groups of three to four per cage and maintained on a 12-h light–dark cycle with ad libitum access to food and water. No animals were excluded from the study. Registered in a license (national permit number 33876), all procedures were conducted following the Swiss Welfare Act, and the Swiss National Institutional and Veterinary Office guidelines in the Canton of Vaud on Animal Experimentation.

### Diets

KD and CD were sourced from Safe (Safe-Lab, www.safe-lab.com) and Bio-Serv (www.bio-serv.com). Food was replaced weekly. After 2 weeks of acclimatization on Safe’s CD (the food commonly used in the animal facility), with gentle handling and restrained habituation, the mice were assigned to either KD or CD groups from Bio-Serv or Safe for a 4-week feeding period. Mice were subjected to weighing and blood tests three times a week (Supplementary Fig. 17).

The CD was composed of carbohydrates, proteins and lipids as follows (kcal%) (Supplementary Fig. 18a–e):

- Bio-Serv: 69, 19 and 12 (F1515-V).
- Safe: 66, 21 and 13 (Safe 150).

The CD was pellets placed in the food tray of housing cages.

The KD was composed as follows (kcal%):

- Bio-Serv: 2, 5 and 93 (F3666-V).
- Safe: 3, 9 and 89 (Safe U8954 version 176).

Bio-Serv KD was provided as fat paste in glass feeders, while Safe’s KD was more compact (Supplementary Fig. 18f–h) and provided as chunks in plastic feeders. Both disposals were covered with a stainless-steel shield (Unifab corporation) and placed on the cage bedding.

### Blood biochemical analysis

Glycemia, β-HB and lactate were measured in blood three times per week using the tail-tip excision method (Supplementary Fig. 17). The animals were maintained on a food tray to clip the distal part of the tail with sharp scissors. The resulting blood drops were collected on specific reader strips connected to a glucometer (Contour XT, Bayer), lactate meter (StatStrip Xpress, Nova biomedical) or β-HB meter (Freestyle Precision Neo, Abbott).

### Brain dissociation

Mice were rapidly anesthetized with isoflurane (5% in oxygen) in a gas chamber and decapitated. Then, brains were collected and placed in ice-cold phosphate-buffered saline (PBS) before being dissociated into single-cell suspensions using the Adult Brain Dissociation Kit (130-107-677, Miltenyi).

### Magnetic-activated cell sorting

After dissociation, astrocytes were labeled using the ACSA-2 antibody (1:10; 130-097-678, Miltenyi)^96^, and neurons were isolated via negative selection (130-115-389, Miltenyi).

### PCR

RNA extraction from isolated cells was performed using RNeasy Mini Kit (74104, Qiagen). Complementary deoxyribonucleic acid (cDNA) was synthesized using Superscript III (18080044, Invitrogen). PCRs were carried out on a ProFlex PCR system (Applied Biosystems) using a GoTaq PCR master mix that contained GoTaq Green buffer (M7805, Promega), 10 mM dNTP (U1515, Promega), 10 µM forward primer, 10 µM reverse primer, GoTaq (M7805, Promega), 1:50 cDNA and double distilled water (ddH_2_O). The following cycles were performed: initial denaturation cycle at 95 °C for 3 min, followed by 40 amplification cycles of 95 °C for 40 s, 50 °C for 40 s, and 72 °C for 45 s, and ending with one cycle at 72 °C for 5 min. Finally, PCR products were analyzed on a 2% agarose gel (11405.01, Serva) in TBE 1× (89 mM Tris, A 1086.5000, Applichem; 89 mM boric acid, A 2940.1000, Applichem; 2 mM ethylenediamine tetraacetic acid (EDTA), A 1103.0500, Applichem) at 75 V. All primer sequences are listed in Supplementary Table 35 (‘PCR primers’) and were designed using NCBI Primer-BLAST^86^.

### Immunocytochemistry

Astrocytes (5 × 10^4^) were plated on poly-d-lysine-coated glass coverslips (CB00180RAC20MNZ0, diameter 18 mm, #1,5, Epredia) (poly-d-lysine: 50 µg/mL in ddH_2_O, A38904-01, Gibco) in culture dishes (353803, Corning Life Sciences) with D-phosphate buffer solution (D-PBS, 14040-133, Gibco) containing 0.5% MACS bovine serum albumin (130-091-376, Miltenyi). After 2 h at 37 °C with 5% CO_2_, cells were fixed with 4% paraformaldehyde for 30 min at room temperature (RT). Following washes in PBS, cells were blocked and permeabilized with a 3% normal goat serum (NGS, S-1000-20, Vector Laboratories), and 0.5% Triton X-100 (A4975, AppliChem) in PBS for 1 h at RT. Primary antibodies were prepared in 0.04% Triton X-100 and 2% NGS in PBS: mouse anti-GFAP (1:100; MAB360, Merck) and rabbit anti-aldehyde ALDH1L1 (1:500; ab87117, Abcam), and incubated with cells overnight at 4 °C. After washes in PBS, cells were incubated for 2 h at RT with secondary antibodies: goat anti-mouse Alexa Fluor 488 (1:1000; A10680, Invitrogen) and donkey anti-rabbit Alexa Fluor 647 (1:1000; ab150075, Abcam) in 0.04% Triton X-100 and 2% NGS in PBS. Then, washes in PBS were performed before incubation for 10 min in 0.04% Triton X-100 and 2% NGS in PBS containing 4′,6-diamidino-2-phenylindole (DAPI, 1:50,000, D9542, Sigma). Finally, after washes, cells were mounted in fluorescence mounting medium (S3023, Dako) on Superfrost Plus adhesion slides (J1800AMNZ, Thermo Scientific), dried overnight and stored at 4 °C until microscopy acquisition and analysis. Negative controls included secondary antibodies only (data not shown).

### Confocal microscopy

An upright confocal microscope (Upright Leica SP8 DM6 CS), equipped with a 20× (0.75 numerical aperture (NA), HC PL APO air objective (Leica) was used. Images were captured in the *xy* plane, with dimensions fixed at 2,704 × 2,704 and a zoom factor of 1, resulting in a pixel size of 215.04 nm.

DAPI, Alexa Fluor 488, and 647 were excited to 405, 488 and 638 nm, respectively. The laser speed was 400 Hz with a four-frame average per channel. The emitted fluorescences were sequentially detected by two HyD and one photomultiplier tube spectral detectors tuned to the 410–480 nm, 500–550 nm and 640–700 nm wavelengths, respectively. The acquired images were visualized using Leica Application Suite X software (Leica).

For each of the two samples of cells investigated, four regions of interest (ROIs) were randomly chosen across the coverslips.

### Image processing

To count the number of cells in each ROI, the channels of each antibody, including DAPI, GFAP and ALDH1L1, were split into three separate images in Fiji-ImageJ software. The DAPI pictures were filtered using the automatic threshold ‘Moments’^97^, discarding noise exceeding the segmentation threshold. Then, the number of nuclei was counted in the segmented image with the ‘Analyze Particles’ function set as follows: 30-Infinity for the size (micron^2^), pixel units, circularity between 0.00 and 1.00, and ‘outlines’ to emphasize the nuclei. Next, the number of cells co-expressing GFAP–DAPI and ALDH1L1–DAPI was manually counted and visually controlled by a trained experimenter.

### Flow cytometry analysis

Cell viability was assessed with propidium iodide and analyzed in the phycoerythrin (PE) channel (585/42) using a four-laser Cytoflex S (Beckman Coulter). The cell survival rate was estimated at 85%, on average (data not shown).

Astrocytic fraction purity after MACS was assessed using mouse anti-ACSA-2 conjugated to PE (1:50, 130-116-244, Miltenyi). After washes in PBS, the extract was centrifuged at 300*g* for 10 min, resuspended in PBS and sorted using AutoMACS Pro Cell separator from Miltenyi Biotec. Data analysis used CytExpert Software from Beckman Coulter (V 2.5.0.77).

### Metabolite detection

We used a Glycogen Assay Kit to assess glycogen levels via fluorometric detection (ab65620, Abcam). Lactate, glucose, glutamine and glutamate were measured using specific kits (lactate-J5021, Glucose-J6021, Glutamine/Glutamate-J8021, Promega). Metabolite concentrations were calculated from standard curves and normalized to protein levels measured with the Pierce 660 nm Protein Assay (22662, Thermo Scientific). All assays were read using the FlexStation 3 microplate reader (Molecular Devices).

### Blinding

Cages were assigned identification numbers so that the experimenter could not deduce the diet of the mice. For the chemistry analysis, the experimenter was blind to the study design.

### Sample size calculation

The sample size was calculated based on the difference in blood ketone levels between KD and KD ground reported in the literature^28^. An alpha level of 0.05, a power of 0.8 and a large effect size of 0.8, as defined by Cohen’s *d*, were used. According to G*Power software (Heinrich Heine Universität), ten mice per group was the estimated number of animals per diet group to successfully achieve adequate statistical power and provide data for this project.

### Statistical analysis

GraphPad Prism and R were used to analyze the data. Detailed descriptions are available in Supplementary Table 1. Data are presented in scatter dot plots and histograms as mean ± s.e.m. For comparisons at specific time points, two-way ANOVA with a Tukey test post-hoc analysis was performed to obtain *P* values. RM ANOVA was used to compare treatments in which time series were measured under different treatment regimes. Dunn’s multiple comparison test, a post-hoc nonparametric test run after a Kruskal–Wallis test, was used to compare neuroglial cells and brain tissues under different diets in males and females. Statistical significance was defined as follows: not significant (n.s.) *P* > 0.05, *P < 0.05, **P < 0.01 and ***P < 0.001.

The analyses of kinetic rate constants (*K*_fast_ and *K*_slow_), half-life_fast_ and half-life_slow_ (HL_F_ and HL_S_) for BW, glycemia and blood lactate were performed using nonlinear regression analysis, using a double exponential decay model. Specifically, a two-phase model was applied, where the result is the sum of a fast and slow exponential decay.

The model can be expressed as follows:

- SpanFast = (Y0-Plateau)*PercentFast*.01
- SpanSlow = (Y0-Plateau)*(100-PercentFast)*.01
- Y=Plateau + SpanFast*exp(-KFast*X) + SpanSlow*exp(-KSlow*X)

For blood β-HB, the analyses were similarly conducted with a nonlinear regression in two-phase association using the following model:

- SpanFast = (Plateau-Y0)*PercentFast*.01
- SpanSlow = (Plateau-Y0)*(100-PercentFast)*.01
- Y = Y0+ SpanFast*(1-exp(-KFast*X)) + SpanSlow*(1-exp(-KSlow*X))

*K*_fast_ and *K*_slow_ are the rate constants for the fast and slow phases, respectively, and are expressed in reciprocal time units (per day). Smaller values of *K* indicate slower changes in the parameter, while larger values indicate faster changes. HL_F_ and HL_S_ represent the time required for the measured parameter to decrease by half. They are in the time units of the *x* axis (days). They are computed as ln(2)/*K*. A larger half-life indicates slower changes in the parameter, while a smaller half-life suggests more rapid variation.

### Reporting summary

Further information on research design is available in the Nature Portfolio Reporting Summary linked to this article.

## Online content

Any methods, additional references, Nature Portfolio reporting summaries, source data, extended data, supplementary information, acknowledgements, peer review information; details of author contributions and competing interests; and statements of data and code availability are available at 10.1038/s41684-026-01732-7.

## Supplementary information

Supplementary Information Supplementary Figs. 1–18 and Tables 1–35.

Reporting Summary

## Data Availability

All data supporting the findings of this study are available within the article and its Supplementary Information.
