# Supplementary material for: Sex-specific systemic and brain metabolic responses to a standardized ketogenic diet in mice
Source: Lab Anim (NY). 2026 May 12;55(6):230–40. doi: 10.1038/s41684-026-01732-7 (PMC13233310; doi:10.1038/s41684-026-01732-7)
Supplement: Supplementary file 1 — Supplementary Figs. 1–18 and Tables 1–35. [file 41684_2026_1732_MOESM1_ESM.pdf]

https://doi.org/10.1038/s41684-026-01732-7

Sex-specific systemic and brain metabolic responses to a standardized ketogenic diet in mice

In the format provided by the authors and unedited

A

Test for normal distribution of CD male BW datasets

D'Agostino & Pearson Test	CD 1	CD 3	CD 5	CD 8	CD 10	CD 12	CD 15	CD 17	CD 19	CD 22	CD 24	CD 26
K2	0.8	1.4	0.7	1.0	0.9	0.7	0.3	0.9	1.7	0.3	0.8	0.7
P value	0.7	0.5	0.7	0.6	0.7	0.7	0.8	0.6	0.4	0.9	0.7	0.7
Passed normality test (alpha=0.05)?	Yes	Yes	Yes	Yes	Yes	Yes	Yes	Yes	Yes	Yes	Yes	Yes
P value summary	ns	ns	ns	ns	ns	ns	ns	ns	ns	ns	ns	ns
Number of values	10	10	10	10	10	10	10	10	10	10	10	10

QQ Plot

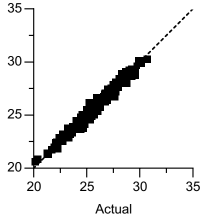

B

Test for normal distribution of KD male BW datasets

D'Agostino & Pearson Test	KD 1	KD 3	KD 5	KD 8	KD 10	KD 12	KD 15	KD 17	KD 19	KD 22	KD 24	KD 26
K2	0.2	4.2	2.4	3.3	2.4	4.4	2.8	4.5	7.1	3.2	2.9	9.4
P value	0.9	0.1	0.3	0.2	0.3	0.1	0.2	0.1	0.03	0.2	0.2	0.01
Passed normality test (alpha=0.05)?	Yes	Yes	Yes	Yes	Yes	Yes	Yes	Yes	No	Yes	Yes	No
P value summary	ns	ns	ns	ns	ns	ns	ns	ns	*	ns	ns	**
Number of values	10	10	10	10	10	10	10	10	10	10	10	10

QQ Plot

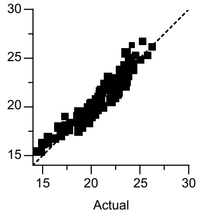

Supplementary Figure 1. Normality tests for Figure 2A datasets.

A), and B) (left panels) Tables of the statistical normality test D'Agostino-Pearson omnibus performed on datasets derived from Figure 2A. (Right panels) Quantile-quantile (QQ) normality plots resulting from this normality test. Control diet (CD), ketogenic diet (KD), body weight (BW).

A

Test for normal distribution of CD male glycemia datasets

D'Agostino & Pearson Test	CD 1	CD 3	CD 5	CD 8	CD 10	CD 12	CD 15	CD 17	CD 19	CD 22	CD 24	CD 26
K2	1.0	0.0	1.7	0.1	1.0	0.4	0.1	2.5	4.1	0.1	1.2	0.4
P value	0.6	>0.99	0.4	0.9	0.6	0.8	1.0	0.3	0.1	1.0	0.5	0.8
Passed normality test (alpha=0.05)?	Yes	Yes	Yes	Yes	Yes	Yes	Yes	Yes	Yes	Yes	Yes	Yes
P value summary	ns	ns	ns	ns	ns	ns	ns	ns	ns	ns	ns	ns
Number of values	10	10	10	10	10	10	10	10	10	10	10	10

QQ Plot

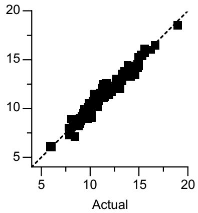

B

Test for normal distribution of KD male glycemia datasets

D'Agostino & Pearson Test	KD 1	KD 3	KD 5	KD 8	KD 10	KD 12	KD 15	KD 17	KD 19	KD 22	KD 24	KD 26
K2	2.4	3.0	2.3	2.7	2.3	1.9	5.1	3.4	0.8	1.0	0.6	1.9
P value	0.3	0.2	0.3	0.3	0.3	0.4	0.1	0.2	0.7	0.6	0.7	0.4
Passed normality test (alpha=0.05)?	Yes	Yes	Yes	Yes	Yes	Yes	Yes	Yes	Yes	Yes	Yes	Yes
P value summary	ns	ns	ns	ns	ns	ns	ns	ns	ns	ns	ns	ns
Number of values	10	10	10	10	10	10	10	10	10	10	10	10

QQ Plot

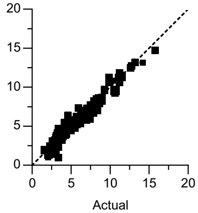

Supplementary Figure 2. Normality tests for Figure 2C datasets.

A), and B) (left panels) Tables of the statistical normality test D'Agostino-Pearson omnibus performed on datasets derived from Figure 2C. (Right panels) QQ normality plots resulting from this normality test. Control diet (CD), ketogenic diet (KD).

A

Test for normal distribution of CD male β-HB datasets

D'Agostino & Pearson Test	CD 1	CD 3	CD 5	CD 8	CD 10	CD 12	CD 15	CD 17	CD 19	CD 22	CD 24	CD 26
K2	0.2	0.7	5.9	3.1	3.0	8.8	1.4	0.2	10.4	2.3	0.6	3.2
P value	0.9	0.7	0.1	0.2	0.2	0.0	0.5	0.9	0.01	0.3	0.8	0.2
Passed normality test (alpha=0.05)?	Yes	Yes	Yes	Yes	Yes	No	Yes	Yes	No	Yes	Yes	Yes
P value summary	ns	ns	ns	ns	ns	*	ns	ns	**	ns	ns	ns
Number of values	10	10	10	10	10	10	10	10	10	10	10	10

QQ Plot

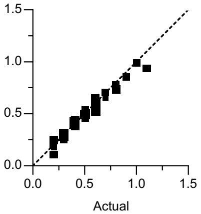

B

Test for normal distribution of KD male β-HB datasets

D'Agostino & Pearson Test	KD 1	KD 3	KD 5	KD 8	KD 10	KD 12	KD 15	KD 17	KD 19	KD 22	KD 24	KD 26
K2	0.6	0.3	1.1	2.2	2.9	5.7	2.5	0.5	1.4	1.8	1.9	5.3
P value	0.8	0.9	0.6	0.3	0.2	0.1	0.3	0.8	0.5	0.4	0.4	0.1
Passed normality test (alpha=0.05)?	Yes	Yes	Yes	Yes	Yes	Yes	Yes	Yes	Yes	Yes	Yes	Yes
P value summary	ns	ns	ns	ns	ns	ns	ns	ns	ns	ns	ns	ns
Number of values	10	10	10	10	10	10	10	10	10	10	10	10

QQ Plot

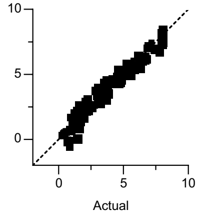

Supplementary Figure 3. Normality tests for Figure 2E datasets.

A), and B) (right panels) Tables of the statistical normality test D'Agostino-Pearson omnibus performed on datasets derived from Figure 2E. (Right panels) QQ normality plots resulting from this normality test. Control diet (CD), ketogenic diet (KD), β-hydroxybutyrate (β-HB).

A

Test for normal distribution of CD male lactate datasets

D'Agostino & Pearson Test	CD 1	CD 3	CD 5	CD 8	CD 10	CD 12	CD 15	CD 17	CD 19	CD 22	CD 24	CD 26
K2	1.2	1.7	6.5	1.6	3.0	2.6	3.7	0.7	4.8	0.8	5.1	12.9
P value	0.5	0.4	0.04	0.5	0.2	0.3	0.2	0.7	0.1	0.7	0.1	0.002
Passed normality test (alpha=0.05)?	Yes	Yes	No	Yes	Yes	Yes	Yes	Yes	Yes	Yes	Yes	No
P value summary	ns	ns	*	ns	ns	ns	ns	ns	ns	ns	ns	**
Number of values	10	10	10	10	10	10	10	10	10	10	10	10

QQ Plot

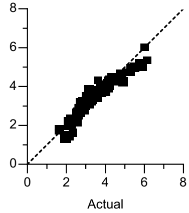

B

Test for normal distribution of KD male lactate datasets

D'Agostino & Pearson Test	KD 1	KD 3	KD 5	KD 8	KD 10	KD 12	KD 15	KD 17	KD 19	KD 22	KD 24	KD 26
K2	9.1	0.1	1.0	1.0	2.8	11.1	2.5	13.7	18.0	0.3	1.4	3.6
P value	0.01	0.9	0.6	0.6	0.3	0.004	0.3	0.001	<0.001	0.9	0.5	0.2
Passed normality test (alpha=0.05)?	No	Yes	Yes	Yes	Yes	No	Yes	No	No	Yes	Yes	Yes
P value summary	*	ns	ns	ns	ns	**	ns	**	***	ns	ns	ns
Number of values	10	10	10	10	10	10	10	10	10	10	10	10

QQ Plot

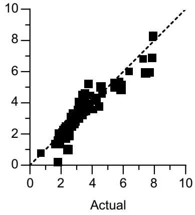

Supplementary Figure 4. Normality tests for Figure 2G datasets.

A), and B) (right panels) Tables of the statistical normality test D'Agostino-Pearson omnibus performed on datasets derived from Figure 2G. (Right panels) QQ normality plots resulting from this normality test. Control diet (CD), ketogenic diet (KD).

A

Test for normal distribution of CD male BW datasets

D'Agostino & Pearson Test	CD 1	CD 3	CD 5	CD 8	CD 10	CD 12	CD 15	CD 17	CD 19	CD 22	CD 24	CD 26
K2	1.3	3.5	1.0	3.4	0.8	1.7	2.0	1.0	1.4	0.9	0.9	0.7
P value	0.5	0.2	0.6	0.2	0.7	0.4	0.4	0.6	0.5	0.6	0.6	0.7
Passed normality test (alpha=0.05)?	Yes	Yes	Yes	Yes	Yes	Yes	Yes	Yes	Yes	Yes	Yes	Yes
P value summary	ns	ns	ns	ns	ns	ns	ns	ns	ns	ns	ns	ns
Number of values	10	10	10	10	10	10	10	10	10	10	10	10

QQ Plot

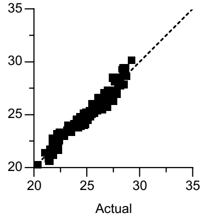

B

Test for normal distribution of KD male BW datasets

D'Agostino & Pearson Test	KD 1	KD 3	KD 5	KD 8	KD 10	KD 12	KD 15	KD 17	KD 19	KD 22	KD 24	KD 26
K2	1.0	2.3	0.3	0.3	0.2	0.0	0.7	1.1	0.6	2.7	3.3	4.6
P value	0.6	0.3	0.9	0.9	0.9	1.0	0.7	0.6	0.7	0.3	0.2	0.1
Passed normality test (alpha=0.05)?	Yes	Yes	Yes	Yes	Yes	Yes	Yes	Yes	Yes	Yes	Yes	Yes
P value summary	ns	ns	ns	ns	ns	ns	ns	ns	ns	ns	ns	ns
Number of values	10	10	10	10	10	10	10	10	10	10	10	10

QQ Plot

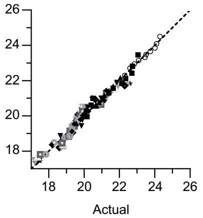

Supplementary Figure 5. Normality tests for Figure 3A male datasets.

A), and B) (left panels) Tables of the statistical normality test D'Agostino-Pearson omnibus performed on male datasets derived from Figure 3A. (Right panels) QQ normality plots resulting from this normality test. Control diet (CD), ketogenic diet (KD), body weight (BW).

A

Test for normal distribution of CD female BW datasets

D'Agostino & Pearson Test	CD 1	CD 3	CD 5	CD 8	CD 10	CD 12	CD 15	CD 17	CD 19	CD 22	CD 24	CD 26
K2	1.1	2.0	2.9	2.1	1.6	3.9	5.3	2.4	4.2	1.0	2.4	1.1
P value	0.6	0.4	0.2	0.4	0.4	0.1	0.1	0.3	0.1	0.6	0.3	0.6
Passed normality test (alpha=0.05)?	Yes	Yes	Yes	Yes	Yes	Yes	Yes	Yes	Yes	Yes	Yes	Yes
P value summary	ns	ns	ns	ns	ns	ns	ns	ns	ns	ns	ns	ns
Number of values	10	10	10	10	10	10	10	10	10	10	10	10

QQ Plot

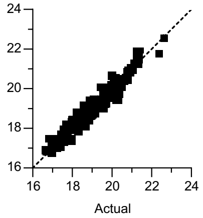

B

Test for normal distribution of KD female BW datasets

D'Agostino & Pearson Test	KD 1	KD 3	KD 5	KD 8	KD 10	KD 12	KD 15	KD 17	KD 19	KD 22	KD 24	KD 26
K2	3.4	3.2	1.6	1.6	2.6	3.3	1.4	1.2	0.5	0.1	0.2	0.4
P value	0.2	0.2	0.5	0.5	0.3	0.2	0.5	0.6	0.8	1.0	0.9	0.8
Passed normality test (alpha=0.05)?	Yes	Yes	Yes	Yes	Yes	Yes	Yes	Yes	Yes	Yes	Yes	Yes
P value summary	ns	ns	ns	ns	ns	ns	ns	ns	ns	ns	ns	ns
Number of values	10	10	10	10	10	10	10	10	10	10	10	10

QQ Plot

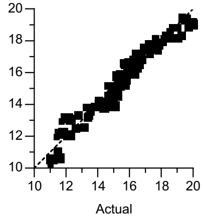

Supplementary Figure 6. Normality tests for Figure 3A female datasets.

A), and B) (left panels) Tables of the statistical normality test D'Agostino-Pearson omnibus performed on female datasets derived from Figure 3A. (Right panels) QQ normality plots resulting from this normality test. Control diet (CD), ketogenic diet (KD), body weight (BW).

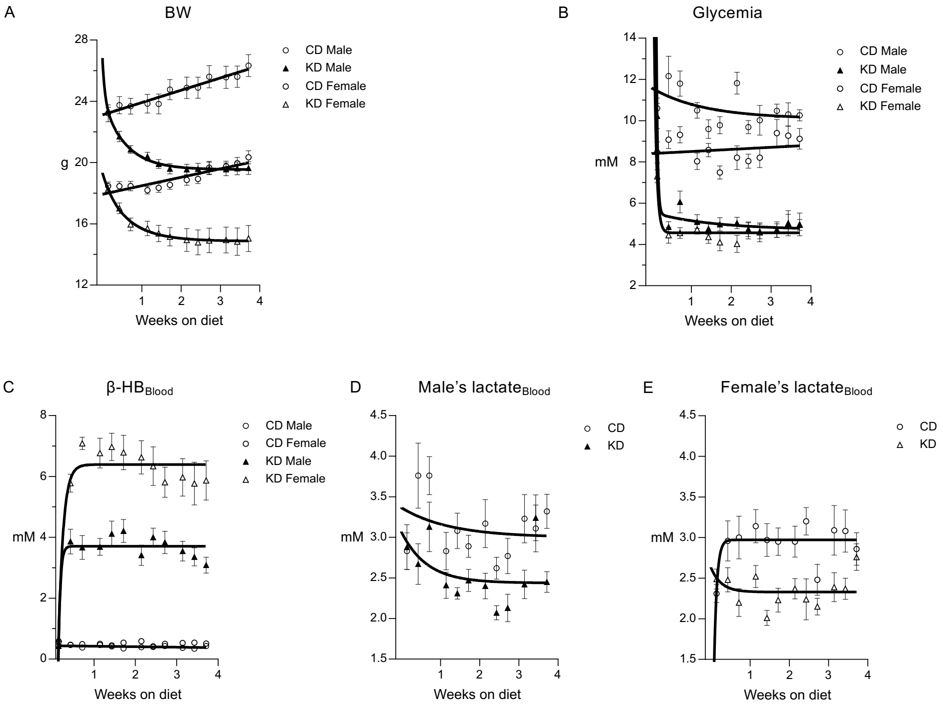

Supplementary Figure 7. Nonlinear regression analysis of metabolic ketosis biomarkers.

Two-phase models (black lines) were applied to assess kinetic changes over weeks in various metabolic parameters in male (black symbols) and female (white symbols) mice fed Bio-Serv's control diet (CD, circle) or ketogenic diet (KD, triangle). Two-phase decay models were used to analyze the body weight (BW, g, A), glycemia (mM, B), and blood lactate of males (mM, D), and females (mM, E). Two-phase association models were performed for the blood concentration of β-HB (mM, C). Simple linear regression analysis was applied to β-hydroxybutyrate (β-HB) CD data (mM, C). N = 10 mice per Bio-Serv's dietary group. Data are presented as Mean ± SEM.

A

Test for normal distribution of CD male glycemia datasets

D'Agostino & Pearson Test	CD 1	CD 3	CD 5	CD 8	CD 10	CD 12	CD 15	CD 17	CD 19	CD 22	CD 24	CD 26
K2	0.5	2.3	3.5	0.6	0.7	1.6	1.9	1.8	22.3	0.6	1.2	0.5
P value	0.8	0.3	0.2	0.8	0.7	0.4	0.4	0.4	<0.001	0.8	0.6	0.8
Passed normality test (alpha=0.05)?	Yes	Yes	Yes	Yes	Yes	Yes	Yes	Yes	No	Yes	Yes	Yes
P value summary	ns	ns	ns	ns	ns	ns	ns	ns	***	ns	ns	ns
Number of values	10	10	10	10	10	10	10	10	10	10	10	10

QQ Plot

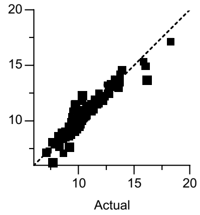

B

Test for normal distribution of KD male glycemia datasets

D'Agostino & Pearson Test	KD 1	KD 3	KD 5	KD 8	KD 10	KD 12	KD 15	KD 17	KD 19	KD 22	KD 24	KD 26
K2	6.7	0.7	12.4	1.0	1.0	0.5	2.4	3.7	3.1	0.7	1.6	3.1
P value	0.0	0.7	0.0	0.6	0.6	0.8	0.3	0.2	0.2	0.7	0.4	0.2
Passed normality test (alpha=0.05)?	No	Yes	No	Yes	Yes	Yes	Yes	Yes	Yes	Yes	Yes	Yes
P value summary	*	ns	**	ns	ns	ns	ns	ns	ns	ns	ns	ns
Number of values	10	10	10	10	10	10	10	10	10	10	10	10

QQ Plot

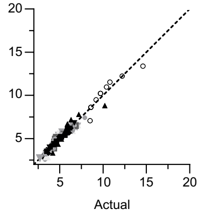

Supplementary Figure 8. Normality tests for Figure 4A male datasets.

A), and B) (left panels) Tables of the statistical normality test D'Agostino-Pearson omnibus performed on male datasets derived from Figure 4A. (Right panels) QQ normality plots resulting from this normality test. Control diet (CD), ketogenic diet (KD).

A

Test for normal distribution of CD female glycemia datasets

D'Agostino & Pearson Test	CD 1	CD 3	CD 5	CD 8	CD 10	CD 12	CD 15	CD 17	CD 19	CD 22	CD 24	CD 26
K2	2.4	1.2	0.2	1.6	19.1	2.7	2.7	3.7	4.5	0.2	0.9	8.8
P value	0.3	0.5	0.9	0.4	<0.001	0.3	0.3	0.2	0.1	0.9	0.6	0.01
Passed normality test (alpha=0.05)?	Yes	Yes	Yes	Yes	No	Yes	Yes	Yes	Yes	Yes	Yes	No
P value summary	ns	ns	ns	ns	***	ns	ns	ns	ns	ns	ns	*
Number of values	10	10	10	10	10	10	10	10	10	10	10	10

QQ Plot

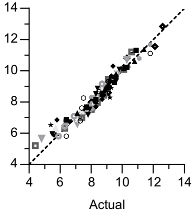

B

Test for normal distribution of KD female glycemia datasets

D'Agostino & Pearson Test	KD 1	KD 3	KD 5	KD 8	KD 10	KD 12	KD 15	KD 17	KD 19	KD 22	KD 24	KD 26
K2	0.2	3.2	1.5	7.2	2.3	19.8	15.3	0.3	0.5	0.2	1.7	1.0
P value	0.9	0.2	0.5	0.0	0.3	<0.001	<0.001	0.9	0.8	0.9	0.4	0.6
Passed normality test (alpha=0.05)?	Yes	Yes	Yes	No	Yes	No	No	Yes	Yes	Yes	Yes	Yes
P value summary	ns	ns	ns	*	ns	***	***	ns	ns	ns	ns	ns
Number of values	10	10	10	10	10	10	10	10	10	10	10	10

QQ Plot

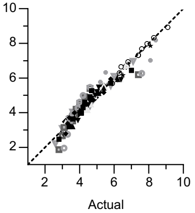

Supplementary Figure 9. Normality tests for Figure 4A female datasets.

A), and B) (left panels) Tables of the statistical normality test D'Agostino-Pearson omnibus performed on female datasets derived from Figure 4A. (Right panels) QQ normality plots resulting from this normality test. Control diet (CD), ketogenic diet (KD).

A

Test for normal distribution of CD male blood β-HB datasets

D'Agostino & Pearson Test	CD 1	CD 3	CD 5	CD 8	CD 10	CD 12	CD 15	CD 17	CD 19	CD 22	CD 24	CD 26
K2	13.9	12.6	1.1	3.9	4.9	6.8	16.2	1.2	1.0	1.5	0.2	7.3
P value	<0.001	0.0	0.6	0.1	0.1	0.0	<0.001	0.6	0.6	0.5	0.9	0.0
Passed normality test (alpha=0.05)?	No	No	Yes	Yes	Yes	No	No	Yes	Yes	Yes	Yes	No
P value summary	***	**	ns	ns	ns	*	***	ns	ns	ns	ns	*
Number of values	10	10	10	10	10	10	10	10	10	10	10	10

QQ Plot

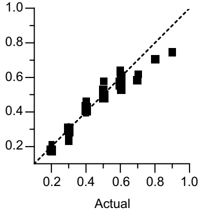

B

Test for normal distribution of KD male blood β-HB datasets

D'Agostino & Pearson Test	KD 1	KD 3	KD 5	KD 8	KD 10	KD 12	KD 15	KD 17	KD 19	KD 22	KD 24	KD 26
K2	8.2	1.9	0.4	0.0	0.9	1.5	5.2	6.9	9.1	0.2	0.2	1.2
P value	0.0	0.4	0.8	>0.99	0.6	0.5	0.1	0.0	0.0	0.9	0.9	0.5
Passed normality test (alpha=0.05)?	No	Yes	Yes	Yes	Yes	Yes	Yes	No	No	Yes	Yes	Yes
P value summary	*	ns	ns	ns	ns	ns	ns	*	*	ns	ns	ns
Number of values	10	10	10	10	10	10	10	10	10	10	10	10

QQ Plot

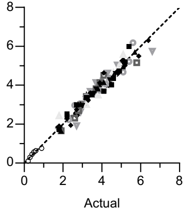

Supplementary Figure 10. Normality tests for Figure 5A male datasets.

A), and B) (left panels) Tables of the statistical normality test D'Agostino-Pearson omnibus performed on male datasets derived from Figure 5A. (Right panels) QQ normality plots resulting from this normality test. Control diet (CD), ketogenic diet (KD), β-hydroxybutyrate (β-HB).

A

Test for normal distribution of CD female blood β-HB datasets

D'Agostino & Pearson Test	CD 1	CD 3	CD 5	CD 8	CD 10	CD 12	CD 15	CD 17	CD 19	CD 22	CD 24	CD 26
K2	1.2	1.5	1.1	0.9	0.2	1.5	0.9	1.0	0.5	1.0	1.5	1.1
P value	0.6	0.5	0.6	0.6	0.9	0.5	0.6	0.6	0.8	0.6	0.5	0.6
Passed normality test (alpha=0.05)?	Yes	Yes	Yes	Yes	Yes	Yes	Yes	Yes	Yes	Yes	Yes	Yes
P value summary	ns	ns	ns	ns	ns	ns	ns	ns	ns	ns	ns	ns
Number of values	10	10	10	10	10	10	10	10	10	10	10	10

QQ Plot

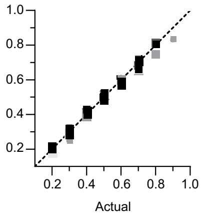

B

Test for normal distribution of KD female blood β-HB datasets

D'Agostino & Pearson Test	KD 1	KD 3	KD 5	KD 8	KD 10	KD 12	KD 15	KD 17	KD 19	KD 22	KD 24	KD 26
K2	0.15	1.45	2.54	11.39	16.25	8.69	7.76	3.14	0.54	2.50	1.44	1.52
P value	0.93	0.48	0.28	0.003	<0.001	0.01	0.02	0.21	0.76	0.29	0.49	0.47
Passed normality test (alpha=0.05)?	Yes	Yes	Yes	No	No	No	No	Yes	Yes	Yes	Yes	Yes
P value summary	ns	ns	ns	**	***	*	*	ns	ns	ns	ns	ns
Number of values	10	10	10	10	10	10	10	10	10	10	10	10

QQ Plot

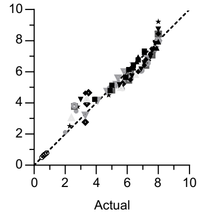

Supplementary Figure 11. Normality tests for Figure 5A female datasets.

A), and B) (left panels) Tables of the statistical normality test D'Agostino-Pearson omnibus performed on female datasets derived from Figure 5A. (Right panels) QQ normality plots resulting from this normality test. Control diet (CD), ketogenic diet (KD), β-hydroxybutyrate (β-HB).

A

Test for normal distribution of CD male blood lactate datasets

D'Agostino & Pearson Test	CD 1	CD 3	CD 5	CD 8	CD 10	CD 12	CD 15	CD 17	CD 19	CD 22	CD 24	CD 26
K2	12.2	1.8	0.6	2.4	1.0	0.4	2.7	0.2	4.9	0.3	2.7	0.7
P value	0.002	0.4	0.7	0.3	0.6	0.8	0.3	0.9	0.1	0.9	0.3	0.7
Passed normality test (alpha=0.05)?	No	Yes	Yes	Yes	Yes	Yes	Yes	Yes	Yes	Yes	Yes	Yes
P value summary	**	ns	ns	ns	ns	ns	ns	ns	ns	ns	ns	ns
Number of values	10	10	10	10	10	10	10	10	10	10	10	10

QQ Plot

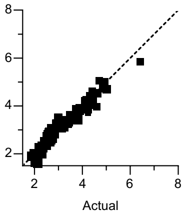

B

Test for normal distribution of KD male blood lactate datasets

D'Agostino & Pearson Test	KD 1	KD 3	KD 5	KD 8	KD 10	KD 12	KD 15	KD 17	KD 19	KD 22	KD 24	KD 26
K2	7.6	1.3	0.9	0.4	2.4	9.1	2.8	6.6	0.9	1.4	7.0	0.1
P value	0.02	0.5	0.7	0.8	0.3	0.0	0,2	0.0	0.7	0.5	0.0	1.0
Passed normality test (alpha=0.05)?	No	Yes	Yes	Yes	Yes	No	Yes	No	Yes	Yes	No	Yes
P value summary	*	ns	ns	ns	ns	*	ns	*	ns	ns	*	ns
Number of values	10	10	10	10	10	10	10	10	10	10	10	10

QQ Plot

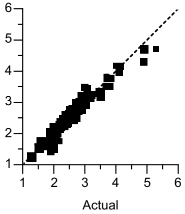

Supplementary Figure 12. Normality tests for Figure 6A male datasets.

A), and B) (left panels) Tables of the statistical normality test D'Agostino-Pearson omnibus performed on male datasets derived from Figure 6A. (Right panels) QQ normality plots resulting from this normality test. Control diet (CD), ketogenic diet (KD).

A

Test for normal distribution of CD female blood lactate datasets

D'Agostino & Pearson Test	CD 1	CD 3	CD 5	CD 8	CD 10	CD 12	CD 15	CD 17	CD 19	CD 22	CD 24	CD 26
K2	1.0	7.7	1.4	1.1	2.7	1.7	9.6	0.6	4.1	9.6	7.5	3.3
P value	0.6	0.02	0.5	0.6	0.3	0.4	0.01	0.8	0.1	0.01	0.02	0.2
Passed normality test (alpha=0.05)?	Yes	No	Yes	Yes	Yes	Yes	No	Yes	Yes	No	No	Yes
P value summary	ns	*	ns	ns	ns	ns	**	ns	ns	**	*	ns
Number of values	10	10	10	10	10	10	10	10	10	10	10	10

QQ Plot

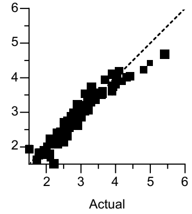

B

Test for normal distribution of KD female blood lactate datasets

D'Agostino & Pearson Test	KD 1	KD 3	KD 5	KD 8	KD 10	KD 12	KD 15	KD 17	KD 19	KD 22	KD 24	KD 26
K2	0.9	8.6	4.1	7.2	0.5	0.5	0.3	4.1	1.2	0.6	6.0	0.6
P value	0.6	0.01	0.1	0.03	0.8	0.8	0.9	0.1	0.6	0.7	0.1	0.7
Passed normality test (alpha=0.05)?	Yes	No	Yes	No	Yes	Yes	Yes	Yes	Yes	Yes	No	Yes
P value summary	ns	*	ns	*	ns	ns	ns	ns	ns	ns	*	ns
Number of values	10	10	10	10	10	10	10	10	10	10	10	10

QQ Plot

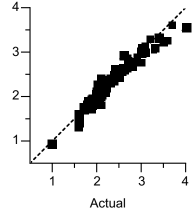

Supplementary Figure 13. Normality tests for Figure 6B female datasets.

A), and B) (right panels) Tables of the statistical normality test D'Agostino-Pearson omnibus performed on female datasets derived from Figure 6B. (Right panels) QQ normality plots resulting from this normality test. Control diet (CD), ketogenic diet (KD).

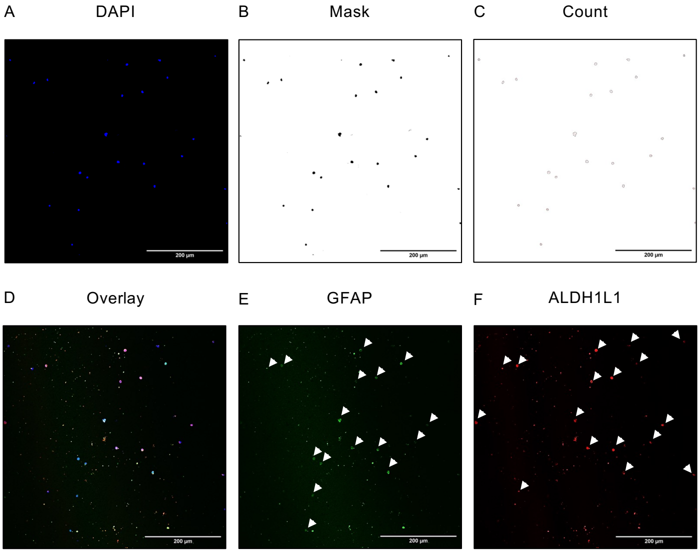

Supplementary Figure 14. Assessment of astrocyte extract purity by immunostaining.

Immunostaining example in a region of interest (ROI) from an astrocyte fraction. A) Nucleus staining with 4′,6-diamidino-2-phenylindole (DAPI, blue). B) DAPI signals were filtered with a mask (black dots) to remove noise. C) The number of nuclei (circles) was automatically counted from the segmented image (B). D) Overlay image showing DAPI (blue), Glial Fibrillary Acidic Protein (GFAP, green), and Aldehyde Dehydrogenase 1 Family Member L1 (ALDH1L1, red) staining within the ROI. E) Split image from D highlighting GFAP staining (green) designated by white arrowheads. F) Split image from D highlighting ALDH1L1 staining (red) designated by white arrowheads. Scale bar = 200 µm.

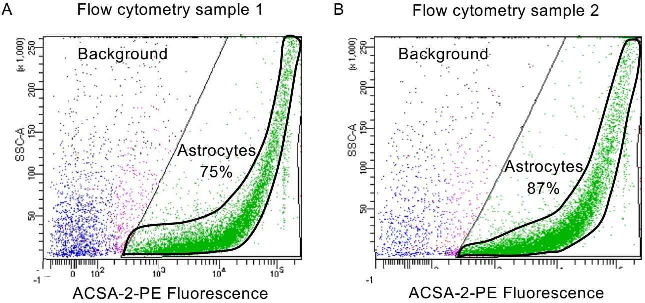

Supplementary Figure 15. Assessment of astrocyte extract purity by flow cytometry analysis

Flow cytometry gating strategy for astrocyte purity validation. Samples 1 (A) and 2 (B) were analyzed after Astrocyte Cell Surface Antigen-2 (ACSA2)-magnetic separation, distinguishing the astrocyte population (green) from the background noise (blue).

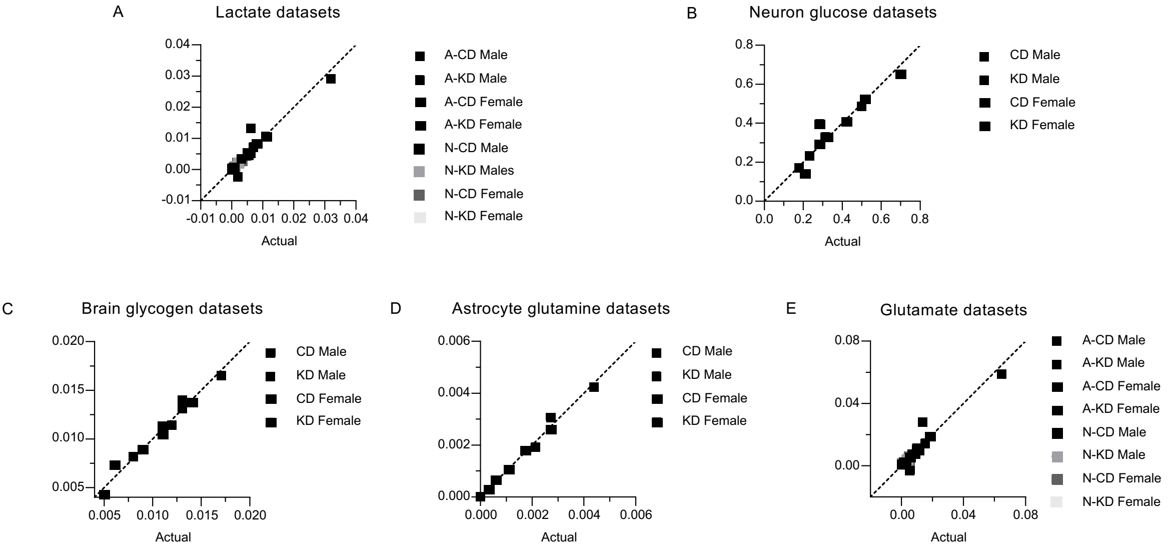

Supplementary Figure 16. QQ normality plots of brain metabolites.

QQ normality plots for brain metabolites, including A) lactate, B) glucose, C) glycogen, D) glutamine, and E) glutamate. Metabolite levels were measured in astrocytes (A), neurons (N), or brain tissues from mice fed either control or ketogenic diet (CD or KD).

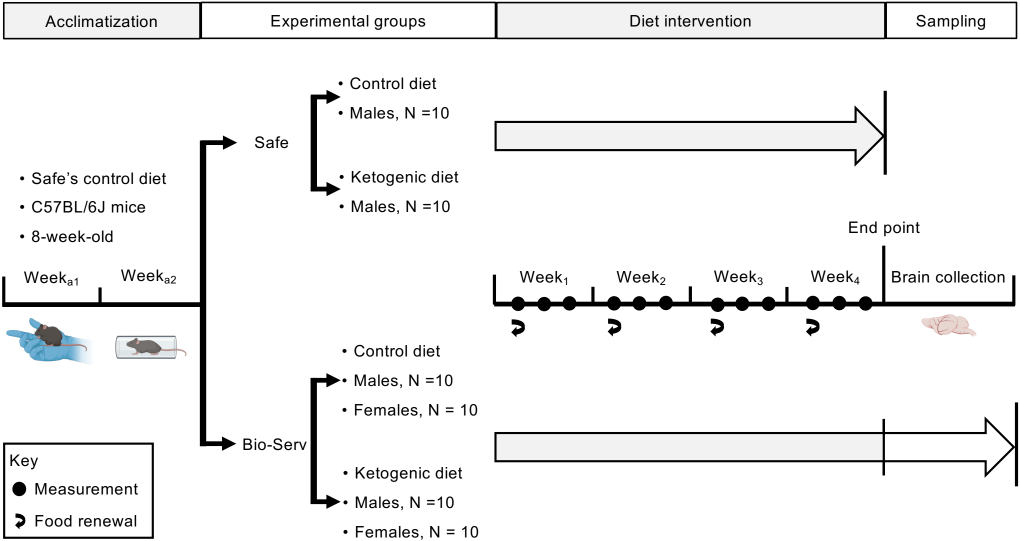

Supplementary Figure 17. Study design scheme.

Stepwise workflow illustrating the two-week acclimatization period (Week$_{a1}$ and Week$_{a2}$), during which mice of 8 weeks old were habituated to gentle handling and restraining while being fed Safe's control diet (CD). Then, mice were randomly assigned to one of four dietary groups for four weeks, including Safe's CD, Safe's ketogenic diet (KD), Bio-Serv's CD, or Bio-Serv's KD. Throughout the diet intervention and until its end point, each individual was weighed and monitored for glycemia, blood β-hydroxybutyrate (β-HB), and blood lactate levels three times a week (black circles). The food was replaced once a week (rounded arrows). After one month, only the brains of Bio-Serv's groups were collected to perform the subsequent analyses. The illustrations were created with BioRender.com. Week$_{a1}$ (week acclimatization 1), week$_{a2}$ (week acclimatization 2).

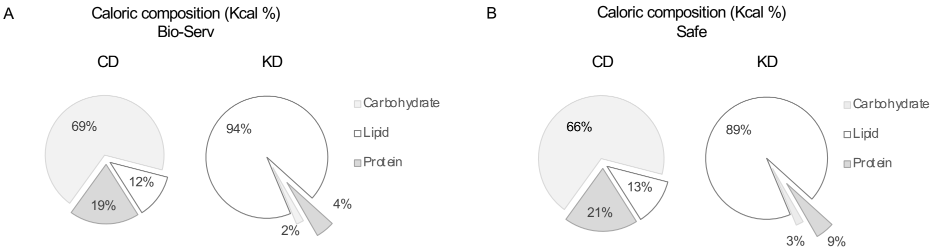

C

Nutritional profile - Bio-Serv

			CD	KD
**Proteins**		Kcal/g	0.7	0.3
		g %	18.1	8.6
**Lipids**		Kcal/g	0.5	6.8
		g %	5.1	75.1
**Carbohydrates**		Kcal/g	2.6	0.1
		g %	65.2	3.2
**Fatty Acids**	**Saturated**	g/Kg	6.5	303
	**Monounsaturated**	g/Kg	13.8	288
	**Polyunsaturated**	g/Kg	27.3	122
**Total**		Kcal/g	3.8	7.2

D

Nutritional profile - Safe

			CD	KD
**Proteins**		Kcal/g	0.7	0.6
		g %	18	15.1
**Lipids**		Kcal/g	0.4	6.1
		g %	4.8	68.2
**Carbohydrates**		Kcal/g	2.3	0.2
		g %	56.9	4.6
**Fatty Acids**	**Saturated**	g/Kg	5.9	3
	**Monounsaturated**	g/Kg	19	211
	**Polyunsaturated**	g/Kg	15.5	96
**Total**		Kcal/g	3.4	6.9

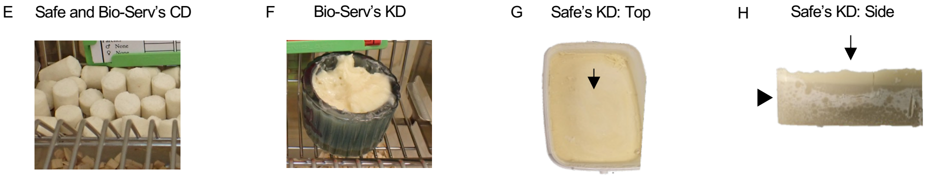

Supplementary Figure 18. Dietary composition and form.

Caloric composition in carbohydrates (light gray), lipids (white), and proteins (dark gray) (kilocalories %, Kcal %) of the four diets used in this study, including Bio-Serv's control and ketogenic diets (CD and KD, A), as well as Safe's CD and KD (B). Nutritional profiles of each diet Bio-Serv (C) and Safe (D), detailing macronutrient (proteins, lipids, and carbohydrates) content in kilocalories per gram of food (Kcal/ g), and as a proportion by weight (g %), and for fatty acids in gram per kilograms (g/Kg) of food. Pictures showing the different forms of the diets: (E) Safe's and Bio-Serv's CD as pellets placed in the tray of mouse housing cages; (F) Bio-Serv's KD as a smooth, creamy fat paste provided in glass feeders; (G) top view and (H) side view of Safe's KD highlighting the distinct yellow (black arrows) and brown (black arrowhead) layers.

Supplementary Table 1: Statistical analysis of body weight datasets from Safe's diet

		Body weight / Mouse (g)																			
		CD Male										KD Male									
	Cohort	A			B			C				A			B			C			
	Mouse n°	1	2	3	1	2	3	1	2	3	4	1	2	3	1	2	3	1	2	3	4
Day	1	24.4	24.7	21.9	25.0	21.9	24.8	26.2	20.1	23.4	23.2	25.7	21.8	26.2	25.0	24.1	24.0	23.1	23.4	22.8	23.5
	3	25.0	24.2	22.0	25.0	21.8	24.7	25.2	20.4	23.4	23.3	22.0	18.5	22.2	22.0	19.6	20.6	22.1	22.4	21.9	23.0
	5	25.4	26.2	22.2	25.3	22.9	25.5	27.0	21.3	24.0	24.0	20.8	17.3	20.6	20.2	17.8	18.7	20.7	20.5	21.4	21.1
	8	25.4	26.1	22.2	26.0	23.3	26.1	27.3	22.4	24.2	24.1	18.7	15.5	18.9	21.4	19.8	17.2	20.8	20.6	20.9	20.1
	10	25.6	26.5	22.2	26.2	23.7	27.0	27.7	22.8	24.5	24.8	18.7	14.8	18.6	21.9	20.7	16.2	19.9	19.8	19.7	19.2
	12	25.9	27.3	22.7	26.9	24.7	27.8	28.4	23.3	24.8	25.3	20.0	14.7	18.7	21.8	20.8	15.6	20.3	20.5	20.4	19.8
	15	26.7	27.4	23.6	26.8	25.4	28.7	28.7	24.1	25.3	25.8	20.8	14.4	19.1	23.4	21.6	16.8	19.9	20.7	19.4	20.1
	17	26.7	27.3	24.3	27.6	25.8	28.7	29.0	24.5	25.1	26.0	21.9	15.0	19.5	23.5	21.0	17.7	20.5	21.0	20.2	20.9
	19	27.9	27.6	24.7	27.5	26.0	29.1	29.1	24.6	25.5	25.8	23.0	16.7	22.2	23.9	21.1	18.9	22.6	22.4	22.0	22.4
	22	27.3	27.6	24.6	28.2	26.8	29.4	30.0	25.7	25.1	26.6	22.7	17.4	22.3	23.4	20.1	18.7	23.5	23.5	23.4	23.4
	24	27.8	26.9	24.7	28.0	26.5	29.0	29.7	25.5	25.2	26.1	22.8	17.3	22.8	23.8	20.1	18.5	23.9	23.9	24.2	24.1
	26	28.4	28.0	25.7	28.4	26.7	29.2	30.7	26.2	26.0	27.3	23.0	17.3	23.0	25.3	22.0	21.1	24.6	23.8	24.3	24.4

```
Univariate Type III Repeated-Measures ANOVA Assuming Sphericity

Sum Sq num Df Error SS den Df  F value    Pr(>F)
(Intercept)    130312      1   670.32     18 3499.258 < 2.2e-16 ***
Treatment        1363      1   670.32     18   36.608 1.017e-05 ***
Time              275     11   128.71    198   38.524 < 2.2e-16 ***
Treatment:Time    229     11   128.71    198   31.956 < 2.2e-16 ***
---
Signif. codes:  0 '***' 0.001 '**' 0.01 '*' 0.05 '.' 0.1 ' ' 1


Mauchly Tests for Sphericity

Test statistic    p-value
Time              1.7187e-08 5.4041e-23
Treatment:Time    1.7187e-08 5.4041e-23


Greenhouse-Geisser and Huynh-Feldt Corrections
for Departure from Sphericity

GG eps Pr(>F[GG])
Time           0.27483  1.546e-13 ***
Treatment:Time 0.27483  4.321e-12 ***
---
Signif. codes:  0 '***' 0.001 '**' 0.01 '*' 0.05 '.' 0.1 ' ' 1

HF eps   Pr(>F[HF])
Time           0.336518 4.332764e-16
Treatment:Time 0.336518 2.521418e-14
```

Normal distribution: D'Agostino & Pearson test

**Control Diet datasets**	CD 1	CD 3	CD 5	CD 8	CD 10	CD 12	CD 15	CD 17	CD 19	CD 22	CD 24	CD 26
K2	0.8	1.4	0.7	1.0	0.9	0.7	0.3	0.9	1.7	0.3	0.8	0.7
P value	0.7	0.5	0.7	0.6	0.7	0.7	0.8	0.6	0.4	0.9	0.7	0.7
Passed normality test (alpha=0.05)?	Yes	Yes	Yes	Yes	Yes	Yes	Yes	Yes	Yes	Yes	Yes	Yes
P value summary	ns	ns	ns	ns	ns	ns	ns	ns	ns	ns	ns	ns
Number of values	10	10	10	10	10	10	10	10	10	10	10	10

**Ketogenic Diet datasets**	KD 1	KD 3	KD 5	KD 8	KD 10	KD 12	KD 15	KD 17	KD 19	KD 22	KD 24	KD 26
K2	0.2	4.2	2.4	3.3	2.4	4.4	2.8	4.5	7.1	3.2	2.9	9.4
P value	0.9	0.1	0.3	0.2	0.3	0.1	0.2	0.1	0.03	0.2	0.2	0.01
Passed normality test (alpha=0.05)?	Yes	Yes	Yes	Yes	Yes	Yes	Yes	Yes	No	Yes	Yes	No
P value summary	ns	ns	ns	ns	ns	ns	ns	ns	*	ns	ns	**
Number of values	10	10	10	10	10	10	10	10	10	10	10	10

**Type of Test**	Unpaired T-test	Unpaired T-test	Unpaired T-test	Unpaired T-test	Unpaired T-test	Unpaired T-test	Unpaired T-test	Unpaired T-test	Mann-Whitney T-test	Unpaired T-test	Unpaired T-test	Mann-Whitney T-test
Data organization	CD 1 vs KD 1	CD 3 vs KD 3	CD 5 vs KD 5	CD 8 vs KD 8	CD 10 vs KD 10	CD 12 vs KD 12	CD 15 vs KD 15	CD 17 vs KD 17	CD 19 vs KD 19	CD 22 vs KD 22	CD 24 vs KD 24	CD 26 vs KD 26
Number of values	10 vs 10	10 vs 10	10 vs 10	10 vs 10	10 vs 10	10 vs 10	10 vs 10	10 vs 10	10 vs 10	10 vs 10	10 vs 10	10 vs 10
P value	0.58	0.007	<0.001	<0.001	<0.001	<0.001	<0.001	<0.001	<0.001	<0.001	<0.001	<0.001
P value summary	ns	**	***	***	***	***	***	***	***	***	***	***
Significantly different (P < 0.05)?	No	Yes	Yes	Yes	Yes	Yes	Yes	Yes	Yes	Yes	Yes	Yes
One- or two-tailed P value?	Two-tailed	Two-tailed	Two-tailed	Two-tailed	Two-tailed	Two-tailed	Two-tailed	Two-tailed	Two-tailed	Two-tailed	Two-tailed	Two-tailed
t, df or *Sum of ranks in column I,U*	t=0.5584, df=18	t=3.043, df=18	t=6.048, df=18	t=6.693, df=18	t=7.031, df=18	t=6.828, df=18	t=6.888, df=18	t=7.011, df=18	*155 , 55*	t=5.791, df=18	t=4.981, df=18	*155 , 55*

**How big is the difference?**												
Mean of column A or *Median of column I*	23.54	23.49	24.36	24.7	25.09	25.7	26.24	26.48	*26.73, n=10*	27.13	26.93	*27.65, n=10*
Mean of column M or *Median of column U*	23.94	21.41	19.89	19.35	18.94	19.24	19.61	20.12	*22.30, n=10*	21.84	22.13	*23.40, n=10*
Difference between means (M - A) ± SEM or *Difference: Actual*	0.4000 ± 0.7163	-2.079 ± 0.6833	-4.470 ± 0.7391	-5.352 ± 0.7997	-6.153 ± 0.8751	-6.467 ± 0.9472	-6.629 ± 0.9624	-6.365 ± 0.9078	*-4.425*	-5.289 ± 0.9133	-4.799 ± 0.9635	*-4.25*
95% confidence interval or *Difference: Hodges-Lehmann*	-1.105 to 1.905	-3.515 to -0.6435	-6.023 to -2.917	-7.032 to -3.672	-7.992 to -4.314	-8.457 to -4.477	-8.651 to -4.607	-8.272 to -4.458	*-5.08*	-7.208 to -3.370	-6.823 to -2.775	*-4.27*
R squared (eta squared)	0.01703	0.3396	0.6702	0.7133	0.7331	0.7214	0.725	0.732		0.6507	0.5795	

**F test to compare variances**												
F, DFn, Dfd	1.761, 9, 9	1.330, 9, 9	1.626, 9, 9	1.142, 9, 9	1.286, 9, 9	1.488, 9, 9	2.098, 9, 9	2.015, 9, 9	1.659, 9, 9	1.678, 9, 9	2.337, 9, 9	2.179, 9, 9
P value	0.41	0.68	0.48	0.85	0.71	0.56	0.28	0.31	0.46	0.45	0.22	0.26
P value summary	ns	ns	ns	ns	ns	ns	ns	ns	ns	ns	ns	ns
Significantly different (P < 0.05)?	No	No	No	No	No	No	No	No	No	No	No	No

**Descriptive statistics**	CD Male			KD Male		
Days	Mean	SEM	N	Mean	SEM	N
1	23.5	0.6	10	23.9	0.4	10
3	23.5	0.5	10	21.4	0.4	10
5	24.4	0.6	10	19.9	0.5	10
8	24.7	0.5	10	19.3	0.6	10
10	25.1	0.6	10	18.9	0.7	10
12	25.7	0.6	10	19.2	0.7	10
15	26.2	0.5	10	19.6	0.8	10
17	26.5	0.5	10	20.1	0.7	10
19	26.7	0.5	10	21.5	0.7	10
22	27.1	0.6	10	21.8	0.7	10
24	26.9	0.5	10	22.1	0.8	10
26	27.7	0.5	10	22.9	0.7	10

Supplementary Table 2: Statistical analysis of body weight gain between CD and KD male

		Body weight loss (%)			
Cohort	Mouse n°	CD_{12} Male	KD_{12} Male	CD_{26} Male	KD_{26} Male
A	1	6.2	-22.3	16.6	-10.5
	2	10.7	-32.7	13.6	-20.6
	3	3.5	-28.5	17.2	-12.2
B	1	7.7	-13.1	13.7	1.1
	2	12.7	-13.5	21.8	-8.7
	3	12.3	-35.1	18.1	-11.9
C	1	8.6	-12.1	17.2	6.3
	2	15.9	-12.6	30.3	1.7
	3	6.0	-10.3	11.1	6.6
	4	9.1	-15.8	17.7	3.8

Test for normal distribution

**D'Agostino & Pearson test**	CD_{12} Male	KD_{12} Male	CD_{26} Male	KD_{26} Male
K2	0.2	2.3	8.5	1.4
P value	0.9	0.3	0.0	0.5
Passed normality test (alpha=0.05)?	Yes	Yes	No	Yes
P value summary	ns	ns	*	ns

**Kruskal-Wallis test**	
P value	<0.001
Exact or approximate P value?	Approximate
P value summary	***
Do the medians vary signif. (P < 0.05)?	Yes
Number of groups	4
Kruskal-Wallis statistic	33.7

**Dunn's multiple comparisons test**	**Mean rank diff.**	**Significant?**	**Summary**	**Adjusted P Value**
CD12 vs. KD12	18.8	Yes	**	0.0
CD12 vs. CD26	-9.7	No	ns	0.4
CD12 vs. KD26	10.1	No	ns	0.3
KD12 vs. CD26	-28.5	Yes	***	<0.001
KD12 vs. KD26	-8.7	No	ns	0.6
CD26 vs. KD26	19.8	Yes	***	<0.001

**Test details**	**Mean rank 1**	**Mean rank 2**	**Mean rank diff.**	**n1**	**n2**	**Z**
CD12 vs. KD12	25.3	6.5	18.8	10.0	10.0	3.6
CD12 vs. CD26	25.3	35.0	-9.7	10.0	10.0	1.9
CD12 vs. KD26	25.3	15.2	10.1	10.0	10.0	1.9
KD12 vs. CD26	6.5	35.0	-28.5	10.0	10.0	5.5
KD12 vs. KD26	6.5	15.2	-8.7	10.0	10.0	1.7
CD26 vs. KD26	35.0	15.2	19.8	10.0	10.0	3.8

**Descriptive statistics**	CD_{12} Male	KD_{12} Male	CD_{26} Male	KD_{26} Male
Number of values	10	10	10	10
Mean	9.3	-20.0	18.0	-4.4
Std. Deviation	3.7	9.3	5.3	9.5
Std. Error of Mean	1.2	3.0	1.7	3.0

Supplementary Table 3: Statistical analysis of glycemia datasets from Safe's diet

		Glycemia / Mouse (mM)																			
		CD Male										KD Male									
	Cohort	A			B			C				A			B			C			
	Mouse n°	1	2	3	1	2	3	1	2	3	4	1	2	3	1	2	3	1	2	3	4
Day	1	12.6	12.3	14.3	11.3	9.7	9.8	9.7	9.0	11.4	10.9	8.3	9.2	9.9	8.8	11.2	9.2	13.1	12.5	15.7	11.1
	3	16.3	13.8	10.6	13.7	8.1	10.2	12.2	12.6	9.5	13.0	5.9	3.8	3.9	4.6	4.1	3.4	11.2	9.0	10.6	12.7
	5	15.6	13.2	10.2	14.8	8.9	12.7	11.1	14.0	7.9	9.4	3.0	2.1	3.1	2.5	4.2	3.6	5.6	2.7	4.5	2.7
	8	13.0	10.1	11.2	10.5	10.8	12.1	10.4	11.7	9.2	9.0	2.4	2.6	3.1	5.1	4.1	2.5	4.9	4.1	4.5	7.3
	10	16.6	15.0	8.4	9.8	10.3	11.3	9.9	14.1	14.8	18.9	3.2	2.1	3.4	4.6	7.9	2.1	3.1	6.9	7.8	2.9
	12	12.8	14.1	10.9	8.0	11.6	13.3	10.6	11.4	8.1	9.7	3.1	1.6	2.8	4.3	3.6	3.3	3.9	4.9	4.7	4.2
	15	12.7	15.0	15.6	11.3	11.9	12.2	9.9	9.9	13.3	8.3	6.8	4.1	4.9	7.1	7.1	6.2	6.3	3.4	3.4	3.8
	17	11.2	14.5	12.7	15.2	14.8	11.6	11.1	10.7	10.1	10.8	5.7	3.2	4.2	5.3	3.0	3.1	4.8	4.2	5.6	3.0
	19	9.3	8.7	10.8	9.2	7.8	12.9	8.8	10.7	9.8	9.8	6.4	2.8	7.6	5.6	3.7	3.3	11.4	10.6	5.8	8.6
	22	12.2	8.2	9.2	7.9	10.8	11.9	5.9	10.3	13.6	9.5	4.5	3.2	5.2	5.0	4.0	3.3	8.0	6.8	8.3	5.7
	24	15.7	10.9	13.4	10.0	9.9	15.1	10.9	10.8	12.5	12.8	4.7	2.1	9.2	6.4	3.3	4.5	14.1	7.8	10.6	8.5
	26	12.7	10.1	11.7	9.5	9.2	11.3	9.9	10.3	7.7	10.5	4.2	2.3	3.3	8.7	8.9	7.6	9.3	7.5	9.8	7.6

```
Univariate Type III Repeated-Measures ANOVA Assuming Sphericity

Sum Sq num Df Error SS den Df  F value    Pr(>F)
(Intercept)    17510.4      1   340.49     18 925.6816 < 2.2e-16 ***
Treatment       1886.1      1   340.49     18  99.7071 9.135e-09 ***
Time             254.3     11   736.85    198   6.2120 9.287e-09 ***
Treatment:Time   347.8     11   736.85    198   8.4973 3.233e-12 ***
---
Signif. codes:  0 '***' 0.001 '**' 0.01 '*' 0.05 '.' 0.1 ' ' 1


Mauchly Tests for Sphericity

Test statistic p-value
Time                 0.0065891  0.3574
Treatment:Time       0.0065891  0.3574


Greenhouse-Geisser and Huynh-Feldt Corrections
for Departure from Sphericity

GG eps Pr(>F[GG])
Time           0.53093  1.584e-05 ***
Treatment:Time 0.53093  2.065e-07 ***
---
Signif. codes:  0 '***' 0.001 '**' 0.01 '*' 0.05 '.' 0.1 ' ' 1

HF eps   Pr(>F[HF])
Time           0.8146431 1.728805e-07
Treatment:Time 0.8146431 2.515973e-10
```

Normal distribution: D'Agostino & Pearson test

**Control Diet datasets**	CD 1	CD 3	CD 5	CD 8	CD 10	CD 12	CD 15	CD 17	CD 19	CD 22	CD 24	CD 26
K2	1.0	0.0	1.7	0.1	1.0	0.4	0.1	2.5	4.1	0.1	1.2	0.4
P value	0.6	>0.99	0.4	0.9	0.6	0.8	1.0	0.3	0.1	1.0	0.5	0.8
Passed normality test (alpha=0.05)?	Yes	Yes	Yes	Yes	Yes	Yes	Yes	Yes	Yes	Yes	Yes	Yes
P value summary	ns	ns	ns	ns	ns	ns	ns	ns	ns	ns	ns	ns
Number of values	10	10	10	10	10	10	10	10	10	10	10	10

**Ketogenic Diet datasets**	KD 1	KD 3	KD 5	KD 8	KD 10	KD 12	KD 15	KD 17	KD 19	KD 22	KD 24	KD 26
K2	2.4	3.0	2.3	2.7	2.3	1.9	5.1	3.4	0.8	1.0	0.6	1.9
P value	0.3	0.2	0.3	0.3	0.3	0.4	0.1	0.2	0.7	0.6	0.7	0.4
Passed normality test (alpha=0.05)?	Yes	Yes	Yes	Yes	Yes	Yes	Yes	Yes	Yes	Yes	Yes	Yes
P value summary	ns	ns	ns	ns	ns	ns	ns	ns	ns	ns	ns	ns
Number of values	10	10	10	10	10	10	10	10	10	10	10	10

**Type of Test**	Unpaired T-test	Unpaired T-test	Welch's T-test	Unpaired T-test	Unpaired T-test	Welch's T-test	Unpaired T-test	Unpaired T-test	Welch's T-test	Unpaired T-test	Unpaired T-test	Unpaired T-test
Data organization	CD 1 vs KD 1	CD 3 vs KD 3	CD 5 vs KD 5	CD 8 vs KD 8	CD 10 vs KD 10	CD 12 vs KD 12	CD 15 vs KD 15	CD 17 vs KD 17	CD 19 vs KD 19	CD 22 vs KD 22	CD 24 vs KD 24	CD 26 vs KD 26
Number of values	10 vs 10	10 vs 10	10 vs 10	10 vs 10	10 vs 10	10 vs 10	10 vs 10	10 vs 10	10 vs 10	10 vs 10	10 vs 10	10 vs 10
P value	0.83	0.002	<0.001	<0.001	<0.001	<0.001	<0.001	<0.001	0.009	<0.001	0.001	0.002
P value summary	ns	**	***	***	***	***	***	***	**	***	**	**
Significantly different (P < 0.05)?	No	Yes	Yes	Yes	Yes	Yes	Yes	Yes	Yes	Yes	Yes	Yes
One- or two-tailed P value?	Two-tailed	Two-tailed	Two-tailed	Two-tailed	Two-tailed	Two-tailed	Two-tailed	Two-tailed	Two-tailed	Two-tailed	Two-tailed	Two-tailed
t, df	t=0.2226, df=18	t=3.723, df=18	t=9.246, df=11.89	t=10.84, df=18	t=6.497, df=18	t=10.29, df=12.97	t=7.663, df=18	t=11.62, df=18	*t=3.071, df=12.92*	t=4.922, df=18	t=3.827, df=18	*t=3.537, df=18*

**How big is the difference?**												
Mean of column A	11.1	12	11.78	10.8	12.91	11.05	12.01	12.27	9.78	9.95	12.2	10.29
Mean of column M	10.9	6.92	3.4	04.06	4.4	3.64	5.31	4.21	6.58	5.4	7.12	6.92
Difference between means (M - A) ± SEM	-0.2000 ± 0.8984	-5.082 ± 1.365	-8.380 ± 0.9064	-6.740 ± 0.6215	-8.510 ± 1.310	-7.410 ± 0.7204	-6.700 ± 0.8743	-8.060 ± 0.6937	-3.200 ± 1.042	-4.550 ± 0.9244	-5.080 ± 1.327	-3.370 ± 0.9529
95% confidence interval	-2.087 to 1.687	-7.950 to -2.214	-10.36 to -6.403	-8.046 to -5.434	-11.26 to -5.758	-8.967 to -5.853	-8.537 to -4.863	-9.517 to -6.603	-5.452 to -0.9479	-6.492 to -2.608	-7.869 to -2.291	-5.372 to -1.368
R squared (eta squared)	0.002746	0.435	0.8779	0.8673	0.7011	0.8908	0.7654	0.8824	0.4219	0.5737	0.4486	0.41

**F test to compare variances**												
F, DFn, Dfd	2.012, 9, 9	2.194, 9, 9	6.068, 9, 9	1.476, 9, 9	2.281, 9, 9	4.305, 9, 9	2.184, 9, 9	2.985, 9, 9	4.357, 9, 9	1.612, 9, 9	3.171, 9, 9	3.652, 9, 9
P value	0.31	0.26	0.01	0.57	0.24	0.04	0.26	0.12	0.04	0.49	0.1	0.07
P value summary	ns	ns	*	ns	ns	*	ns	ns	*	ns	ns	ns
Significantly different (P < 0.05)?	No	No	Yes	No	No	Yes	No	No	Yes	No	No	No

**Descriptive statistics**	CD Male			KD Male		
Days	Mean	SEM	N	Mean	SEM	N
1	11.1	0.5	10	10.9	0.7	10
3	12.0	0.8	10	6.9	1.1	10
5	11.8	0.8	10	3.4	0.3	10
8	10.8	0.4	10	4.1	0.5	10
10	12.9	1.1	10	4.4	0.7	10
12	11.1	0.6	10	3.6	0.3	10
15	12.0	0.7	10	5.3	0.5	10
17	12.3	0.6	10	4.2	0.3	10
19	9.8	0.5	10	6.6	0.9	10
22	10.0	0.7	10	5.4	0.6	10
24	12.2	0.6	10	7.1	1.2	10
26	10.3	0.4	10	6.9	0.8	10

Supplementary Table 4: Statistical analysis of glycemia variation between CD and KD male

		Glycemia change (%)			
Cohort	Mouse n°	CD_{12} Male	KD_{12} Male	CD_{26} Male	KD_{26} Male
A	1	1.6	-62.7	0.8	-49.4
	2	14.6	-82.6	-17.9	-75.0
	3	-23.8	-71.7	-18.2	-66.7
B	1	-29.2	-51.1	-15.9	-1.1
	2	19.6	-67.9	-5.2	-20.5
	3	35.7	-64.1	15.3	-17.4
C	1	9.3	-70.2	2.1	-29.0
	2	26.7	-60.8	14.4	-40.0
	3	-28.9	-70.1	-32.5	-37.6
	4	-11.0	-62.2	-3.7	-31.5

Test for normal distribution

**D'Agostino & Pearson test**	CD_{12} Male	KD_{12} Male	CD_{26} Male	KD_{26} Male
K2	1.9	1.4	0.1	0.2
P value	0.4	0.5	0.9	0.9
Passed normality test (alpha=0.05)?	Yes	Yes	Yes	Yes
P value summary	ns	ns	ns	ns

**Welch's ANOVA test**	
W (DFn, DFd)	53.0 (3.00, 18.3)
P value	<0.001
P value summary	***
Significant diff. among means (P < 0.05)?	Yes

**Dunnett's T3 multiple comparisons test**	**Mean Diff.**	**95.00% CI of diff.**	**Below threshold?**	**Summary**	**Adjusted P Value**
CD12 vs. KD12	67.8	42.9 to 92.7	Yes	***	<0.001
CD12 vs. CD26	7.5	-19.1 to 34.1	No	ns	0.9
CD12 vs. KD26	38.3	8.10 to 68.5	Yes	**	0.0
KD12 vs. CD26	-60.3	-76.8 to -43.7	Yes	***	<0.001
KD12 vs. KD26	-29.5	-53.3 to -5.75	Yes	*	0.0
CD26 vs. KD26	30.7	5.30 to 56.2	Yes	*	0.0

**Test details**	**Mean 1**	**Mean 2**	**Mean Diff.**	**SE of diff.**	**n1**	**n2**	**t**	**DF**
CD12 vs. KD12	1.5	-66.3	67.8	7.9	10.0	10.0	8.6	11.2
CD12 vs. CD26	1.5	-6.1	7.5	8.9	10.0	10.0	0.8	15.4
CD12 vs. KD26	1.5	-36.8	38.3	10.3	10.0	10.0	3.7	18.0
KD12 vs. CD26	-66.3	-6.1	-60.3	5.5	10.0	10.0	11.0	14.0
KD12 vs. KD26	-66.3	-36.8	-29.5	7.6	10.0	10.0	3.9	11.4
CD26 vs. KD26	-6.1	-36.8	30.7	8.6	10.0	10.0	3.6	15.8

**Descriptive statistics**	CD_{12} Male	KD_{12} Male	CD_{26} Male	KD_{26} Male
Number of values	10	10	10	10
Mean	1.5	-66.3	-6.1	-36.8
Std. Deviation	23.6	8.3	15.2	22.4
Std. Error of Mean	7.5	2.6	4.8	7.1

Supplementary Table 5: Statistical analysis of blood β-HB datasets from Safe's diet

	Blood β-HB / Mouse (mM)																			
	CD Male										KD Male									
Cohort	A			B			C				A			B			C			
Mouse n°	1	2	3	1	2	3	1	2	3	4	1	2	3	1	2	3	1	2	3	4
Day 1	0.4	0.5	0.3	0.3	0.5	0.4	0.4	0.6	0.5	0.5	0.5	0.3	0.5	0.4	0.3	0.4	0.4	0.6	0.5	0.3
3	1.0	0.8	0.3	0.9	0.7	0.5	0.4	0.6	0.5	0.5	5.6	3.1	3.8	3.1	4.3	3.5	0.9	2.2	0.6	0.6
5	0.3	0.2	0.3	0.5	0.4	0.6	1.1	0.6	0.6	0.6	3.6	6.6	4.8	4.6	5.9	6.9	3.5	5.8	2.6	3.8
8	0.3	0.4	0.3	0.4	0.4	0.4	0.4	0.6	0.3	0.5	6.0	6.7	5.8	2.7	2.7	7.9	3.4	6.8	3.5	5.6
10	0.4	0.4	0.5	0.6	0.4	0.4	0.4	0.2	0.5	0.4	3.7	3.6	3.6	8.0	1.9	8.0	5.2	8.0	4.6	8.0
12	0.3	0.3	0.4	0.4	0.4	0.4	0.6	0.3	0.4	0.3	6.7	8.0	8.0	7.8	3.8	8.0	4.5	4.6	3.7	5.2
15	0.8	0.6	0.5	0.3	0.3	0.3	0.4	0.3	0.6	0.5	5.4	7.0	5.7	2.2	1.9	1.6	2.9	6.8	4.8	8.0
17	0.6	0.5	0.5	0.4	0.3	0.4	0.4	0.3	0.5	0.5	3.2	7.4	3.9	3.7	5.9	8.0	4.9	5.6	6.2	4.8
19	0.4	0.4	0.4	0.4	0.4	0.4	0.6	0.3	0.3	0.4	2.3	2.4	1.1	3.1	4.6	3.8	0.7	1.2	0.9	1.3
22	0.6	0.3	0.3	0.4	0.4	0.4	0.4	0.4	0.6	0.3	3.1	4.2	3.0	5.2	6.4	8.0	1.6	1.4	2.1	1.9
24	0.4	0.4	0.2	0.4	0.6	0.4	0.4	0.3	0.7	0.7	3.2	5.0	3.4	7.1	5.1	6.8	1.4	0.8	1.3	1.6
26	0.3	0.2	0.3	0.5	0.3	0.4	0.2	0.3	0.2	0.2	4.3	6.4	3.7	2.2	1.1	0.8	1.1	1.4	1.3	1.6

Univariate Type III Repeated-Measures ANOVA Assuming Sphericity

```
Sum Sq num Df Error SS den Df  F value    Pr(>F)
(Intercept)    1109.83      1    83.37     18 239.6186 7.604e-12 ***
Treatment       705.55      1    83.37     18 152.3325 3.205e-10 ***
Time           154.25     11   286.00    198   9.7080 5.706e-14 ***
Treatment:Time  154.75     11   286.00    198   9.7398 5.141e-14 ***
---
Signif. codes:  0 '***' 0.001 '**' 0.01 '*' 0.05 '.' 0.1 ' ' 1
```

Mauchly Tests for Sphericity

```
Test statistic    p-value
Time              1.9092e-08 9.3473e-23
Treatment:Time     1.9092e-08 9.3473e-23
```

Greenhouse-Geisser and Huynh-Feldt Corrections
for Departure from Sphericity

```
GG eps Pr(>F[GG])
Time           0.31495  9.642e-06 ***
Treatment:Time 0.31495  9.317e-06 ***
---
Signif. codes:  0 '***' 0.001 '**' 0.01 '*' 0.05 '.' 0.1 ' ' 1

HF eps   Pr(>F[HF])
Time           0.3991825 9.072534e-07
Treatment:Time 0.3991825 8.691199e-07
```

Normal distribution: D'Agostino & Pearson test

**Control Diet datasets**	CD 1	CD 3	CD 5	CD 8	CD 10	CD 12	CD 15	CD 17	CD 19	CD 22	CD 24	CD 26
K2	0.2	0.7	5.9	3.1	3.0	8.8	1.4	0.2	10.4	2.3	0.6	3.2
P value	0.9	0.7	0.1	0.2	0.2	0.0	0.5	0.9	0.01	0.3	0.8	0.2
Passed normality test (alpha=0.05)?	Yes	Yes	Yes	Yes	Yes	No	Yes	Yes	No	Yes	Yes	Yes
P value summary	ns	ns	ns	ns	ns	*	ns	ns	**	ns	ns	ns
Number of values	10	10	10	10	10	10	10	10	10	10	10	10

**Ketogenic Diet datasets**	KD 1	KD 3	KD 5	KD 8	KD 10	KD 12	KD 15	KD 17	KD 19	KD 22	KD 24	KD 26
K2	0.6	0.3	1.1	2.2	2.9	5.7	2.5	0.5	1.4	1.8	1.9	5.3
P value	0.8	0.9	0.6	0.3	0.2	0.1	0.3	0.8	0.5	0.4	0.4	0.1
Passed normality test (alpha=0.05)?	Yes	Yes	Yes	Yes	Yes	Yes	Yes	Yes	Yes	Yes	Yes	Yes
P value summary	ns	ns	ns	ns	ns	ns	ns	ns	ns	ns	ns	ns
Number of values	10	10	10	10	10	10	10	10	10	10	10	10

**Type of Test**	Unpaired T-test	Welch's T-test	Welch's T-test	Welch's T-test	Welch's T-test	Mann-Whitney T-test	Welch's T-test	Welch's T-test	Mann-Whitney T-test	Welch's T-test	Welch's T-test	Welch's T-test
Data organization	CD 1 vs KD 1	CD 3 vs KD 3	CD 5 vs KD 5	CD 8 vs KD 8	CD 10 vs KD 10	CD 12 vs KD 12	CD 15 vs KD 15	CD 17 vs KD 17	CD 19 vs KD 19	CD 22 vs KD 22	CD 24 vs KD 24	CD 26 vs KD 26
Number of values	10 vs 10	10 vs 10	10 vs 10	10 vs 10	10 vs 10	10 vs 10	10 vs 10	10 vs 10	10 vs 10	10 vs 10	10 vs 10	10 vs 10
P value	0.66	0.003	<0.001	<0.001	<0.001	<0.001	<0.001	<0.001	<0.001	0.001	0.002	0.005
P value summary	ns	**	***	***	***	***	***	***	***	**	**	**
Significantly different (P < 0.05)?	No	Yes	Yes	Yes	Yes	Yes	Yes	Yes	Yes	Yes	Yes	Yes
One- or two-tailed P value?	Two-tailed	Two-tailed	Two-tailed	Two-tailed	Two-tailed	Two-tailed	Two-tailed	Two-tailed	Two-tailed	Two-tailed	Two-tailed	Two-tailed
t, df or *Sum of ranks in column I,U*	t=0.4472, df=18	t=4.011, df=9.323	t=9.204, df=9,546	t=7.913, df=9.045	t=6.803, df=9.035	*55 , 155*	t=5.637, df=9.097	t=9.873, df=9.068	*55 , 155*	t=4.652, df=9.044	t=4.224, df=9.090	*t=3.624, df=9.053*

**How big is the difference?**												
Mean of column A or *Median of column I*	0.44	0.62	0.52	0.4	0.42	*0.4000, n=10*	0.46	0.44	*0.4000, n=10*	0.41	0.45	0.29
Mean of column M or *Median of column U*	0.42	2.77	4.808	5.11	5.46	*5.950, n=10*	4.63	5.36	*1.800, n=10*	3.69	3.57	2.39
Difference between means (M - A) ± SEM or *Difference: Actual*	-0.02000 ± 0.04472	2.150 ± 0.5360	4.288 ± 0.4659	4.710 ± 0.5952	5.040 ± 0.7409	*5.55*	4.170 ± 0.7397	4.920 ± 0.4983	*1.4*	3.280 ± 0.7050	3.120 ± 0.7387	2.100 ± 0.5794
95% confidence interval or *Difference: Hodges-Lehmann*	-0.1140 to 0.07396	0.9438 to 3.356	3.243 to 5.333	3.364 to 6.056	3.365 to 6.715	*5.5*	2.499 to 5.841	3.794 to 6.046	*1.35*	1.686 to 4.874	1.452 to 4.788	0.7904 to 3.410
R squared (eta squared)	0.01099	0.6331	0.8987	0.8738	0.8367		0.7775	0.9149		0.7053	0.6625	0.592

**F test to compare variances**												
F, DFn, Dfd	1.143, 9, 9	55.70, 9, 9	32.91, 9, 9	397.6, 9, 9	513.6, 9, 9	404.0, 9, 9	185.5, 9, 9	265.0, 9, 9	268.4, 9, 9	409.4, 9, 9	199.4, 9, 9	338.5, 9, 9
P value	0.85	<0.001	<0.001	<0.001	<0.001	<0.001	<0.001	<0.001	<0.001	<0.001	<0.001	<0.001
P value summary	ns	***	***	***	***	***	***	***	***	***	***	***
Significantly different (P < 0.05)?	No	Yes	Yes	Yes	Yes	Yes	Yes	Yes	Yes	Yes	Yes	Yes

**Descriptive statistics**	CD Male			KD Male		
Days	Mean	SEM	N	Mean	SEM	N
1	0.4	0.03	10	0.4	0.03	10
3	0.6	0.07	10	2.8	0.5	10
5	0.5	0.08	10	4.8	0.5	10
8	0.4	0.03	10	5.1	0.6	10
10	0.4	0.03	10	5.5	0.7	10
12	0.4	0.03	10	6.0	0.6	10
15	0.5	0.05	10	4.6	0.7	10
17	0.4	0.03	10	5.4	0.5	10
19	0.4	0.03	10	2.1	0.4	10
22	0.4	0.03	10	3.7	0.7	10
24	0.5	0.05	10	3.6	0.7	10
26	0.3	0.03	10	2.4	0.6	10

Supplementary Table 6: Statistical analysis of blood β-HB variation between CD and KD male

		Blood β-HB change (%)			
Cohort	Mouse n°	CD_{12} Male	KD_{12} Male	CD_{26} Male	KD_{26} Male
A	1	-25.0	1240.0	-25	760
	2	-40.0	2566.7	-60	2033.3
	3	33.3	1500.0	0	640
B	1	33.3	1850.0	66.7	450
	2	-20.0	1166.7	-40	266.7
	3	0.0	1900.0	0	100
C	1	50.0	1025.0	-50	175
	2	-50.0	666.7	-50	133
	3	-20.0	640.0	-60	160
	4	-40.0	1633.3	-60	433

Test for normal distribution

**D'Agostino & Pearson test**	CD_{12} Male	KD_{12} Male	CD_{26} Male	KD_{26} Male
K2	1.7	0.56	8.0	18.0
P value	0.42	0.75	0.0	<0.001
Passed normality test (alpha=0.05)?	Yes	Yes	No	No
P value summary	ns	ns	*	***

**Kruskal-Wallis test**	
P value	<0.001
Exact or approximate P value?	Approximate
P value summary	***
Do the medians vary signif. (P < 0.05)?	Yes
Number of groups	4
Kruskal-Wallis statistic	32.1

**Dunn's multiple comparisons test**	**Mean rank diff.**	**Significant?**	**Summary**	**Adjusted P Value**
CD12 vs. KD12	-21.8	Yes	***	<0.001
CD12 vs. CD26	4.1	No	ns	>0.99
CD12 vs. KD26	-14.1	Yes	*	0.04
KD12 vs. CD26	25.9	Yes	***	<0.001
KD12 vs. KD26	7.7	No	ns	0.8
CD26 vs. KD26	-18.2	Yes	**	0.003

**Test details**	**Mean rank 1**	**Mean rank 2**	**Mean rank diff.**	**n1**	**n2**	**Z**
CD12 vs. KD12	12.6	34.4	-21.8	10.0	10.0	4.2
CD12 vs. CD26	12.6	8.5	4.1	10.0	10.0	0.8
CD12 vs. KD26	12.6	26.7	-14.1	10.0	10.0	2.7
KD12 vs. CD26	34.4	8.5	25.9	10.0	10.0	5.0
KD12 vs. KD26	34.4	26.7	7.7	10.0	10.0	1.5
CD26 vs. KD26	8.5	26.7	-18.2	10.0	10.0	3.5

**Descriptive statistics**	CD_{12} Male	KD_{12} Male	CD_{26} Male	KD_{26} Male
Number of values	10	10	10	10
Mean	-7.8	1419.0	-28.0	515.0
Std. Deviation	35.0	597.0	40.0	579.0
Std. Error of Mean	11.0	189.0	13.0	183.0

Supplementary Table 7: Statistical analysis of blood lactate datasets from Safe's diet

		Blood lactate / Mouse (mM)																			
		CD Male										KD Male									
	Cohort	A			B			C				A			B			C			
	Mouse n°	1	2	3	1	2	3	1	2	3	4	1	2	3	1	2	3	1	2	3	4
Day	1	3.8	3.7	3.3	3.6	2.7	4.0	4.7	4.3	3.7	4.1	4.6	3.4	5.9	3.4	2.7	2.5	2.8	3.4	3.2	3.3
	3	4.2	3.3	3.7	2.8	2.7	3.0	2.8	3.2	4.3	2.6	2.3	3.9	5.4	7.3	6.4	7.9	3.8	3.2	0.7	3.9
	5	3.3	6.1	2.8	4.2	2.7	4.2	2.1	2.7	2.4	3.4	2.6	1.8	2.8	2.3	3.1	1.9	2.3	4.1	4.0	2.9
	8	5.0	6.0	4.1	2.5	3.2	2.9	3.7	2.9	5.7	2.3	2.1	1.6	3.6	2.2	2.1	2.3	3.1	1.9	3.1	2.5
	10	3.3	4.2	3.0	4.0	2.7	3.3	4.0	2.7	4.3	3.1	7.8	7.9	7.7	3.5	2.3	1.8	2.8	3.2	2.5	3.7
	12	3.7	5.3	4.7	3.1	2.5	2.7	2.1	2.7	2.8	1.9	2.3	2.8	2.6	1.9	4.4	1.8	2.3	1.8	2.7	2.7
	15	3.9	5.3	2.2	2.7	2.1	3.7	3.5	2.6	2.5	2.0	3.3	4.3	3.8	2.3	2.3	3.5	2.6	5.4	2.3	2.3
	17	4.8	3.8	4.1	2.9	2.9	3.9	3.3	2.7	3.4	2.5	5.8	3.0	2.5	1.9	2.9	2.4	2.8	3.2	3.5	3.4
	19	3.0	3.1	2.1	2.0	2.0	2.7	5.5	2.6	2.6	4.9	3.1	2.8	7.4	2.8	2.4	2.5	3.2	3.1	3.4	4.5
	22	3.1	3.5	2.6	1.6	2.9	2.2	2.3	2.9	2.5	3.1	2.9	3.0	3.3	2.5	2.5	2.5	2.8	3.0	2.3	2.9
	24	4.1	3.0	3.2	2.7	2.8	3.0	2.7	2.9	2.3	2.1	2.7	4.7	1.7	4.1	3.9	5.8	2.8	2.3	2.9	2.9
	26	2.9	3.3	2.0	3.6	5.9	2.3	3.3	3.0	2.5	3.3	3.2	2.4	3.5	3.0	3.7	3.4	1.9	2.4	2.1	2.0

```
Univariate Type III Repeated-Measures ANOVA Assuming Sphericity

Sum Sq num Df   Error SS den Df   F value  Pr(>F)
(Intercept)    4.0851e+11       1 2.1714e+09     18 3386.4085 < 2e-16 ***
Treatment      5.5288e+08       1 2.1714e+09     18    4.5831 0.04623 *
Time           1.4163e+09      11 3.4127e+10    198    0.7470 0.69231
Treatment:Time 2.9535e+09      11 3.4127e+10    198    1.5578 0.11384
---
Signif. codes:  0 ‘***’ 0.001 ‘**’ 0.01 ‘*’ 0.05 ‘.’ 0.1 ‘ ’ 1


Mauchly Tests for Sphericity

Test statistic    p-value
Time               8.5821e-08 2.0761e-19
Treatment:Time     8.5821e-08 2.0761e-19


Greenhouse-Geisser and Huynh-Feldt Corrections
for Departure from Sphericity

GG eps Pr(>F[GG])
Time           0.55046     0.6142
Treatment:Time 0.55046     0.1658

HF eps Pr(>F[HF])
Time           0.8603487  0.6720403
Treatment:Time 0.8603487  0.1276091
```

Normal distribution: D'Agostino & Pearson test

**Control Diet datasets**	CD 1	CD 3	CD 5	CD 8	CD 10	CD 12	CD 15	CD 17	CD 19	CD 22	CD 24	CD 26
K2	1.2	1.7	6.5	1.6	3.0	2.6	3.7	0.7	4.8	0.8	5.1	12.9
P value	0.5	0.4	0.04	0.5	0.2	0.3	0.2	0.7	0.1	0.7	0.1	0.002
Passed normality test (alpha=0.05)?	Yes	Yes	No	Yes	Yes	Yes	Yes	Yes	Yes	Yes	Yes	No
P value summary	ns	ns	*	ns	ns	ns	ns	ns	ns	ns	ns	**
Number of values	10	10	10	10	10	10	10	10	10	10	10	10

**Ketogenic Diet datasets**	KD 1	KD 3	KD 5	KD 8	KD 10	KD 12	KD 15	KD 17	KD 19	KD 22	KD 24	KD 26
K2	9.1	0.1	1.0	1.0	2.8	11.1	2.5	13.7	18.0	0.3	1.4	3.6
P value	0.01	0.9	0.6	0.6	0.3	0.004	0.3	0.001	<0.001	0.9	0.5	0.2
Passed normality test (alpha=0.05)?	No	Yes	Yes	Yes	Yes	No	Yes	No	No	Yes	Yes	Yes
P value summary	*	ns	ns	ns	ns	**	ns	**	***	ns	ns	ns
Number of values	10	10	10	10	10	10	10	10	10	10	10	10

**Type of Test**	Mann-Whitney T-test	Welch's T-test	Mann-Whitney T-test	Welch's T-test	Welch's T-test	Mann-Whitney T-test	Unpaired T-test	Mann-Whitney T-test	Mann-Whitney T-test	Unpaired T-test	Welch's T-test	Mann-Whitney T-test
Data organization	CD 1 vs KD 1	CD 3 vs KD 3	CD 5 vs KD 5	CD 8 vs KD 8	CD 10 vs KD 10	CD 12 vs KD 12	CD 15 vs KD 15	CD 17 vs KD 17	CD 19 vs KD 19	CD 22 vs KD 22	CD 24 vs KD 24	CD 26 vs KD 26
Number of values	10 vs 10	10 vs 10	10 vs 10	10 vs 10	10 vs 10	10 vs 10	10 vs 10	10 vs 10	10 vs 10	10 vs 10	10 vs 10	10 vs 10
P value	0.12	0.13	0.2	0.01	0.31	0.12	0.74	0.27	0.21	0.62	0.26	0.47
P value summary	ns	ns	ns	*	ns	ns	ns	ns	ns	ns	ns	ns
Significantly different (P < 0.05)?	No	No	No	Yes	No	No	No	No	No	No	No	No
One- or two-tailed P value?	Two-tailed	Two-tailed	Two-tailed	Two-tailed	Two-tailed	Two-tailed	Two-tailed	Two-tailed	Two-tailed	Two-tailed	Two-tailed	Two-tailed
t, df or *Sum of ranks in column I,U*	*126 , 84*	t=1.647, df=10.32	*122.5 , 87.50*	t=2.970, df=12.80	t=1.070, df=10.11	*126 , 84*	t=0.3405, df=18	*120 , 90*	*88 , 122*	t=0.5027, df=18	t=1.176, df=12.35	*115 , 95*

**How big is the difference?**												
Mean of column A or *Median of column I*	*3.750, n=10*	3.26	*3.050, n=10*	3.83	3.46	*2.750, n=10*	03.05	*3.350, n=10*	*2.650, n=10*	2.67	2.88	*3.150, n=10*
Mean of column M or *Median of column U*	*3.350, n=10*	4.48	*2.700, n=10*	2.45	4.32	*2.450, n=10*	3.21	*2.950, n=10*	*3.100, n=10*	2.77	3.38	*2.700, n=10*
Difference between means (M - A) ± SEM or *Difference: Actual*	*-0.4*	1.220 ± 0.7409	*-0.35*	-1.380 ± 0.4646	0.8600 ± 0.8035	*-0.3*	0.1600 ± 0.4699	*-0.4*	*0.45*	0.1000 ± 0.1989	0.5000 ± 0.4252	*-0.45*
95% confidence interval or *Difference: Hodges-Lehmann*	*-0.5*	-0.4237 to 2.864	*-0.4*	-2.385 to -0.3747	-0.9276 to 2.648	*-0.4*	-0.8273 to 1.147	*-0.4*	*0.4*	-0.3180 to 0.5180	-0.4235 to 1.424	*-0.25*
R squared (eta squared)		0.208		0.408	0.1018		0.006399			0.01384	0.1007	

**F test to compare variances**												
F, DFn, Dfd	3.417, 9, 9	13.51, 9, 9	2.261, 9, 9	4.511, 9, 9	16.17, 9, 9	2.104, 9, 9	1.035, 9, 9	2.138, 9, 9	1.517, 9, 9	3.137, 9, 9	5.173, 9, 9	2.522, 9, 9
P value	0.08	<0.001	0.24	0.04	<0.001	0.28	0.96	0.27	0.54	0.1	0.02	0.18
P value summary	ns	***	ns	*	***	ns	ns	ns	ns	ns	*	ns
Significantly different (P < 0.05)?	No	Yes	No	Yes	Yes	No	No	No	No	No	Yes	No

**Descriptive statistics**	CD Male			KD Male		
Days	Mean	SEM	N	Mean	SEM	N
1	3.8	0.2	10	3.5	0.3	10
3	3.3	0.2	10	4.5	0.7	10
5	3.4	0.4	10	2.8	0.2	10
8	3.8	0.4	10	2.5	0.2	10
10	3.5	0.2	10	4.3	0.8	10
12	3.2	0.3	10	2.5	0.2	10
15	3.1	0.3	10	3.2	0.3	10
17	3.4	0.2	10	3.1	0.3	10
19	3.1	0.4	10	3.5	0.5	10
22	2.7	0.2	10	2.8	0.1	10
24	2.9	0.2	10	3.4	0.4	10
26	3.2	0.3	10	2.8	0.2	10

Supplementary Table 8: Statistical analysis of blood lacate variation between CD and KD male

		Blood lactate change (%)			
Cohort	Mouse n°	CD_{12} Male	KD_{12} Male	CD_{26} Male	KD_{26} Male
A	1	-2.6	-50.0	-23.7	-30.4
	2	43.2	-17.6	-10.8	-29.4
	3	42.4	-55.9	-39.4	-40.7
B	1	-13.9	-44.1	0	-11.8
	2	-7.4	63.0	118.5	37
	3	-32.5	-28.0	-42.5	36
C	1	-55.3	-17.9	-30	-32
	2	-37.2	-47.1	-30	-29
	3	-24.3	-15.6	-32	-34
	4	-53.7	-18.2	-20	-39

Test for normal distribution

**D'Agostino & Pearson test**	CD_{12} Male	KD_{12} Male	CD_{26} Male	KD_{26} Male
K2	1.2	14	23.0	5.2
P value	0.56	<0.001	<0.001	0.1
Passed normality test (alpha=0.05)?	Yes	No	No	Yes
P value summary	ns	***	***	ns

**Kruskal-Wallis test**	
P value	0.88
Exact or approximate P value?	Approximate
P value summary	ns
Do the medians vary signif. (P < 0.05)?	No
Number of groups	4
Kruskal-Wallis statistic	0.681

**Dunn's multiple comparisons test**	**Mean rank diff.**	**Significant?**	**Summary**	**Adjusted P Value**
CD12 vs. KD12	3.8	No	ns	>0.99
CD12 vs. CD26	0.3	No	ns	>0.99
CD12 vs. KD26	2.0	No	ns	>0.99
KD12 vs. CD26	-3.6	No	ns	>0.99
KD12 vs. KD26	-1.9	No	ns	>0.99
CD26 vs. KD26	1.7	No	ns	>0.99

**Test details**	**Mean rank 1**	**Mean rank 2**	**Mean rank diff.**	**n1**	**n2**	**Z**
CD12 vs. KD12	22.0	18.2	3.8	10.0	10.0	0.7
CD12 vs. CD26	22.0	21.8	0.3	10.0	10.0	0.0
CD12 vs. KD26	22.0	20.1	2.0	10.0	10.0	0.4
KD12 vs. CD26	18.2	21.8	-3.6	10.0	10.0	0.7
KD12 vs. KD26	18.2	20.1	-1.9	10.0	10.0	0.4
CD26 vs. KD26	21.8	20.1	1.7	10.0	10.0	0.3

**Descriptive statistics**	CD_{12} Male	KD_{12} Male	CD_{26} Male	KD_{26} Male
Number of values	10	10	10	10
Mean	-14.0	-23.0	-11.0	-17.0
Std. Deviation	35.0	34.0	47.0	29.0
Std. Error of Mean	11.0	11.0	15.0	9.3

Supplementary Table 9: Statistical analysis of male's body weight datasets

		Body weight / Mouse (g)																			
		CD Male										KD Male									
	Cohort	A			B			C				A			B			C			
	Mouse n°	1	2	3	1	2	3	1	2	3	4	1	2	3	1	2	3	1	2	3	4
Day	1	21.0	23.5	24.8	25.5	24.8	22.1	22.4	22.8	20.4	24.9	24.3	24.1	22.3	23.5	22.9	23.8	24.0	23.1	23.5	22.6
	3	21.7	24.4	26.1	25.3	25.1	21.8	22.7	23.3	21.4	25.8	22.4	23.1	20.5	22.7	21.4	22.2	22.7	20.4	21.7	20.2
	5	21.5	24.6	26.0	24.4	24.1	22.3	22.7	23.5	21.9	26.0	20.9	21.7	19.6	22.1	20.7	21.2	21.4	20.1	21.1	20.0
	8	21.6	24.8	26.6	25.4	25.0	22.0	22.3	23.4	21.4	26.1	19.9	20.9	18.7	21.9	20.2	21.0	21.4	20.1	20.1	19.9
	10	21.4	24.7	27.4	25.0	24.9	22.3	22.2	23.1	21.4	26.0	19.3	20.3	18.2	21.3	19.9	20.5	21.2	19.3	19.8	19.5
	12	21.7	25.0	27.7	26.6	26.4	23.6	23.3	24.1	22.4	27.0	19.3	19.9	17.8	20.9	19.2	20.1	21.5	19.4	18.3	19.7
	15	21.7	24.7	27.4	27.3	26.9	24.5	23.3	24.1	21.9	27.0	19.1	19.8	17.5	21.0	19.5	19.9	21.3	18.8	19.6	19.2
	17	21.7	25.3	28.1	27.1	26.3	24.6	23.4	23.9	21.9	26.8	19.2	19.7	17.4	21.0	19.4	19.8	21.7	18.9	19.9	19.1
	19	22.2	25.9	28.5	28.1	27.4	25.3	24.3	24.4	22.3	27.8	19.4	19.9	17.2	21.2	19.4	20.0	21.9	18.8	19.6	18.7
	22	21.9	25.8	28.1	28.6	27.1	25.6	24.3	24.4	22.5	27.5	19.4	19.9	17.3	20.7	19.5	19.7	22.3	18.9	19.6	18.6
	24	22.1	25.8	28.3	27.7	27.0	25.7	24.6	24.7	22.4	28.2	19.1	19.9	17.3	20.6	19.3	19.7	22.5	19.1	20.0	18.9
	26	22.4	26.9	29.3	28.7	27.1	26.3	25.8	25.4	23.2	28.6	19.2	19.9	17.5	20.5	18.9	19.7	22.6	19.3	20.2	18.7

Univariate Type III Repeated-Measures ANOVA Assuming Sphericity

```
Sum Sq num Df Error SS den Df  F value    Pr(>F)
(Intercept)    121415      1   547.87     18 3989.035 < 2.2e-16 ***
Treatment        1150      1   547.87     18   37.775 8.367e-06 ***
Time               36     11    49.60    198   13.114 < 2.2e-16 ***
Treatment:Time    228     11    49.60    198   82.818 < 2.2e-16 ***
---
Signif. codes:  0 '***' 0.001 '**' 0.01 '*' 0.05 '.' 0.1 ' ' 1
```

Mauchly Tests for Sphericity

```
Test statistic    p-value
Time              3.6715e-07 2.7527e-16
Treatment:Time    3.6715e-07 2.7527e-16
```

Greenhouse-Geisser and Huynh-Feldt Corrections
for Departure from Sphericity

```
GG eps Pr(>F[GG])
Time           0.24098  5.197e-06 ***
Treatment:Time 0.24098  < 2.2e-16 ***
---
Signif. codes:  0 '***' 0.001 '**' 0.01 '*' 0.05 '.' 0.1 ' ' 1

HF eps    Pr(>F[HF])
Time           0.2864554 8.870669e-07
Treatment:Time 0.2864554 4.222661e-21
```

Normal distribution: D'Agostino & Pearson test

**Control Diet datasets**	CD 1	CD 3	CD 5	CD 8	CD 10	CD 12	CD 15	CD 17	CD 19	CD 22	CD 24	CD 26
K2	1.3	3.5	1.0	3.4	0.8	1.7	2.0	1.0	1.4	0.9	0.9	0.7
P value	0.5	0.2	0.6	0.2	0.7	0.4	0.4	0.6	0.5	0.6	0.6	0.7
Passed normality test (alpha=0.05)?	Yes	Yes	Yes	Yes	Yes	Yes	Yes	Yes	Yes	Yes	Yes	Yes
P value summary	ns	ns	ns	ns	ns	ns	ns	ns	ns	ns	ns	ns
Number of values	10	10	10	10	10	10	10	10	10	10	10	10

**Ketogenic Diet datasets**	KD 1	KD 3	KD 5	KD 8	KD 10	KD 12	KD 15	KD 17	KD 19	KD 22	KD 24	KD 26
K2	1.0	2.3	0.3	0.3	0.2	0.0	0.7	1.1	0.6	2.7	3.3	4.6
P value	0.6	0.3	0.9	0.9	0.9	1.0	0.7	0.6	0.7	0.3	0.2	0.1
Passed normality test (alpha=0.05)?	Yes	Yes	Yes	Yes	Yes	Yes	Yes	Yes	Yes	Yes	Yes	Yes
P value summary	ns	ns	ns	ns	ns	ns	ns	ns	ns	ns	ns	ns
Number of values	10	10	10	10	10	10	10	10	10	10	10	10

**Type of Test**	Welch's T-test	Unpaired T-test	Welch's T-test	Welch's T-test	Welch's T-test	Unpaired T-test	Welch's T-test	Unpaired T-test	Unpaired T-test	Unpaired T-test	Unpaired T-test	Unpaired T-test
Data organization	CD 1 vs KD 1	CD 3 vs KD 3	CD 5 vs KD 5	CD 8 vs KD 8	CD 10 vs KD 10	CD 12 vs KD 12	CD 15 vs KD 15	CD 17 vs KD 17	CD 19 vs KD 19	CD 22 vs KD 22	CD 24 vs KD 24	CD 26 vs KD 26
Number of values	10 vs 10	10 vs 10	10 vs 10	10 vs 10	10 vs 10	10 vs 10	10 vs 10	10 vs 10	10 vs 10	10 vs 10	10 vs 10	10 vs 10
P value	0.77	0.006	<0.001	<0.001	<0.001	<0.001	<0.001	<0.001	<0.001	<0.001	<0.001	<0.001
P value summary	ns	**	***	***	***	***	***	***	***	***	***	***
Significantly different (P < 0.05)?	No	Yes	Yes	Yes	Yes	Yes	Yes	Yes	Yes	Yes	Yes	Yes
One- or two-tailed P value?	Two-tailed	Two-tailed	Two-tailed	Two-tailed	Two-tailed	Two-tailed	Two-tailed	Two-tailed	Two-tailed	Two-tailed	Two-tailed	Two-tailed
t, df	t=0.2970, df=11.56	t=3.116, df=18	t=4.978, df=13.01	t=5.058, df=12.76	t=5.494, df=12.59	t=6.978, df=18	t=6.917, df=12.97	t=6.747, df=18	t=7.127, df=18	t=7.210, df=18	t=7.304, df=18	t=8.004, df=18

**How big is the difference?**												
Mean of column A	23.22	23.75	23.68	23.84	23.82	24.76	24.88	24.89	25.6	25.57	25.61	26.34
Mean of column M	23.4	21.71	20.86	20.39	19.91	19.6	19.55	19.59	19.62	19.56	19.62	19.64
Difference between means (M - A) ± SEM	0.1770 ± 0.5959	-2.040 ± 0.6548	-2.829 ± 0.5683	-3.454 ± 0.6828	-3.915 ± 0.7126	-5.161 ± 0.7396	-5.330 ± 0.7706	-5.303 ± 0.7860	-5.982 ± 0.8393	-6.010 ± 0.8336	-5.995 ± 0.8208	-6.700 ± 0.8371
95% confidence interval	-1.127 to 1.481	-3.416 to -0.6644	-4.057 to -1.601	-4.932 to -1.976	-5.460 to -2.370	-6.715 to -3.607	-6.995 to -3.665	-6.954 to -3.652	-7.745 to -4.219	-7.761 to -4.259	-7.719 to -4.271	-8.459 to -4.941
R squared (eta squared)	0.007572	0.3503	0.6557	0.6672	0.7057	0.7301	0.7867	0.7166	0.7383	0.7428	0.7477	0.7807

**F test to compare variances**												
F, DFn, Dfd	6.877, 9, 9	2.802, 9, 9	4.254, 9, 9	4.563, 9, 9	4.812, 9, 9	3.651, 9, 9	4.297, 9, 9	3.490, 9, 9	3.160, 9, 9	3.059, 9, 9	2.796, 9, 9	2.930, 9, 9
P value	0.008	0.14	0.04	0.03	0.03	0.07	0.04	0.08	0.1	0.11	0.14	0.13
P value summary	**	ns	*	*	*	ns	*	ns	ns	ns	ns	ns
Significantly different (P < 0.05)?	Yes	No	Yes	Yes	Yes	No	Yes	No	No	No	No	No

**Descriptive statistics**	CD Male			KD Male		
Days	Mean	SEM	N	Mean	SEM	N
1	23.2	0.6	10	23.4	0.2	10
3	23.7	0.6	10	21.7	0.3	10
5	23.7	0.5	10	20.9	0.2	10
8	23.8	0.6	10	20.4	0.3	10
10	23.8	0.6	10	19.9	0.3	10
12	24.8	0.7	10	19.6	0.3	10
15	24.9	0.7	10	19.6	0.3	10
17	24.9	0.7	10	19.6	0.4	10
19	25.6	0.7	10	19.6	0.4	10
22	25.6	0.7	10	19.6	0.4	10
24	25.6	0.7	10	19.6	0.4	10
26	26.3	0.7	10	19.6	0.4	10

Supplementary Table 10: Statistical analysis of female's body weight datasets

		Body weight / Mouse (g)																			
		CD Female										KD Female									
	Cohort	A			B			C				A			B			C			
	Mouse n°	1	2	3	1	2	3	1	2	3	4	1	2	3	1	2	3	1	2	3	4
Day	1	19.5	17.6	19.3	18.9	18.8	17.6	18.5	18.4	17.0	19.2	19.3	18.1	18.2	17.8	19.3	17.9	16.5	18.3	18.8	18.6
	3	19.2	16.7	18.8	18.9	19.6	17.2	18.7	19.2	17.7	18.8	17.3	16.9	16.4	16.5	19.0	16.9	14.7	17.4	17.6	17.7
	5	18.9	16.8	18.7	18.3	19.3	16.7	19.3	19.4	18.3	19.1	15.8	15.8	14.7	15.8	18.2	16.0	13.3	16.7	17.0	16.6
	8	18.8	17.3	18.6	17.9	18.6	16.7	18.7	18.9	17.8	19.1	15.3	15.9	14.1	15.3	18.8	14.9	13.5	16.7	16.5	16.3
	10	18.8	17.0	19.1	18.2	19.7	17.2	19.2	18.1	17.4	19.0	14.9	15.5	13.5	15.2	19.0	14.0	13.1	16.4	16.2	16.0
	12	19.5	17.6	19.6	17.7	19.0	17.2	18.9	19.0	17.7	19.5	14.7	15.4	13.4	14.9	19.3	13.8	12.3	16.4	16.1	15.4
	15	19.9	17.7	19.9	18.0	19.7	17.6	20.0	19.0	17.4	19.6	14.7	15.3	12.7	14.8	19.8	12.8	11.5	17.1	15.7	15.3
	17	20.1	18.4	19.9	18.1	19.7	17.5	19.4	19.1	17.6	19.6	14.4	15.2	12.1	15.1	20.0	12.3	11.2	17.0	15.8	15.0
	19	20.7	18.6	21.3	18.3	20.5	18.0	20.3	21.0	17.9	20.3	14.9	15.5	12.2	15.4	19.9	11.8	11.6	17.0	16.0	15.2
	22	20.3	18.9	20.9	18.0	21.3	18.8	20.0	20.6	18.8	20.1	15.1	16.3	12.0	15.7	19.5	11.1	11.6	17.3	15.9	15.3
	24	20.7	19.2	21.5	18.8	21.4	18.6	20.2	21.0	18.3	20.2	14.6	16.2	11.7	15.6	19.6	11.0	11.4	17.8	15.8	14.8
	26	20.8	19.0	22.6	18.7	22.4	19.6	20.2	20.0	19.3	21.1	14.8	16.4	12.0	15.8	19.4	11.1	11.8	17.9	16.2	15.2

Univariate Type III Repeated-Measures ANOVA Assuming Sphericity

```
Sum Sq num Df Error SS den Df   F value    Pr(>F)
(Intercept)    71923      1   498.44     18 2597.3452 < 2.2e-16 ***
Treatment        702      1   498.44     18   25.3558 8.599e-05 ***
Time              47     11   103.76    198    8.1856 9.337e-12 ***
Treatment:Time   135     11   103.76    198   23.4717 < 2.2e-16 ***
---
Signif. codes:  0 '***' 0.001 '**' 0.01 '*' 0.05 '.' 0.1 ' ' 1
```

Mauchly Tests for Sphericity

```
Test statistic    p-value
Time              5.2943e-09 1.0821e-25
Treatment:Time    5.2943e-09 1.0821e-25
```

Greenhouse-Geisser and Huynh-Feldt Corrections
for Departure from Sphericity

```
GG eps Pr(>F[GG])
Time           0.14895   0.002567 **
Treatment:Time 0.14895  2.657e-06 ***
---
Signif. codes:  0 '***' 0.001 '**' 0.01 '*' 0.05 '.' 0.1 ' ' 1

HF eps   Pr(>F[HF])
Time           0.1618552 1.886948e-03
Treatment:Time 0.1618552 1.125035e-06
```

Normal distribution: D'Agostino & Pearson test

**Control Diet datasets**	CD 1	CD 3	CD 5	CD 8	CD 10	CD 12	CD 15	CD 17	CD 19	CD 22	CD 24	CD 26
K2	1.1	2.0	2.9	2.1	1.6	3.9	5.3	2.4	4.2	1.0	2.4	1.1
P value	0.6	0.4	0.2	0.4	0.4	0.1	0.1	0.3	0.1	0.6	0.3	0.6
Passed normality test (alpha=0.05)?	Yes	Yes	Yes	Yes	Yes	Yes	Yes	Yes	Yes	Yes	Yes	Yes
P value summary	ns	ns	ns	ns	ns	ns	ns	ns	ns	ns	ns	ns
Number of values	10	10	10	10	10	10	10	10	10	10	10	10

**Ketogenic Diet datasets**	KD 1	KD 3	KD 5	KD 8	KD 10	KD 12	KD 15	KD 17	KD 19	KD 22	KD 24	KD 26
K2	3.4	3.2	1.6	1.6	2.6	3.3	1.4	1.2	0.5	0.1	0.2	0.4
P value	0.2	0.2	0.5	0.5	0.3	0.2	0.5	0.6	0.8	1.0	0.9	0.8
Passed normality test (alpha=0.05)?	Yes	Yes	Yes	Yes	Yes	Yes	Yes	Yes	Yes	Yes	Yes	Yes
P value summary	ns	ns	ns	ns	ns	ns	ns	ns	ns	ns	ns	ns
Number of values	10	10	10	10	10	10	10	10	10	10	10	10

**Type of Test**	Unpaired T-test	Unpaired T-test	Unpaired T-test	Unpaired T-test	Unpaired T-test	Welch's T-test	Welch's T-test	Welch's T-test	Unpaired T-test	Welch's T-test	Welch's T-test	Unpaired T-test
Data organization	CD 1 vs KD 1	CD 3 vs KD 3	CD 5 vs KD 5	CD 8 vs KD 8	CD 10 vs KD 10	CD 12 vs KD 12	CD 15 vs KD 15	CD 17 vs KD 17	CD 19 vs KD 19	CD 22 vs KD 22	CD 24 vs KD 24	CD 26 vs KD 26
Number of values	10 vs 10	10 vs 10	10 vs 10	10 vs 10	10 vs 10	10 vs 10	10 vs 10	10 vs 10	10 vs 10	10 vs 10	10 vs 10	10 vs 10
P value	0.57	0.006	<0.001	<0.001	<0.001	<0.001	<0.001	<0.001	<0.001	<0.001	<0.001	<0.001
P value summary	ns	**	***	***	***	***	***	***	***	***	***	***
Significantly different (P < 0.05)?	No	Yes	Yes	Yes	Yes	Yes	Yes	Yes	Yes	Yes	Yes	Yes
One- or two-tailed P value?	Two-tailed	Two-tailed	Two-tailed	Two-tailed	Two-tailed	Two-tailed	Two-tailed	Two-tailed	Two-tailed	Two-tailed	Two-tailed	Two-tailed
t, df	t=0.5769, df=18	t=3.118, df=18	t=4.790, df=18	t=4.704, df=18	t=4.856, df=18	t=5.051, df=12.81	t=4.760, df=12.58	t=4.754, df=11.46	t=5.202, df=18	t=5.253, df=11.81	t=5.298, df=12.14	t=5.541, df=18

**How big is the difference?**												
Mean of column A	18.47	18.46	18.47	18.2	18.35	18.54	18.87	18.93	19.67	19.75	19.96	20.35
Mean of column M	18.26	17.02	15.96	15.7	15.37	15.16	14.95	14.79	14.92	14.95	14.84	15.04
Difference between means (M - A) ± SEM	-0.2150 ± 0.3727	-1.442 ± 0.4625	-2.508 ± 0.5236	-2.500 ± 0.5315	-2.981 ± 0.6138	-3.380 ± 0.6691	-3.920 ± 0.8235	-4.142 ± 0.8713	-4.745 ± 0.9122	-4.800 ± 0.9138	-5.115 ± 0.9655	-5.305 ± 0.9573
95% confidence interval	-0.9979 to 0.5679	-2.414 to -0.4703	-3.608 to -1.408	-3.617 to -1.383	-4.271 to -1.691	-4.828 to -1.932	-5.705 to -2.135	-6.050 to -2.234	-6.661 to -2.829	-6.795 to -2.805	-7.216 to -3.014	-7.316 to -3.294
R squared (eta squared)	0.01816	0.3506	0.5604	0.5514	0.5671	0.6657	0.6429	0.6635	0.6005	0.7002	0.6981	0.6304

**F test to compare variances**												
F, DFn, Dfd	1.025, 9, 9	1.376, 9, 9	1.815, 9, 9	3.631, 9, 9	3.339, 9, 9	4.498, 9, 9	4.816, 9, 9	7.167, 9, 9	3.692, 9, 9	6.244, 9, 9	5.556, 9, 9	3.956, 9, 9
P value	0.97	0.64	0.39	0.07	0.09	0.04	0.03	0.007	0.06	0.01	0.02	0.05
P value summary	ns	ns	ns	ns	ns	*	*	**	ns	*	*	ns
Significantly different (P < 0.05)?	No	No	No	No	No	Yes	Yes	Yes	No	Yes	Yes	No

**Descriptive statistics**	CD Female			KD Female		
Days	Mean	SEM	N	Mean	SEM	N
1	18.5	0.3	10	18.3	0.3	10
3	18.5	0.3	10	17.0	0.4	10
5	18.5	0.3	10	16.0	0.4	10
8	18.2	0.2	10	15.7	0.5	10
10	18.4	0.3	10	15.4	0.5	10
12	18.5	0.3	10	15.2	0.6	10
15	18.9	0.3	10	14.9	0.7	10
17	18.9	0.3	10	14.8	0.8	10
19	19.7	0.4	10	14.9	0.8	10
22	19.8	0.3	10	15.0	0.8	10
24	20.0	0.4	10	14.8	0.9	10
26	20.3	0.4	10	15.0	0.9	10

Supplementary Table 11: : Statistical analysis of body weights from both sexes

ANOVA

	Df	Sum Sq	Mean Sq	F value	Pr(>F)	
Treatment	1	477	477	303.773	< 2e-16	***
Gender	1	25853	25853	16477.803	< 2e-16	***
Time	11	23	2	1.354	0.1919	
Treatment:Gender	1	245	245	155.947	< 2e-16	***
Treatment:Time	11	79	7	4.584	1.32e-06	***
Gender:Time	11	30	3	1.710	0.0687	.
Treatment:Gender:Time	11	63	6	3.623	6.41e-05	***
Residuals	432	678	2			

Supplementary Table 12: Statistical analysis of body weight between male and female the first day of the diet pipepline

Body weight, g	
Male	Female
21.0	19.5
23.5	17.6
24.8	19.3
25.5	18.9
24.8	18.8
22.1	17.6
22.4	18.5
22.8	18.4
20.4	17.0
24.9	19.2
24.3	19.3
24.1	18.1
22.3	18.2
23.5	17.8
22.9	19.3
23.8	17.9
24.0	16.5
23.1	18.3
23.5	18.8
22.6	18.6

**Type of Test**	Unpaired T-test
Data organization	Male vs Female
Number of values	20 vs 20
P value	<0.001
P value summary	***
Significantly different (P < 0.05)?	Yes
One- or two-tailed P value?	Two-tailed
t, df	t=14.39. df=38

**How big is the difference?**	
Mean of column A	23.31
Mean of column M	18.36
Difference between means (M - A) ± SEM	-4.945 ± 0.3435
95% confidence interval	-5.640 to -4.250
R squared (eta squared)	0.845

**F test to compare variances**	
F, DFn, Dfd	2.523. 19. 19
P value	0.05
P value summary	ns
Significantly different (P < 0.05)?	No

**Normality of Residuals**	
Test name: D'Agostino-Pearson omnibus (K2)	
Statistics	2.90
P value	0.23
Passed normality test (alpha=0,05)?	Yes
P value summary	ns

**Descriptive statistics**	Male	Female
Number of values	20	20
Minimum	20.4	16.5
Maximum	25.5	19.5
Range	5.1	3.1
Mean	23.3	18.4
Std. Deviation	1.3	0.8
Std. Error of Mean	0.3	0.2

Supplementary Table 13: Statistical analysis of body weight gain between CD and KD male,and CD and KD female

**Type of Test**	Nonlinear regression: Two phase decay			
**Best-fit values**	CD Male	KD Male	CD Female	KD Female
Y0	23.1	26.8	17.9	19.3
Plateau	11901.0	19.5	197.9	14.9
PercentFast	~ 78.42	37.6	~ 25.10	19.3
KFast	1.0E-05	2.2	4.4E-04	0.6
KSlow	8.3E-06	0.2	4.4E-04	0.2
Half Life (Slow)	8.4E+04	2.8	1.6E+03	3.3
Half Life (Fast)	6.9E+04	0.3	1.6E+03	1.2
Tau (slow)	121085.0	4.1	2264.0	4.7
Tau (fast)	99363.0	0.5	~ 2264	1.7
Rate constant ratio	1.2	9.1	~ 1.000	2.7

Supplementary Table 14: Statistical analysis of body weight loss between CD and KD male,and CD and KD female

		Body weight loss (%)			
Cohort	Mouse n°	CD Male	KD Male	CD Female	KD Female
A	1	6	-21	6	-23
	2	14	-18	8	-9
	3	18	-22	17	-34
B	1	13	-13	-1	-12
	2	9	-17	19	0
	3	19	-17	11	-38
C	1	15	-6	9	-29
	2	11	-16	8	-2
	3	13	-14	14	-14
	4	15	-17	10	-18

Test for normal distribution

**D'Agostino & Pearson test**	CD Male	KD Male	CD Female	KD Female
K2	0.7	6.1	0.7	0.8
P value	0.7	0.1	0.7	0.7
Passed normality test (alpha=0.05)?	Yes	Yes	Yes	Yes
P value summary	ns	ns	ns	ns

**Type of Test**	Welch ANOVA tests
W (DFn, DFd)	91.05 (3.000. 19.26)
P value	<0.001
P value summary	***
Significant diff. among means (P < 0.05)?	Yes

**Dunnett's T3 multiple comparisons test**	**Mean Diff.**	**95.00% CI of diff.**	**Below threshold?**	**Summary**	**Adjusted P Value**
CD Male vs. KD Male	29.51	24.01 to 35.02	Yes	***	<0.001
CD Male vs. CD Female	3.219	-3.238 to 9.676	No	ns	0.61
CD Male vs. KD Female	31.17	17.77 to 44.57	Yes	***	<0.001
KD Male vs. CD Female	-26.29	-33.05 to -19.53	Yes	***	<0.001
KD Male vs. KD Female	1.659	-11.94 to 15.26	No	ns	>0.99
CD Female vs. KD Female	27.95	14.14 to 41.77	Yes	***	<0.001

**Test details**	**Mean 1**	**Mean 2**	**Mean Diff.**	**SE of diff.**	**n1**	**n2**	**t**	**DF**
CD Male vs. KD Male	13.33	-16.18	29.51	1.88	10	10	15.7	17.6
CD Male vs. CD Female	13.33	10.11	3.219	2.175	10	10	1.48	15.88
CD Male vs. KD Female	13.33	-17.84	31.17	4.266	10	10	7.307	10.61
KD Male vs. CD Female	-16.18	10.11	-26.29	2.294	10	10	11.46	17.12
KD Male vs. KD Female	-16.18	-17.84	1.659	4.328	10	10	0.3833	11.16
CD Female vs. KD Female	10.11	-17.84	27.95	4.464	10	10	6.262	12.35

**Descriptive statistics**	CD Male	KD Male	CD Female	KD Female
Number of values	10	10	10	10
Mean	13.3	-16.2	10.1	-17.8
Std. Deviation	3.9	4.5	5.7	12.9
Std. Error of Mean	1.2	1.4	1.8	4.1

Supplementary Table 15: Statistical analysis of male's glycemia datasets

	Glycemia / Mouse (mM)																			
	CD Male										KD Male									
Cohort	A			B			C				A			B			C			
Mouse n°	1	2	3	1	2	3	1	2	3	4	1	2	3	1	2	3	1	2	3	4
Day 1	12.4	11.2	10.6	11.2	9.4	12.7	7.9	8.8	11.7	10.1	9.2	9.7	8.5	8.6	8.6	14.6	9.7	12.2	10.8	10.4
3	11.8	13.7	12.9	15.9	18.3	9.9	9.7	8.7	10.3	10.4	5.1	5.9	4.3	5.6	5.2	5.8	4.6	3.5	4.6	3.9
5	13.2	16.0	12.8	10.9	11.1	10.6	12.1	10.1	9.1	12.1	5.4	7.2	4.8	6.3	5.8	5.2	4.2	10.2	5.6	5.9
8	10.5	12.2	12.6	8.8	9.1	9.8	10.3	10.9	10.2	10.6	3.8	4.6	3.6	6.7	4.3	5.1	6.0	5.8	5.0	6.2
10	10.0	11.3	11.4	9.7	8.8	9.9	7.1	7.6	9.4	10.7	3.7	4.6	3.8	4.9	4.9	3.9	6.1	4.6	4.8	6.3
12	9.3	12.2	11.2	8.3	9.4	9.6	9.3	8.7	8.6	11.2	5.1	4.4	3.4	6.4	4.3	5.2	6.5	4.5	4.2	5.8
15	9.3	10.1	13.7	13.6	11.1	12.7	11.8	9.9	12.1	13.9	4.4	4.4	4.3	5.5	4.6	5.5	6.8	4.3	4.5	6.1
17	10.4	10.7	10.0	8.8	9.8	10.1	7.9	8.2	10.2	10.8	3.5	4.6	3.7	4.5	4.1	4.1	7.0	4.8	4.7	6.4
19	7.7	9.3	9.7	16.2	9.5	9.4	9.6	9.2	9.3	10.3	4.4	3.6	2.7	5.3	4.3	4.9	7.0	4.8	4.5	4.8
22	9.7	8.8	11.3	11.6	11.1	10.6	9.2	10.2	10.3	12.0	4.2	3.5	3.1	4.4	4.6	4.6	6.0	4.7	6.6	5.0
24	7.3	10.7	12.3	10.7	8.2	12.5	10.1	8.4	11.2	11.6	4.4	4.1	3.3	3.9	3.8	4.3	6.3	5.8	7.9	6.2
26	9.7	9.1	10.8	9.8	9.3	10.6	11.2	11.8	10.4	9.9	4.4	4.8	3.8	4.5	4.3	5.1	5.8	5.2	6.7	4.9

Univariate Type III Repeated-Measures ANOVA Assuming Sphericity

Sum Sq num Df Error SS den Df F value Pr(>F)
(Intercept) 15363.2 1 108.87 18 2540.0406 < 2.2e-16 ***
Treatment 1600.6 1 108.87 18 264.6372 3.292e-12 ***
Time 195.4 11 359.33 198 9.7892 4.373e-14 ***
Treatment:Time 156.9 11 359.33 198 7.8596 2.856e-11 ***

Signif. codes: 0 '***' 0.001 '**' 0.01 '*' 0.05 '.' 0.1 ' ' 1

Mauchly Tests for Sphericity

Test statistic p-value
Time 0.0002874 0.00028472
Treatment:Time 0.0002874 0.00028472

Greenhouse-Geisser and Huynh-Feldt Corrections
for Departure from Sphericity

GG eps Pr(>F[GG])
Time 0.44949 1.971e-07 ***
Treatment:Time 0.44949 3.956e-06 ***

Signif. codes: 0 '***' 0.001 '**' 0.01 '*' 0.05 '.' 0.1 ' ' 1

HF eps Pr(>F[HF])
Time 0.6402206 9.468063e-10
Treatment:Time 0.6402206 6.359686e-08

Normal distribution: D'Agostino & Pearson test

**Control Diet datasets**	CD 1	CD 3	CD 5	CD 8	CD 10	CD 12	CD 15	CD 17	CD 19	CD 22	CD 24	CD 26
K2	0.5	2.3	3.5	0.6	0.7	1.6	1.9	1.8	22.3	0.6	1.2	0.5
P value	0.8	0.3	0.2	0.8	0.7	0.4	0.4	0.4	<0.001	0.8	0.6	0.8
Passed normality test (alpha=0.05)?	Yes	Yes	Yes	Yes	Yes	Yes	Yes	Yes	No	Yes	Yes	Yes
P value summary	ns	ns	ns	ns	ns	ns	ns	ns	***	ns	ns	ns
Number of values	10	10	10	10	10	10	10	10	10	10	10	10

**Ketogenic Diet datasets**	KD 1	KD 3	KD 5	KD 8	KD 10	KD 12	KD 15	KD 17	KD 19	KD 22	KD 24	KD 26
K2	6.7	0.7	12.4	1.0	1.0	0.5	2.4	3.7	3.1	0.7	1.6	3.1
P value	0.0	0.7	0.0	0.6	0.6	0.8	0.3	0.2	0.2	0.7	0.4	0.2
Passed normality test (alpha=0.05)?	No	Yes	No	Yes	Yes	Yes	Yes	Yes	Yes	Yes	Yes	Yes
P value summary	*	ns	**	ns	ns	ns	ns	ns	ns	ns	ns	ns
Number of values	10	10	10	10	10	10	10	10	10	10	10	10

**Type of Test**	Mann-Whitney T-test	Welch's T-test	Mann-Whitney T-test	Unpaired T-test	Unpaired T-test	Unpaired T-test	Unpaired T-test	Unpaired T-test	Mann-Whitney T-test	Unpaired T-test	Unpaired T-test	Unpaired T-test
Data organization	CD 1 vs KD 1	CD 3 vs KD 3	CD 5 vs KD 5	CD 8 vs KD 8	CD 10 vs KD 10	CD 12 vs KD 12	CD 15 vs KD 15	CD 17 vs KD 17	CD 19 vs KD 19	CD 22 vs KD 22	CD 24 vs KD 24	CD 26 vs KD 26
Number of values	10 vs 10	10 vs 10	10 vs 10	10 vs 10	10 vs 10	10 vs 10	10 vs 10	10 vs 10	10 vs 10	10 vs 10	10 vs 10	10 vs 10
P value	0.38	<0.001	<0.001	<0.001	<0.001	<0.001	<0.001	<0.001	<0.001	<0.001	<0.001	<0.001
P value summary	ns	***	***	***	***	***	***	***	***	***	***	***
Significantly different (P < 0.05)?	No	Yes	Yes	Yes	Yes	Yes	Yes	Yes	Yes	Yes	Yes	Yes
One- or two-tailed P value?	Two-tailed	Two-tailed	Two-tailed	Two-tailed	Two-tailed	Two-tailed	Two-tailed	Two-tailed	Two-tailed	Two-tailed	Two-tailed	Two-tailed
t, df or *Sum of ranks in column I,U*	*117 , 93*	t=7.297, df=10.25	*153 , 57*	t=10.70, df=18	t=9.061, df=18	t=9.205, df=18	t=11.29, df=18	t=10.27, df=18	*155 , 55*	t=12.46, df=18	t=7.250, df=18	t=14.11, df=18

**How big is the difference?**												
Mean of column A or *Median of column I*	*10.90, n=10*	12.16	*11.60, n=10*	10.5	9.59	9.78	11.82	9.69	*9.450, n=10*	10.48	10.3	10.26
Mean of column M or *Median of column U*	*9.700, n=10*	4.85	*5.700, n=10*	5.11	4.76	4.98	5.04	4.74	*4.650, n=10*	4.67	5	4.95
Difference between means (M - A) ± SEM or *Difference: Actual*	*-1.2*	-7.310 ± 1.002	*-5.9*	-5.390 ± 0.5039	-4.830 ± 0.5331	-4.800 ± 0.5215	-6.780 ± 0.6004	-4.950 ± 0.4818	*-4.8*	-5.810 ± 0.4662	-5.300 ± 0.7311	-5.310 ± 0.3764
95% confidence interval or *Difference: Hodges-Lehmann*	*-0.8*	-9.535 to -5.085	*-5.8*	-6.449 to -4.331	-5.950 to -3.710	-5.896 to -3.704	-8.041 to -5.519	-5.962 to -3.938	*-4.9*	-6.789 to -4.831	-6.836 to -3.764	-6.101 to -4.519
R squared (eta squared)		0.8385		0.8641	0.8202	0.8248	0.8763	0.8543		0.8962	0.7449	0.9171

**F test to compare variances**												
F, DFn, Dfd	1.526, 9, 9	14.30, 9, 9	1.347, 9, 9	1.306, 9, 9	2.630, 9, 9	1.661, 9, 9	3.605, 9, 9	1.197, 9, 9	4.148, 9, 9	1.008, 9, 9	1.482, 9, 9	1.075, 9, 9
P value	0.54	<0.001	0.66	0.7	0.17	0.46	0.07	0.79	0.05	>0.99	0.57	0.92
P value summary	ns	***	ns	ns	ns	ns	ns	ns	*	ns	ns	ns
Significantly different (P < 0.05)?	No	Yes	No	No	No	No	No	No	Yes	No	No	No

**Descriptive statistics**	CD Male			KD Male		
Days	Mean	SEM	N	Mean	SEM	N
1	10.6	0.5	10	10.2	0.6	10
3	12.2	1.0	10	4.9	0.3	10
5	11.8	0.6	10	6.1	0.5	10
8	10.5	0.4	10	5.1	0.3	10
10	9.6	0.5	10	4.8	0.3	10
12	9.8	0.4	10	5.0	0.3	10
15	11.8	0.5	10	5.0	0.3	10
17	9.7	0.3	10	4.7	0.4	10
19	10.0	0.7	10	4.6	0.4	10
22	10.5	0.3	10	4.7	0.3	10
24	10.3	0.6	10	5.0	0.5	10
26	10.3	0.3	10	5.0	0.3	10

Supplementary Table 16: Statistical analysis of female's glycemia datasets

	Glycemia / Mouse (mM)																				
		CD Female										KD Female									
	Cohort	A			B			C				A			B			C			
	Mouse n°	1	2	3	1	2	3	1	2	3	4	1	2	3	1	2	3	1	2	3	4
Day	1	8.5	9.1	7.3	11.8	9.1	10.1	7.4	7.4	6.4	7.5	7.4	8.3	7.6	9.1	5.9	7.4	6.4	6.3	6.8	7.9
	3	10.3	7.7	9.6	6.4	8.7	8.3	9.1	9.5	10.3	10.9	3.6	4.7	3.4	4.3	5.8	4.6	2.8	4.2	4.1	7.0
	5	10.8	8.9	10.1	11.4	7.1	9.6	8.3	8.7	9.1	9.2	3.6	6.2	4.0	5.4	4.2	4.0	3.7	4.7	4.7	5.1
	8	9.8	6.6	9.6	5.5	7.9	8.2	7.8	8.4	8.2	8.3	3.9	4.9	4.2	4.6	6.4	4.0	4.5	4.7	5.1	5.0
	10	9.4	8.7	8.8	5.8	8.3	9.1	8.9	8.3	9.2	9.3	3.4	4.4	3.2	4.2	5.4	3.7	3.3	5.8	4.8	5.4
	12	8.1	6.9	8.7	5.9	7.8	8.0	6.4	8.4	8.4	6.3	3.1	4.2	3.4	3.9	7.6	3.6	3.3	4.0	4.6	3.4
	15	10.1	10.6	9.0	4.4	9.1	8.8	7.4	8.4	6.3	8.0	3.0	4.0	3.7	4.3	7.4	3.1	2.8	4.6	3.6	3.8
	17	7.9	8.2	8.8	5.7	7.4	9.6	8.4	8.4	7.2	8.8	4.4	4.6	3.2	4.8	6.7	2.9	3.5	6.3	4.8	5.4
	19	8.2	7.1	8.3	4.8	9.2	10.3	7.5	9.7	8.3	8.7	3.6	4.6	3.0	4.8	6.0	2.6	3.4	7.1	5.8	5.0
	22	10.1	9.1	12.1	5.6	12.6	10.3	7.4	9.4	8.0	9.4	4.4	5.4	3.9	4.4	6.2	3.0	3.2	6.7	5.1	4.9
	24	8.0	7.8	10.4	7.4	11.8	11.1	8.7	9.7	9.1	8.7	3.4	5.2	2.9	5.5	8.1	3.2	3.8	8.4	5.1	4.7
	26	9.6	7.6	9.4	5.4	9.2	10.7	9.2	10.1	10.7	9.3	3.0	4.1	3.4	5.0	6.8	3.0	4.1	8.1	6.5	5.7

Univariate Type III Repeated-Measures ANOVA Assuming Sphericity

```
Sum Sq num Df Error SS den Df   F value    Pr(>F)
(Intercept)    10764.2      1   172.25     18 1124.8724 < 2.2e-16 ***
Treatment        869.8      1   172.25     18   90.8975 1.852e-08 ***
Time              68.6     11   236.40    198    5.2253 3.247e-07 ***
Treatment:Time    53.4     11   236.40    198    4.0656 2.210e-05 ***
---
Signif. codes:  0 '***' 0.001 '**' 0.01 '*' 0.05 '.' 0.1 ' ' 1
```

Mauchly Tests for Sphericity

```
Test statistic    p-value
Time              2.1625e-05 2.3784e-08
Treatment:Time    2.1625e-05 2.3784e-08
```

Greenhouse-Geisser and Huynh-Feldt Corrections
for Departure from Sphericity

```
GG eps Pr(>F[GG])
Time           0.33793   0.001313 **
Treatment:Time 0.33793   0.006272 **
---
Signif. codes:  0 '***' 0.001 '**' 0.01 '*' 0.05 '.' 0.1 ' ' 1

HF eps   Pr(>F[HF])
Time           0.4368042 0.0003691081
Treatment:Time 0.4368042 0.0026320438
```

Normal distribution: D'Agostino & Pearson test

**Control Diet datasets**	CD 1	CD 3	CD 5	CD 8	CD 10	CD 12	CD 15	CD 17	CD 19	CD 22	CD 24	CD 26
K2	2.4	1.2	0.2	1.6	19.1	2.7	2.7	3.7	4.5	0.2	0.9	8.8
P value	0.3	0.5	0.9	0.4	<0.001	0.3	0.3	0.2	0.1	0.9	0.6	0.01
Passed normality test (alpha=0.05)?	Yes	Yes	Yes	Yes	No	Yes	Yes	Yes	Yes	Yes	Yes	No
P value summary	ns	ns	ns	ns	***	ns	ns	ns	ns	ns	ns	*
Number of values	10	10	10	10	10	10	10	10	10	10	10	10

**Ketogenic Diet datasets**	KD 1	KD 3	KD 5	KD 8	KD 10	KD 12	KD 15	KD 17	KD 19	KD 22	KD 24	KD 26
K2	0.2	3.2	1.5	7.2	2.3	19.8	15.3	0.3	0.5	0.2	1.7	1.0
P value	0.9	0.2	0.5	0.0	0.3	<0.001	<0.001	0.9	0.8	0.9	0.4	0.6
Passed normality test (alpha=0.05)?	Yes	Yes	Yes	No	Yes	No	No	Yes	Yes	Yes	Yes	Yes
P value summary	ns	ns	ns	*	ns	***	***	ns	ns	ns	ns	ns
Number of values	10	10	10	10	10	10	10	10	10	10	10	10

**Type of Test**	Unpaired T-test	Unpaired T-test	Unpaired T-test	Mann-Whitney T-test	Mann-Whitney T-test	Mann-Whitney T-test	Mann-Whitney T-test	Unpaired T-test	Unpaired T-test	Unpaired T-test	Unpaired T-test	Mann-Whitney T-test
Data organization	CD 1 vs KD 1	CD 3 vs KD 3	CD 5 vs KD 5	CD 8 vs KD 8	CD 10 vs KD 10	CD 12 vs KD 12	CD 15 vs KD 15	CD 17 vs KD 17	CD 19 vs KD 19	CD 22 vs KD 22	CD 24 vs KD 24	CD 26 vs KD 26
Number of values	10 vs 10	10 vs 10	10 vs 10	10 vs 10	10 vs 10	10 vs 10	10 vs 10	10 vs 10	10 vs 10	10 vs 10	10 vs 10	10 vs 10
P value	0.07	<0.001	<0.001	<0.001	<0.001	<0.001	<0.001	<0.001	<0.001	<0.001	<0.001	<0.001
P value summary	ns	***	***	***	***	***	***	***	***	***	***	***
Significantly different (P < 0.05)?	No	Yes	Yes	Yes	Yes	Yes	Yes	Yes	Yes	Yes	Yes	Yes
One- or two-tailed P value?	Two-tailed	Two-tailed	Two-tailed	Two-tailed	Two-tailed	Two-tailed	Two-tailed	Two-tailed	Two-tailed	Two-tailed	Two-tailed	Two-tailed
t, df or *Sum of ranks in column I,U*	t=1.924, df=18	t=8.054, df=18	t=10.11, df=18	*154 , 56*	*154.5 , 55.50*	*151 , 59*	*151.5 , 58.50*	t=6.469, df=18	t=5.427, df=18	t=6.144, df=18	t=5.559, df=18	*150 , 60*

**How big is the difference?**												
Mean of column A or *Median of column I*	8.46	9.08	9.32	*8.200, n=10*	*8.850, n=10*	*7.900, n=10*	*8.600, n=10*	8.04	8.21	9.4	9.27	*9.350, n=10*
Mean of column M or *Median of column U*	7.31	4.45	4.56	*4.650, n=10*	*4.300, n=10*	*3.750, n=10*	*3.750, n=10*	4.66	4.59	4.72	5.03	*4.550, n=10*
Difference between means (M - A) ± SEM or *Difference: Actual*	-1.150 ± 0.5977	-4.630 ± 0.5749	-4.760 ± 0.4707	*-3.55*	*-4.55*	*-4.15*	*-4.85*	-3.380 ± 0.5225	-3.620 ± 0.6670	-4.680 ± 0.7617	-4.240 ± 0.7628	*-4.8*
95% confidence interval or *Difference: Hodges-Lehmann*	-2.406 to 0.1057	-5.838 to -3.422	-5.749 to -3.771	*-3.4*	*-4.45*	*-3.8*	*-4.6*	-4.478 to -2.282	-5.021 to -2.219	-6.280 to -3.080	-5.842 to -2.638	*-4.35*
R squared (eta squared)	0.1706	0.7828	0.8503					0.6993	0.6207	0.6771	0.6319	

**F test to compare variances**												
F, DFn, Dfd	2.683, 9, 9	1.259, 9, 9	2.245, 9, 9	3.126, 9, 9	1.195, 9, 9	1.654, 9, 9	1.927, 9, 9	1.343, 9, 9	1.124, 9, 9	3.049, 9, 9	1.733, 9, 9	1.236, 9, 9
P value	0.16	0.74	0.24	0.1	0.8	0.47	0.34	0.67	0.86	0.11	0.43	0.76
P value summary	ns	ns	ns	ns	ns	ns	ns	ns	ns	ns	ns	ns
Significantly different (P < 0.05)?	No	No	No	No	No	No	No	No	No	No	No	No

**Descriptive statistics**	CD Female			KD Female		
Days	Mean	SEM	N	Mean	SEM	N
1	8.5	0.5	10	7.3	0.3	10
3	9.1	0.4	10	4.5	0.4	10
5	9.3	0.4	10	4.6	0.3	10
8	8.0	0.4	10	4.7	0.2	10
10	8.6	0.3	10	4.4	0.3	10
12	7.5	0.3	10	4.1	0.4	10
15	8.2	0.6	10	4.0	0.4	10
17	8.0	0.3	10	4.7	0.4	10
19	8.2	0.5	10	4.6	0.5	10
22	9.4	0.7	10	4.7	0.4	10
24	9.3	0.5	10	5.0	0.6	10
26	9.1	0.5	10	5.0	0.6	10

Supplementary Table 17: Statistical analysis of glycemia between male and female the first and last days of the CD diet intervention

Glycemia, mM			
$Male_{D1}$	$Female_{D1}$	$Male_{D26}$	$Female_{D26}$
12.4	8.5	9.7	9.6
11.2	9.1	9.1	7.6
10.6	7.3	10.8	9.4
11.2	11.8	9.8	5.4
9.4	9.1	9.3	9.2
12.7	10.1	10.6	10.7
7.9	7.4	11.2	9.2
8.8	7.4	11.8	10.1
11.7	6.4	10.4	10.7
10.1	7.5	9.9	9.3
9.2	7.4		
9.7	8.3		
8.5	7.6		
8.6	9.1		
8.6	5.9		
14.6	7.4		
9.7	6.4		
12.2	6.3		
10.8	6.8		
10.4	7.9		

**Type of Test**	Kruskal-Wallis test
P value	<0.001
Exact or approximate P value?	Approximate
P value summary	***
Do the medians vary signif. (P < 0.05)?	Yes
Number of groups	4.00
Kruskal-Wallis statistic	24.50

**Dunn's multiple comparisons test**	**Mean rank diff.**	**Significant?**	**Summary**	**Adjusted P Value**
$Male_{D1}$ vs. $Female_{D1}$	24.5	Yes	***	<0.001
$Male_{D1}$ vs. $Male_{D26}$	-1.35	No	ns	>0.99
$Male_{D1}$ vs. $Female_{D26}$	9.7	No	ns	0.91
$Female_{D1}$ vs. $Male_{D26}$	-25.8	Yes	***	<0.001
$Female_{D1}$ vs. $Female_{D26}$	-14.8	No	ns	0.17
$Male_{D26}$ vs. $Female_{D26}$	11.1	No	ns	0.94

**Test details**	**Mean rank 1**	**Mean rank 2**	**Mean rank diff.**	**n1**	**n2**	**Z**
$Male_{D1}$ vs. $Female_{D1}$	40.1	15.6	24.5	20	20	4.4
$Male_{D1}$ vs. $Male_{D26}$	40.1	41.4	-1.4	20	10	0.2
$Male_{D1}$ vs. $Female_{D26}$	40.1	30.4	9.7	20	10	1.4
$Female_{D1}$ vs. $Male_{D26}$	15.6	41.4	-25.8	20	10	3.8
$Female_{D1}$ vs. $Female_{D26}$	15.6	30.4	-14.8	20	10	2.2
$Male_{D26}$ vs. $Female_{D26}$	41.4	30.4	11.1	10	10	1.4

**Normality of Residuals**	$Male_{D1}$	$Female_{D1}$	$Male_{D26}$	$Female_{D26}$
Test name: D'Agostino-Pearson omnibus (K2)				
K2	2.1	6.9	0.5	8.8
P value	0.4	0.0	0.8	0.01
Passed normality test (alpha=0.05)?	Yes	No	Yes	No
P value summary	ns	*	ns	*

**Descriptive statistics**	$Male_{D1}$	$Female_{D1}$	$Male_{D26}$	$Female_{D26}$
Number of values	20	20	10	10
Minimum	7.9	5.9	9.1	5.4
Maximum	14.6	11.8	11.8	10.7
Range	6.7	5.9	2.7	5.3
Mean	10.4	7.9	10.3	9.1
Std. Deviation	1.7	1.4	0.9	1.6
Std. Error of Mean	0.4	0.3	0.3	0.5

Supplementary Table 18: Statistical analysis of glycemia rate between CD and KD male,and CD and KD female

Type of Test	Nonlinear regression: Two phase decay			
**Best-fit values**	CD Male	KD Male	CD Female	KD Female
Y0	11.6	490.9	8.4	37.8
Plateau	9.9	4.7	180.9	4.6
PercentFast	Unstable	99.83	7.1	Unstable
KFast	0.1	4.6	8.4E-05	2.5
KSlow	4.9E-32	0.1	8.4E-05	2.5
Half Life (Slow)	1.4E+31	7.4	8.3E+03	0.3
Half Life (Fast)	6.4	0.1	8.3E+03	0.3
Tau (slow)	~ 2.028e+031	10.7	11904.0	0.4
Tau (fast)	9.2	0.2	~ 11904	0.4
Rate constant ratio	~ 2.198e+030	49.6	~ 1.000	1.0

Supplementary Table 19: Statistical analysis of glycemia variation between CD and KD male,and CD and KD female

		Glycemia change (%)			
Cohort	Mouse n°	CD Male	KD Male	CD Female	KD Female
A	1	-22.0	-52.0	13.0	-59.0
	2	-19.0	-51.0	-16.0	-51.0
	3	2.0	-55.0	29.0	-55.0
B	1	-13.0	-48.0	-54.0	-45.0
	2	-1.0	-50.0	1.0	15.0
	3	-17.0	-65.0	6.0	-59.0
C	1	41.8	-40.2	24.3	-35.9
	2	34.1	-57.4	36.5	28.6
	3	-11.1	-38.0	67.2	-4.4
	4	-2.0	-52.9	24.0	-27.9

Test for normal distribution

**D'Agostino & Pearson test**	CD Male	KD Male	CD Female	KD Female
K2	3.7	0.3	2.2	1.9
P value	0.2	0.9	0.3	0.4
Passed normality test (alpha=0.05)?	Yes	Yes	Yes	Yes
P value summary	ns	ns	ns	ns

**Type of Test**	Welch ANOVA tests
W (DFn, DFd)	24.30 (3.000, 16.93)
P value	<0.0001
P value summary	****
Significant diff. among means (P < 0.05)?	Yes

**Dunnett's T3 multiple comparisons test**	**Mean Diff.**	**95.00% CI of diff.**	**Below threshold?**	**Summary**	**Adjusted P Value**
CD Male vs. KD Male	50.2	27.06 to 73.38	Yes	***	0.0
CD Male vs. CD Female	-13.8	-50.66 to 23.02	No	ns	0.8
CD Male vs. KD Female	28.6	-7.669 to 64.95	No	ns	0.2
KD Male vs. CD Female	-64.0	-97.88 to -30.20	Yes	***	0.0
KD Male vs. KD Female	-21.6	-54.75 to 11.59	No	ns	0.3
CD Female vs. KD Female	42.5	0.3134 to 84.61	Yes	*	0.0

**Test details**	**Mean 1**	**Mean 2**	**Mean Diff.**	**SE of diff.**	**n1**	**n2**	**t**	**DF**
CD Male vs. KD Male	-0.7	-50.9	50.2	7.4	10	10	6.8	11.3
CD Male vs. CD Female	-0.7	13.1	-13.8	12.4	10	10	1.1	15.8
CD Male vs. KD Female	-0.7	-29.4	28.6	12.2	10	10	2.3	16.0
KD Male vs. CD Female	-50.9	13.1	-64.0	10.6	10	10	6.1	10.0
KD Male vs. KD Female	-50.9	-29.4	-21.6	10.4	10	10	2.1	10.1
CD Female vs. KD Female	13.1	-29.4	42.5	14.4	10	10	3.0	18.0

**Descriptive statistics**	CD Male	KD Male	CD Female	KD Female
Number of values	10	10	10	10
Mean	-0.7	-50.9	13.1	-29.4
Std. Deviation	22.0	7.8	32.5	31.8
Std. Error of Mean	6.9	2.5	10.3	10.1

Supplementary Table 20: Statistical analysis of male's blood β-HB datasets

	Blood β-HB / Mouse (mM)																				
	CD Male										KD Male										
Cohort	A			B			C				A			B			C				
Mouse n°	1	2	3	1	2	3	1	2	3	4	1	2	3	1	2	3	1	2	3	4
Day 1	0.6	0.4	0.3	0.3	0.4	0.4	0.4	0.4	0.9	0.3	0.5	0.4	0.6	0.3	0.3	0.4	0.4	0.9	0.3	0.2
3	0.4	0.4	0.3	0.6	0.9	0.5	0.4	0.3	0.4	0.4	5.2	2.7	1.8	2.2	3.2	4.5	4.9	4.6	4.1	5.4
5	0.5	0.3	0.5	0.4	0.4	0.3	0.3	0.3	0.4	0.4	5.1	3.8	2.2	3.5	1.9	2.9	5.7	2.9	4.6	4.1
8	0.3	0.6	0.4	0.6	0.6	0.4	0.4	0.4	0.6	0.3	5.1	3.0	2.2	3.7	3.3	4.4	3.0	3.9	4.7	3.6
10	0.4	0.3	0.4	0.5	0.8	0.3	0.5	0.3	0.6	0.4	6.4	4.1	2.7	3.4	3.9	5.2	2.4	3.9	5.9	3.3
12	0.4	0.3	0.3	0.6	0.3	0.4	0.2	0.3	0.4	0.3	5.1	4.4	2.3	3.2	5.6	5.4	2.6	4.2	5.4	3.9
15	0.4	0.3	0.3	0.7	0.4	0.4	0.3	0.4	0.4	0.3	2.8	5.8	2.6	3.2	3.9	3.2	1.9	4.1	2.9	3.7
17	0.3	0.3	0.4	0.5	0.5	0.4	0.6	0.4	0.3	0.3	5.1	4.3	4.1	4.4	3.8	4.9	1.8	3.2	4.6	3.8
19	0.6	0.5	0.4	0.6	0.4	0.4	0.3	0.4	0.4	0.3	6.6	3.7	3.3	3.4	2.9	3.7	2.7	3.0	4.3	4.8
22	0.4	0.4	0.3	0.6	0.5	0.4	0.3	0.3	0.3	0.2	5.1	3.8	2.9	3.2	3.9	4.4	1.8	2.7	2.9	4.8
24	0.4	0.3	0.2	0.3	0.3	0.2	0.4	0.4	0.5	0.4	5.0	4.0	3.8	3.3	3.9	3.7	1.7	2.3	2.8	3.1
26	0.4	0.7	0.5	0.4	0.5	0.4	0.3	0.3	0.4	0.4	4.6	3.1	3.2	2.8	4.3	3.2	2.2	2.2	2.2	3.1

```
Univariate Type III Repeated-Measures ANOVA Assuming Sphericity

Sum Sq num Df Error SS den Df F value    Pr(>F)
(Intercept)    886.66      1   42.627     18 374.409 1.703e-13 ***
Treatment      549.95      1   42.627     18 232.226 9.892e-12 ***
Time            54.36     11   78.278    198  12.499 < 2.2e-16 ***
Treatment:Time  56.07     11   78.278    198  12.893 < 2.2e-16 ***
---
Signif. codes:  0 '***' 0.001 '**' 0.01 '*' 0.05 '.' 0.1 ' ' 1


Mauchly Tests for Sphericity

Test statistic    p-value
Time              8.0401e-07 1.1735e-14
Treatment:Time    8.0401e-07 1.1735e-14


Greenhouse-Geisser and Huynh-Feldt Corrections
for Departure from Sphericity

GG eps Pr(>F[GG])
Time           0.38457  4.162e-08 ***
Treatment:Time 0.38457  2.604e-08 ***
---
Signif. codes:  0 '***' 0.001 '**' 0.01 '*' 0.05 '.' 0.1 ' ' 1

HF eps   Pr(>F[HF])
Time           0.5174468 3.246641e-10
Treatment:Time 0.5174468 1.738062e-10
```

Normal distribution: D'Agostino & Pearson test

**Control Diet datasets**	CD 1	CD 3	CD 5	CD 8	CD 10	CD 12	CD 15	CD 17	CD 19	CD 22	CD 24	CD 26
K2	13.9	12.6	1.1	3.9	4.9	6.8	16.2	1.2	1.0	1.5	0.2	7.3
P value	<0.001	0.0	0.6	0.1	0.1	0.0	<0.001	0.6	0.6	0.5	0.9	0.0
Passed normality test (alpha=0.05)?	No	No	Yes	Yes	Yes	No	No	Yes	Yes	Yes	Yes	No
P value summary	***	**	ns	ns	ns	*	***	ns	ns	ns	ns	*
Number of values	10	10	10	10	10	10	10	10	10	10	10	10

**Ketogenic Diet datasets**	KD 1	KD 3	KD 5	KD 8	KD 10	KD 12	KD 15	KD 17	KD 19	KD 22	KD 24	KD 26
K2	8.2	1.9	0.4	0.0	0.9	1.5	5.2	6.9	9.1	0.2	0.2	1.2
P value	0.0	0.4	0.8	>0.99	0.6	0.5	0.1	0.0	0.0	0.9	0.9	0.5
Passed normality test (alpha=0.05)?	No	Yes	Yes	Yes	Yes	Yes	Yes	No	No	Yes	Yes	Yes
P value summary	*	ns	ns	ns	ns	ns	ns	*	*	ns	ns	ns
Number of values	10	10	10	10	10	10	10	10	10	10	10	10

**Type of Test**	Mann-Whitney T-test	Mann-Whitney T-test	Mann-Whitney T-test	Welch's T-test	Welch's T-test	Mann-Whitney T-test	Mann-Whitney T-test	Mann-Whitney T-test	Mann-Whitney T-test	Welch's T-test	Welch's T-test	Mann-Whitney T-test
Data organization	CD 1 vs KD 1	CD 3 vs KD 3	CD 5 vs KD 5	CD 8 vs KD 8	CD 10 vs KD 10	CD 12 vs KD 12	CD 15 vs KD 15	CD 17 vs KD 17	CD 19 vs KD 19	CD 22 vs KD 22	CD 24 vs KD 24	CD 26 vs KD 26
Number of values	10 vs 10	10 vs 10	10 vs 10	10 vs 10	10 vs 10	10 vs 10	10 vs 10	10 vs 10	10 vs 10	10 vs 10	10 vs 10	10 vs 10
P value	0.85	<0.001	<0.001	<0.001	<0.001	<0.001	<0.001	<0.001	<0.001	<0.001	<0.001	<0.001
P value summary	ns	***	***	***	***	***	***	***	***	***	***	***
Significantly different (P < 0.05)?	No	Yes	Yes	Yes	Yes	Yes	Yes	Yes	Yes	Yes	Yes	Yes
One- or two-tailed P value?	Two-tailed	Two-tailed	Two-tailed	Two-tailed	Two-tailed	Two-tailed	Two-tailed	Two-tailed	Two-tailed	Two-tailed	Two-tailed	Two-tailed
t, df or *Sum of ranks in column I,U*	*108 , 102*	*55 , 155*	*55 , 155*	t=11.55, df=9.376	t=8.698, df=9.256	*55 , 155*	*55 , 155*	*55 , 155*	*55 , 155*	t=9.664, df=9.226	t=10.10, df=9.190	*55 , 155*

**How big is the difference?**												
Mean of column A or *Median of column I*	*0.4000, n=10*	*0.4000, n=10*	*0.4000, n=10*	0.46	0.45	*0.3000, n=10*	*0.4000, n=10*	*0.4000, n=10*	*0.4000, n=10*	0.37	0.34	*0.4000, n=10*
Mean of column M or *Median of column U*	*0.4000, n=10*	*4.300, n=10*	*3.650, n=10*	3.69	4.12	*4.300, n=10*	*3.200, n=10*	*4.200, n=10*	*3.550, n=10*	3.55	3.36	*3.100, n=10*
Difference between means (M - A) ± SEM or *Difference: Actual*	*0*	*3.9*	*3.25*	3.230 ± 0.2795	3.670 ± 0.4219	*4*	*2.8*	*3.8*	*3.15*	3.180 ± 0.3291	3.020 ± 0.2990	*2.7*
95% confidence interval or *Difference: Hodges-Lehmann*	*0*	*3.75*	*3.25*	2.601 to 3.859	2.720 to 4.620	*3.95*	*2.8*	*3.8*	*3.1*	2.438 to 3.922	2.346 to 3.694	*2.7*
R squared (eta squared)				0.9344	0.891					0.9101	0.9173	

**F test to compare variances**												
F, DFn, Dfd	1.188, 9, 9	52.97, 9, 9	245.4, 9, 9	47.84, 9, 9	70.20, 9, 9	123.3, 9, 9	78.83, 9, 9	82.00, 9, 9	121.0, 9, 9	79.55, 9, 9	94.81, 9, 9	51.48, 9, 9
P value	0.8	<0.001	<0.001	<0.001	<0.001	<0.001	<0.001	<0.001	<0.001	<0.001	<0.001	<0.001
P value summary	ns	***	***	***	***	***	***	***	***	***	***	***
Significantly different (P < 0.05)?	No	Yes	Yes	Yes	Yes	Yes	Yes	Yes	Yes	Yes	Yes	Yes

**Descriptive statistics**	CD Male			KD Male		
Days	Mean	SEM	N	Mean	SEM	N
1	0.4	0.1	10	0.4	0.1	10
3	0.5	0.1	10	3.9	0.4	10
5	0.4	0.02	10	3.7	0.4	10
8	0.5	0.04	10	3.7	0.3	10
10	0.5	0.1	10	4.1	0.4	10
12	0.4	0.03	10	4.2	0.4	10
15	0.4	0.04	10	3.4	0.3	10
17	0.4	0.03	10	4.0	0.3	10
19	0.4	0.03	10	3.8	0.4	10
22	0.4	0.04	10	3.6	0.3	10
24	0.3	0.03	10	3.4	0.3	10
26	0.4	0.04	10	3.1	0.3	10

Supplementary Table 21: Statistical analysis of female's blood β-HB datasets

		Blood β-HB / Mouse (mM)																			
		CD Female										KD Female									
	Cohort	A			B			C				A			B			C			
	Mouse n°	1	2	3	1	2	3	1	2	3	4	1	2	3	1	2	3	1	2	3	4
Day	1	0.6	0.3	0.4	0.8	0.4	0.5	0.7	0.6	0.8	0.7	0.6	0.7	0.7	0.8	0.8	0.6	0.6	0.5	0.7	0.6
	3	0.5	0.7	0.3	0.7	0.4	0.6	0.4	0.4	0.4	0.3	4.9	6.1	7.1	5.9	3.9	5.0	6.7	6.4	5.9	5.9
	5	0.4	0.3	0.4	0.4	0.7	0.6	0.6	0.5	0.6	0.4	6.4	6.7	7.1	7.9	6.7	8.0	8.0	6.6	6.4	7.1
	8	0.3	0.4	0.2	0.7	0.5	0.6	0.6	0.7	0.6	0.4	6.9	6.3	7.6	7.8	3.0	8.0	8.0	5.5	7.5	7.1
	10	0.4	0.3	0.3	0.6	0.4	0.5	0.4	0.6	0.5	0.2	7.2	7.1	7.5	8.0	3.3	8.0	7.7	6.7	8.0	6.2
	12	0.4	0.6	0.5	0.8	0.6	0.6	0.5	0.4	0.4	0.6	6.9	6.2	8.0	8.0	2.6	8.0	8.0	4.9	7.3	8.0
	15	0.5	0.7	0.8	0.6	0.7	0.4	0.7	0.5	0.4	0.6	7.6	6.2	8.0	7.2	2.6	8.0	7.6	5.0	8.0	6.1
	17	0.4	0.2	0.3	0.6	0.7	0.5	0.4	0.4	0.3	0.4	8.0	6.5	8.0	6.5	2.4	8.0	8.0	3.4	5.6	7.0
	19	0.5	0.5	0.5	0.7	0.4	0.5	0.5	0.3	0.7	0.4	6.4	5.4	7.8	4.7	4.1	8.0	7.3	3.3	5.3	5.8
	22	0.4	0.5	0.5	0.7	0.7	0.6	0.5	0.5	0.5	0.4	7.3	3.4	8.0	6.3	3.3	8.0	8.0	3.5	6.2	5.7
	24	0.6	0.6	0.4	0.6	0.4	0.7	0.3	0.9	0.5	0.4	8.0	3.9	8.0	5.8	2.7	8.0	7.2	2.0	5.7	6.4
	26	0.7	0.6	0.4	0.6	0.5	0.3	0.6	0.6	0.4	0.4	6.3	4.8	8.0	5.5	2.8	7.8	7.7	2.3	6.2	7.3

```
Univariate Type III Repeated-Measures ANOVA Assuming Sphericity

Sum Sq num Df Error SS den Df F value    Pr(>F)
(Intercept)    2440.99      1  186.763     18 235.260 8.871e-12 ***
Treatment      1725.92      1  186.763     18 166.342 1.567e-10 ***
Time            157.33     11   97.879    198  28.932 < 2.2e-16 ***
Treatment:Time  166.28     11   97.879    198  30.579 < 2.2e-16 ***
---
Signif. codes:  0 '***' 0.001 '**' 0.01 '*' 0.05 '.' 0.1 ' ' 1


Mauchly Tests for Sphericity

Test statistic    p-value
Time                3.2727e-08 1.5247e-21
Treatment:Time      3.2727e-08 1.5247e-21


Greenhouse-Geisser and Huynh-Feldt Corrections
for Departure from Sphericity

GG eps Pr(>F[GG])
Time           0.24159  3.062e-10 ***
Treatment:Time 0.24159  1.351e-10 ***
---
Signif. codes:  0 '***' 0.001 '**' 0.01 '*' 0.05 '.' 0.1 ' ' 1

HF eps   Pr(>F[HF])
Time           0.2873315 8.805987e-12
Treatment:Time 0.2873315 3.339487e-12
```

Normal distribution: D'Agostino & Pearson test

**Control Diet datasets**	CD 1	CD 3	CD 5	CD 8	CD 10	CD 12	CD 15	CD 17	CD 19	CD 22	CD 24	CD 26
K2	1.2	1.5	1.1	0.9	0.2	1.5	0.9	1.0	0.5	1.0	1.5	1.1
P value	0.6	0.5	0.6	0.6	0.9	0.5	0.6	0.6	0.8	0.6	0.5	0.6
Passed normality test (alpha=0.05)?	Yes	Yes	Yes	Yes	Yes	Yes	Yes	Yes	Yes	Yes	Yes	Yes
P value summary	ns	ns	ns	ns	ns	ns	ns	ns	ns	ns	ns	ns
Number of values	10	10	10	10	10	10	10	10	10	10	10	10

**Ketogenic Diet datasets**	KD 1	KD 3	KD 5	KD 8	KD 10	KD 12	KD 15	KD 17	KD 19	KD 22	KD 24	KD 26
K2	0.15	1.45	2.54	11.39	16.25	8.69	7.76	3.14	0.54	2.50	1.44	1.52
P value	0.93	0.48	0.28	0.003	<0.001	0.01	0.02	0.21	0.76	0.29	0.49	0.47
Passed normality test (alpha=0.05)?	Yes	Yes	Yes	No	No	No	No	Yes	Yes	Yes	Yes	Yes
P value summary	ns	ns	ns	**	***	*	*	ns	ns	ns	ns	ns
Number of values	10	10	10	10	10	10	10	10	10	10	10	10

**Type of Test**	Unpaired T-test	Welch's T-test	Welch's T-test	Mann-Whitney T-test	Mann-Whitney T-test	Mann-Whitney T-test	Mann-Whitney T-test	Welch's T-test	Welch's T-test	Welch's T-test	Welch's T-test	Welch's T-test
Data organization	CD 1 vs KD 1	CD 3 vs KD 3	CD 5 vs KD 5	CD 8 vs KD 8	CD 10 vs KD 10	CD 12 vs KD 12	CD 15 vs KD 15	CD 17 vs KD 17	CD 19 vs KD 19	CD 22 vs KD 22	CD 24 vs KD 24	CD 26 vs KD 26
Number of values	10 vs 10	10 vs 10	10 vs 10	10 vs 10	10 vs 10	10 vs 10	10 vs 10	10 vs 10	10 vs 10	10 vs 10	10 vs 10	10 vs 10
P value	0.22	<0.001	<0.001	<0.001	<0.001	<0.001	<0.001	<0.001	<0.001	<0.001	<0.001	<0.001
P value summary	ns	***	***	***	***	***	***	***	***	***	***	***
Significantly different (P < 0.05)?	No	Yes	Yes	Yes	Yes	Yes	Yes	Yes	Yes	Yes	Yes	Yes
One- or two-tailed P value?	Two-tailed	Two-tailed	Two-tailed	Two-tailed	Two-tailed	Two-tailed	Two-tailed	Two-tailed	Two-tailed	Two-tailed	Two-tailed	Two-tailed
t, df or *Sum of ranks in column I,U*	t=1.265, df=18	t=17.55, df=9.450	t=31.47, df=9.703	*55 , 155*	*55 , 155*	*55 , 155*	*55 , 155*	t=9.306, df=9.097	t=10.66, df=9.113	t=8.812, df=9.053	t=7.414, df=9.115	t=8.298, df=9.072

**How big is the difference?**												
Mean of column A or *Median of column I*	0.58	0.47	0.49	*0.5500, n=10*	*0.4000, n=10*	*0.5500, n=10*	*0.6000, n=10*	0.42	0.5	0.53	0.54	0.51
Mean of column M or *Median of column U*	0.66	5.78	07.09	*7.300, n=10*	*7.350, n=10*	*7.650, n=10*	*7.400, n=10*	6.34	5.81	5.97	5.77	5.87
Difference between means (M - A) ± SEM or *Difference: Actual*	0.08000 ± 0.06325	5.310 ± 0.3025	6.600 ± 0.2097	*6.75*	*6.95*	*7.1*	*6.8*	5.920 ± 0.6361	5.310 ± 0.4983	5.440 ± 0.6174	5.230 ± 0.7054	5.360 ± 0.6459
95% confidence interval or *Difference: Hodges-Lehmann*	-0.05287 to 0.2129	4.631 to 5.989	6.131 to 7.069	*6.8*	*6.9*	*07.05*	*6.8*	4.483 to 7.357	4.185 to 6.435	4.045 to 6.835	3.637 to 6.823	3.901 to 6.819
R squared (eta squared)	0.08163	0.9702	0.9903					0.9049	0.9257	0.8956	0.8578	0.8836

**F test to compare variances**												
F, DFn, Dfd	3.286, 9, 9	39.98, 9, 9	25.56, 9, 9	82.62, 9, 9	116.7, 9, 9	201.9, 9, 9	161.0, 9, 9	184.8, 9, 9	158.6, 9, 9	338.6, 9, 9	156.7, 9, 9	251.0, 9, 9
P value	0.09	<0.001	<0.001	<0.001	<0.001	<0.001	<0.001	<0.001	<0.001	<0.001	<0.001	<0.001
P value summary	ns	***	***	***	***	***	***	***	***	***	***	***
Significantly different (P < 0.05)?	No	Yes	Yes	Yes	Yes	Yes	Yes	Yes	Yes	Yes	Yes	Yes

**Descriptive statistics**	CD Female			KD Female		
Days	Mean	SEM	N	Mean	SEM	N
1	0.6	0.1	10	0.7	0.03	10
3	0.5	0.05	10	5.78	0.3	10
5	0.5	0.04	10	07.09	0.2	10
8	0.5	0.1	10	6.77	0.5	10
10	0.4	0.04	10	7.0	0.4	10
12	0.5	0.04	10	6.79	0.6	10
15	0.6	0.04	10	6.63	0.5	10
17	0.4	0.05	10	6.34	0.6	10
19	0.5	0.04	10	5.81	0.5	10
22	0.5	0.03	10	6.0	0.6	10
24	0.5	0.1	10	5.77	0.7	10
26	0.5	0.04	10	5.87	0.6	10

Supplementary Table 22: Statistical analysis of blood β-HB between male and female the first and last days of the diet intervention

Blood β-HB, mM			
$Male_{D1}$	$Female_{D1}$	$Male_{D26}$	$Female_{D26}$
0.6	0.6	0.4	0.7
0.4	0.3	0.7	0.6
0.3	0.4	0.5	0.4
0.3	0.8	0.4	0.6
0.4	0.4	0.5	0.5
0.4	0.5	0.4	0.3
0.4	0.7	0.3	0.6
0.4	0.6	0.3	0.6
0.9	0.8	0.4	0.4
0.3	0.7	0.4	0.4
0.5	0.6		
0.4	0.7		
0.6	0.7		
0.3	0.8		
0.3	0.8		
0.4	0.6		
0.4	0.6		
0.9	0.5		
0.3	0.7		
0.2	0.6		

**Type of Test**	Kruskal-Wallis test
P value	0.001
Exact or approximate P value?	Approximate
P value summary	**
Do the medians vary signif. (P < 0.05)?	Yes
Number of groups	4.0
Kruskal-Wallis statistic	16.2

**Dunn's multiple comparisons test**	**Mean rank diff.**	**Significant?**	**Summary**	**Adjusted P Value**
$Male_{D1}$ vs. $Female_{D1}$	-20.2	Yes	**	0.001
$Male_{D1}$ vs. $Male_{D26}$	-1.23	No	ns	>0.99
$Male_{D1}$ vs. $Female_{D26}$	-9.83	No	ns	0.8
$Female_{D1}$ vs. $Male_{D26}$	19	Yes	*	0.02
$Female_{D1}$ vs. $Female_{D26}$	10.4	No	ns	0.7
$Male_{D26}$ vs. $Female_{D26}$	-8.6	No	ns	>0.99

**Test details**	**Mean rank 1**	**Mean rank 2**	**Mean rank diff.**	**n1**	**n2**	**Z**
$Male_{D1}$ vs. $Female_{D1}$	21.9	42.1	-20.2	20	20	3.7
$Male_{D1}$ vs. $Male_{D26}$	21.9	23.2	-1.2	20	10	0.2
$Male_{D1}$ vs. $Female_{D26}$	21.9	31.8	-9.8	20	10	1.5
$Female_{D1}$ vs. $Male_{D26}$	42.1	23.2	19.0	20	10	2.9
$Female_{D1}$ vs. $Female_{D26}$	42.1	31.8	10.4	20	10	1.6
$Male_{D26}$ vs. $Female_{D26}$	23.2	31.8	-8.6	10	10	1.1

**Normality of Residuals**	$Male_{D1}$	$Female_{D1}$	$Male_{D26}$	$Female_{D26}$
Test name: D'Agostino-Pearson omnibus (K2)				
K2	11.9	1.6	7.3	1.1
P value	0.0	0.5	0.0	0.58
Passed normality test (alpha=0.05)?	No	Yes	No	Yes
P value summary	**	ns	*	ns

**Descriptive statistics**	$Male_{D1}$	$Female_{D1}$	$Male_{D26}$	$Female_{D26}$
Number of values	20	20	10	10
Minimum	0.2	0.3	0.3	0.3
Maximum	0.9	0.8	0.7	0.7
Range	0.7	0.5	0.4	0.4
Mean	0.4	0.6	0.4	0.5
Std. Deviation	0.2	0.1	0.1	0.1
Std. Error of Mean	0.04	0.03	0.04	0.04

Supplementary Table 23: Statistical analysis of blood β-HB rate between CD and KD male,and CD and KD female

Type of Test	egression in two phases	
**Best-fit values**	KD Male	KD Female
Y0	-63.4	-13.3
Plateau	3.7	6.3
PercentFast	Unstable	Unstable
KFast	3.0	1.2
KSlow	3.0	1.2
Half Life (Slow)	3.0	0.6
Half Life (Fast)	0.2	0.6
Tau (slow)	0.3	0.8
Tau (fast)	0.3	0.8
Rate constant ratio	1.0	1.0

Type of Test	Simple linear regression	
	CD Male	CD Female
Slope	-2.3E-03	3.6E-04

Supplementary Table 24: Statistical analysis of blood β-HB variation between CD and KD male,and CD and KD female

		Blood β-HB change (%)			
Cohort	Mouse n°	CD Male	KD Male	CD Female	KD Female
A	1	-33	820	17	950
	2	75	675	100	586
	3	67	433	0	1043
B	1	33	833	-25	588
	2	25	1333	13	250
	3	0	700	-25	1200
C	1	-25	450	-14	1183
	2	-25	144	0	360
	3	-56	633	-50	786
	4	33	1450	-43	1117

Test for normal distribution

**D'Agostino & Pearson test**	CD Male	KD Male	CD Female	KD Female
K2	1.0	0.9	9.9	1.7
P value	0.6	0.6	0.01	0.4
Passed normality test (alpha=0.05)?	Yes	Yes	No	Yes
P value summary	ns	ns	**	ns

Type of Test	Kruskal-Wallis test
P value	<0.0001
Exact or approximate P value?	Approximate
P value summary	***
Do the medians vary signif. (P < 0.05)?	Yes
Number of groups	4
Kruskal-Wallis statistic	29.45

**Dunn's multiple comparisons test**	Mean rank diff.	Significant?	Summary	Adjusted P Value
CD Male vs. KD Male	-18.7	Yes	**	0.002
CD Male vs. CD Female	1.8	No	ns	>0.99
CD Male vs. KD Female	-19.5	Yes	**	0.001
KD Male vs. CD Female	20.5	Yes	***	<0.001
KD Male vs. KD Female	-0.8	No	ns	>0.99
CD Female vs. KD Female	-21.3	Yes	***	<0.001

**Test details**	Mean rank 1	Mean rank 2	Mean rank diff.	n1	n2	Z
CD Male vs. KD Male	11.4	30.1	-18.7	10	10	3.58
CD Male vs. CD Female	11.4	9.6	1.8	10	10	0.345
CD Male vs. KD Female	11.4	30.9	-19.5	10	10	3.73
KD Male vs. CD Female	30.1	9.6	20.5	10	10	3.92
KD Male vs. KD Female	30.1	30.9	-0.8	10	10	0.153
CD Female vs. KD Female	9.6	30.9	-21.3	10	10	04.08

**Descriptive statistics**	CD Male	KD Male	CD Female	KD Female
Number of values	10	10	10	10
Mean	9.5	747.2	-2.7	806.3
Std. Deviation	44.1	397.3	42.4	345.7
Std. Error of Mean	13.9	125.6	13.4	109.3

Supplementary Table 25: Statistical analysis of male's blood lactate datasets

	Blood lacate / Mouse (mM)																			
	CD Male										KD Male									
Cohort	A			B			C				A			B			C			
Mouse n°	1	2	3	1	2	3	1	2	3	4	1	2	3	1	2	3	1	2	3	4
Day 1	4.6	2.8	2.3	2.8	3.1	2.7	2.2	2.6	3.1	2.1	4.9	3.0	1.9	2.4	2.7	2.8	2.4	3.7	2.9	2.1
3	4.0	4.1	4.7	4.4	6.4	2.6	2.1	3.4	3.5	2.4	4.1	2.7	3.8	2.6	2.6	2.0	1.8	3.1	2.2	1.8
5	3.6	4.6	4.4	4.9	3.2	2.7	2.8	4.1	3.6	3.7	4.9	3.1	2.8	2.5	2.1	2.0	2.3	4.1	3.7	3.8
8	3.7	4.3	2.4	2.9	2.2	2.7	2.3	2.5	2.0	3.3	3.2	2.4	2.4	2.6	2.0	1.7	1.7	2.8	2.3	3.0
10	3.8	3.6	2.7	3.3	4.4	2.5	2.1	2.8	2.9	2.7	2.5	2.5	2.4	2.2	2.1	2.0	2.0	2.6	2.3	2.5
12	2.7	2.8	2.3	3.7	2.8	2.3	3.3	2.8	3.1	3.1	2.5	2.3	2.3	3.5	2.4	2.0	2.8	2.1	2.6	2.2
15	5.0	2.9	2.6	4.4	3.9	2.3	2.7	3.0	2.3	2.6	2.8	1.9	1.8	2.4	3.0	1.8	2.5	3.1	2.7	2.0
17	3.2	3.2	2.7	1.9	2.2	2.8	2.8	2.6	2.2	2.6	2.0	2.0	1.9	1.9	2.2	1.7	2.7	2.3	2.0	2.0
19	2.5	2.2	2.6	4.2	3.8	2.9	2.1	2.3	2.6	2.5	2.9	1.3	1.6	2.3	2.6	1.5	2.1	2.3	2.7	2.0
22	3.5	3.4	2.7	3.9	4.2	4.9	2.0	2.6	2.0	3.1	3.0	2.9	1.8	3.5	1.8	2.0	2.2	2.2	2.4	2.4
24	2.2	3.0	2.5	4.2	4.5	4.3	2.4	3.3	2.2	2.5	4.0	2.9	2.2	5.3	3.1	2.8	2.8	3.1	3.7	2.5
26	3.5	3.8	2.3	2.5	3.0	3.6	2.8	3.3	4.3	4.1	2.2	1.9	1.9	3.2	2.6	2.2	2.7	2.7	2.6	2.5

```
Univariate Type III Repeated-Measures ANOVA Assuming Sphericity

Sum Sq num Df Error SS den Df   F value    Pr(>F)
(Intercept)    1923.83      1   30.835     18 1123.0517 < 2.2e-16 ***
Treatment        19.21      1   30.835     18   11.2140  0.003575 **
Time            22.31     11   79.668    198    5.0411 6.337e-07 ***
Treatment:Time    7.13     11   79.668    198    1.6110  0.097843 .
---
Signif. codes:  0 '***' 0.001 '**' 0.01 '*' 0.05 '.' 0.1 ' ' 1


Mauchly Tests for Sphericity

Test statistic   p-value
Time               0.00071507 0.0041277
Treatment:Time     0.00071507 0.0041277


Greenhouse-Geisser and Huynh-Feldt Corrections
for Departure from Sphericity

GG eps Pr(>F[GG])
Time           0.49501   0.000249 ***
Treatment:Time 0.49501   0.158672
---
Signif. codes:  0 '***' 0.001 '**' 0.01 '*' 0.05 '.' 0.1 ' ' 1

HF eps   Pr(>F[HF])
Time           0.7346382 1.432532e-05
Treatment:Time 0.7346382 1.255735e-01
```

Normal distribution: D'Agostino & Pearson test

**Control Diet datasets**	CD 1	CD 3	CD 5	CD 8	CD 10	CD 12	CD 15	CD 17	CD 19	CD 22	CD 24	CD 26
K2	12.2	1.8	0.6	2.4	1.0	0.4	2.7	0.2	4.9	0.3	2.7	0.7
P value	0.002	0.4	0.7	0.3	0.6	0.8	0.3	0.9	0.1	0.9	0.3	0.7
Passed normality test (alpha=0.05)?	No	Yes	Yes	Yes	Yes	Yes	Yes	Yes	Yes	Yes	Yes	Yes
P value summary	**	ns	ns	ns	ns	ns	ns	ns	ns	ns	ns	ns
Number of values	10	10	10	10	10	10	10	10	10	10	10	10

**Ketogenic Diet datasets**	KD 1	KD 3	KD 5	KD 8	KD 10	KD 12	KD 15	KD 17	KD 19	KD 22	KD 24	KD 26
K2	7.6	1.3	0.9	0.4	2.4	9.1	2.8	6.6	0.9	1.4	7.0	0.1
P value	0.02	0.5	0.7	0.8	0.3	0.0	0.2	0.0	0.7	0.5	0.0	1.0
Passed normality test (alpha=0.05)?	No	Yes	Yes	Yes	Yes	No	Yes	No	Yes	Yes	No	Yes
P value summary	*	ns	ns	ns	ns	*	ns	*	ns	ns	*	ns
Number of values	10	10	10	10	10	10	10	10	10	10	10	10

**Type of Test**	Mann-Whitney T-test	Unpaired T-test	Unpaired T-test	Unpaired T-test	Welch's T-test	Mann-Whitney T-test	Unpaired T-test	Mann-Whitney T-test	Unpaired T-test	Unpaired T-test	Mann-Whitney T-test	Unpaired T-test
Data organization	CD 1 vs KD 1	CD 3 vs KD 3	CD 5 vs KD 5	CD 8 vs KD 8	CD 10 vs KD 10	CD 12 vs KD 12	CD 15 vs KD 15	CD 17 vs KD 17	CD 19 vs KD 19	CD 22 vs KD 22	CD 24 vs KD 24	CD 26 vs KD 26
Number of values	10 vs 10	10 vs 10	10 vs 10	10 vs 10	10 vs 10	10 vs 10	10 vs 10	10 vs 10	10 vs 10	10 vs 10	10 vs 10	10 vs 10
P value	>0.99	0.03	0.12	0.15	0.006	0.03	0.03	0.007	0.03	0.03	0.67	0.002
P value summary	ns	*	ns	ns	**	*	*	**	*	*	ns	**
Significantly different (P < 0.05)?	No	Yes	No	No	Yes	Yes	Yes	Yes	Yes	Yes	No	Yes
One- or two-tailed P value?	Two-tailed	Two-tailed	Two-tailed	Two-tailed	Two-tailed	Two-tailed	Two-tailed	Two-tailed	Two-tailed	Two-tailed	Two-tailed	Two-tailed
t, df or *Sum of ranks in column I,U*	*105 , 105*	t=2.296, df=18	t=1.637, df=18	t=1.488, df=18	t=3.361, df=10.87	*133.5 , 76.50*	t=2.296, df=18	*139.5 , 70.50*	t=2.311, df=18	t=2.341, df=18	*99 , 111*	*t=3.532, df=18*

**How big is the difference?**												
Mean of column A or *Median of column I*	*2.750, n=10*	3.76	3.76	2.83	03.08	*2.800, n=10*	3.17	*2.650, n=10*	2.77	3.23	*2.750, n=10*	*3.32*
Mean of column M or *Median of column U*	*2.750, n=10*	2.67	3.13	2.41	2.31	*2.350, n=10*	2.4	*2.000, n=10*	2.13	2.42	*3.000, n=10*	*2.45*
Difference between means (M - A) ± SEM or *Difference: Actual*	*0*	-1.090 ± 0.4748	-0.6300 ± 0.3848	-0.4200 ± 0.2823	-0.7700 ± 0.2291	*-0.45*	-0.7700 ± 0.3353	*-0.65*	-0.6400 ± 0.2769	-0.8100 ± 0.3460	*0.25*	*-0.8700 ± 0.2463*
95% confidence interval or *Difference: Hodges-Lehmann*	*0*	-2.087 to -0.09258	-1.438 to 0.1784	-1.013 to 0.1730	-1.275 to -0.2650	*-0.5*	-1.475 to -0.06547	*-0.6*	-1.222 to -0.05820	-1.537 to -0.08299	*0.3*	*-1.388 to -0.3525*
R squared (eta squared)		0.2265	0.1296	0.1095	0.5096		0.2265		0.2288	0.2334		0.4093

**F test to compare variances**												
F, DFn, Dfd	1.499, 9, 9	2.558, 9, 9	1.695, 9, 9	2.052, 9, 9	9.523, 9, 9	1.007, 9, 9	3.518, 9, 9	2.373, 9, 9	1.674, 9, 9	2.882, 9, 9	1.040, 9, 9	2.728, 9, 9
P value	0.56	0.18	0.44	0.3	0.003	>0.99	0.07	0.21	0.45	0.13	0.95	0.15
P value summary	ns	ns	ns	ns	**	ns	ns	ns	ns	ns	ns	ns
Significantly different (P < 0.05)?	No	No	No	No	Yes	No	No	No	No	No	No	No

**Descriptive statistics**	CD Male			KD Male		
Days	Mean	SEM	N	Mean	SEM	N
1	2.8	0.2	10	2.9	0.3	10
3	3.8	0.4	10	2.7	0.3	10
5	3.8	0.2	10	3.1	0.3	10
8	2.8	0.2	10	2.4	0.2	10
10	3.1	0.2	10	2.3	0.1	10
12	2.9	0.1	10	2.5	0.1	10
15	3.2	0.3	10	2.4	0.2	10
17	2.6	0.1	10	2.1	0.1	10
19	2.8	0.2	10	2.1	0.2	10
22	3.2	0.3	10	2.4	0.2	10
24	3.1	0.3	10	3.2	0.3	10
26	3.3	0.2	10	2.5	0.1	10

Supplementary Table 26: Statistical analysis of female's blood lactate datasets

		Blood lacate / Mouse (mM)																			
		CD Female										KD Female									
	Cohort	A			B			C				A			B			C			
	Mouse n°	1	2	3	1	2	3	1	2	3	4	1	2	3	1	2	3	1	2	3	4
Day	1	2.6	2.4	2.2	3.0	2.2	2.0	1.8	2.0	2.5	2.4	2.1	2.9	2.4	2.8	2.3	3.1	2.1	2.7	2.6	1.9
	3	4.8	3.3	2.5	2.1	2.6	2.2	3.3	3.3	2.9	2.6	3.6	2.1	2.2	2.3	2.6	2.3	2.7	2.4	2.7	1.9
	5	4.4	4.4	2.7	3.0	3.0	1.7	2.6	3.1	2.5	2.6	3.3	3.0	2.0	2.4	1.9	1.9	2.1	1.9	1.9	1.6
	8	4.1	2.8	2.8	3.2	2.6	2.2	3.0	3.9	4.0	2.8	2.1	2.3	2.2	2.3	2.4	2.7	3.0	3.5	2.4	2.3
	10	3.6	2.9	3.2	1.9	2.7	2.6	2.7	2.8	4.3	3.0	2.5	2.2	2.0	1.7	2.2	1.9	2.3	1.7	2.0	1.6
	12	3.6	3.0	3.2	2.5	2.9	2.7	2.6	2.8	4.0	2.2	2.1	3.1	1.6	2.4	2.3	2.0	1.7	2.5	2.7	1.9
	15	3.3	3.7	3.2	2.5	2.9	3.1	1.5	3.0	2.9	3.4	2.6	2.9	3.0	2.2	2.5	2.4	2.3	1.8	2.1	1.9
	17	3.9	3.2	2.7	3.1	2.6	2.4	3.4	3.6	4.0	3.1	1.6	2.6	1.7	2.2	2.6	1.0	2.0	4.0	2.5	2.2
	19	2.1	2.9	1.8	3.2	3.2	1.8	1.8	2.3	3.2	2.5	2.2	2.0	1.6	2.1	2.1	2.2	1.8	2.4	2.8	2.3
	22	3.9	3.2	2.8	2.2	5.4	2.9	2.5	2.4	3.3	2.3	2.0	2.5	2.8	1.9	3.4	2.6	3.0	2.0	2.1	1.6
	24	2.9	3.3	2.4	5.0	3.2	3.7	2.8	2.7	2.8	2.0	2.2	2.4	1.8	2.8	3.3	2.1	2.4	2.2	2.3	2.2
	26	2.1	2.6	2.9	2.5	4.2	3.3	2.6	3.5	2.6	2.3	2.4	2.9	3.2	2.5	2.4	2.8	3.0	3.7	2.9	1.8

Univariate Type III Repeated-Measures ANOVA Assuming Sphericity

```
Sum Sq num Df Error SS den Df   F value    Pr(>F)
(Intercept)    1664.27      1   14.822     18 2021.1719 < 2.2e-16 ***
Treatment        19.15      1   14.822     18   23.2610 0.0001364 ***
Time              5.69     11   60.766    198    1.6868 0.0785319 .
Treatment:Time    6.34     11   60.766    198    1.8773 0.0442162 *
---
Signif. codes:  0 ‘***’ 0.001 ‘**’ 0.01 ‘*’ 0.05 ‘.’ 0.1 ‘ ’ 1
```

Mauchly Tests for Sphericity

```
Test statistic p-value
Time              0.0042559 0.19538
Treatment:Time    0.0042559 0.19538
```

Greenhouse-Geisser and Huynh-Feldt Corrections
for Departure from Sphericity

```
GG eps Pr(>F[GG])
Time           0.55205    0.13007
Treatment:Time 0.55205    0.09022 .
---
Signif. codes:  0 ‘***’ 0.001 ‘**’ 0.01 ‘*’ 0.05 ‘.’ 0.1 ‘ ’ 1

HF eps Pr(>F[HF])
Time           0.8641612 0.09119275
Treatment:Time 0.8641612 0.05462743
```

Normal distribution: D'Agostino & Pearson test

**Control Diet datasets**	CD 1	CD 3	CD 5	CD 8	CD 10	CD 12	CD 15	CD 17	CD 19	CD 22	CD 24	CD 26
K2	1.0	7.7	1.4	1.1	2.7	1.7	9.6	0.6	4.1	9.6	7.5	3.3
P value	0.6	0.02	0.5	0.6	0.3	0.4	0.01	0.8	0.1	0.01	0.02	0.2
Passed normality test (alpha=0.05)?	Yes	No	Yes	Yes	Yes	Yes	No	Yes	Yes	No	No	Yes
P value summary	ns	*	ns	ns	ns	ns	**	ns	ns	**	*	ns
Number of values	10	10	10	10	10	10	10	10	10	10	10	10

**Ketogenic Diet datasets**	KD 1	KD 3	KD 5	KD 8	KD 10	KD 12	KD 15	KD 17	KD 19	KD 22	KD 24	KD 26
K2	0.9	8.6	4.1	7.2	0.5	0.5	0.3	4.1	1.2	0.6	6.0	0.6
P value	0.6	0.01	0.1	0.03	0.8	0.8	0.9	0.1	0.6	0.7	0.1	0.7
Passed normality test (alpha=0.05)?	Yes	No	Yes	No	Yes	Yes	Yes	Yes	Yes	Yes	No	Yes
P value summary	ns	*	ns	*	ns	ns	ns	ns	ns	ns	*	ns
Number of values	10	10	10	10	10	10	10	10	10	10	10	10

**Type of Test**	Unpaired T-test	Mann-Whitney T-test	Unpaired T-test	Mann-Whitney T-test	Welch's T-test	Unpaired T-test	Mann-Whitney T-test	Unpaired T-test	Unpaired T-test	Mann-Whitney T-test	Mann-Whitney T-test	Welch's T-test
Data organization	CD 1 vs KD 1	CD 3 vs KD 3	CD 5 vs KD 5	CD 8 vs KD 8	CD 10 vs KD 10	CD 12 vs KD 12	CD 15 vs KD 15	CD 17 vs KD 17	CD 19 vs KD 19	CD 22 vs KD 22	CD 24 vs KD 24	CD 26 vs KD 26
Number of values	10 vs 10	10 vs 10	10 vs 10	10 vs 10	10 vs 10	10 vs 10	10 vs 10	10 vs 10	10 vs 10	10 vs 10	10 vs 10	10 vs 10
P value	0.29	0.16	0.02	0.02	<0.001	0.005	0.01	0.005	0.15	0.07	0.02	0.7
P value summary	ns	ns	*	*	***	**	*	**	ns	ns	*	ns
Significantly different (P < 0.05)?	No	No	Yes	Yes	Yes	Yes	Yes	Yes	No	No	Yes	No
One- or two-tailed P value?	Two-tailed	Two-tailed	Two-tailed	Two-tailed	Two-tailed	Two-tailed	Two-tailed	Two-tailed	Two-tailed	Two-tailed	Two-tailed	Two-tailed
t, df or *Sum of ranks in column I,U*	t=1.085, df=18	*124 , 86*	t=2.538, df=18	*135 , 75*	t=4.317, df=12.60	t=3.218, df=18	*138 , 72*	t=3.157, df=18	t=1.517, df=18	*129 , 81*	*135.5 , 74,50*	t=0.3858, df=18

**How big is the difference?**												
Mean of column A or *Median of column I*	2.31	*2.750, n=10*	3	*2.900, n=10*	2.97	2.95	*3.050, n=10*	3.2	2.48	*2.850, n=10*	*2.850, n=10*	2.86
Mean of column M or *Median of column U*	2.49	*2.350, n=10*	2.2	*2.350, n=10*	02.01	2.23	*2.350, n=10*	2.24	2.15	*2.300, n=10*	*2.250, n=10*	2.76
Difference between means (M - A) ± SEM or *Difference: Actual*	0.1800 ± 0.1659	*-0.4*	-0.8000 ± 0.3152	*-0.55*	-0.9600 ± 0.2224	-0.7200 ± 0.2238	*-0.7*	-0.9600 ± 0.3041	-0.3300 ± 0.2176	*-0.55*	*-0.6*	-0.1000 ± 0.2592
95% confidence interval or *Difference: Hodges-Lehmann*	-0.1686 to 0.5286	*-0.4*	-1.462 to -0.1378	*-0.5*	-1.442 to -0.4781	-1.190 to -0.2499	*-0.7*	-1.599 to -0.3211	-0.7871 to 0.1271	*-0.5*	*-0.6*	-0.6446 to 0.4446
R squared (eta squared)	0.06136		0.2636		0.5966	0.3652		0.3563	0.1133			0.008199

**F test to compare variances**												
F, DFn, Dfd	1.275, 9, 9	2.768, 9, 9	2.361, 9, 9	2.258, 9, 9	4.787, 9, 9	1.321, 9, 9	2.345, 9, 9	2.202, 9, 9	3.416, 9, 9	2.931, 9, 9	3.956, 9, 9	1.495, 9, 9
P value	0.72	0.15	0.22	0.24	0.03	0.68	0.22	0.26	0.08	0.12	0.05	0.56
P value summary	ns	ns	ns	ns	*	ns	ns	ns	ns	ns	ns	ns
Significantly different (P < 0.05)?	No	No	No	No	Yes	No	No	No	No	No	No	No

**Descriptive statistics**	CD Female			KD Female		
Days	Mean	SEM	N	Mean	SEM	N
1	2.3	0.1	10	2.5	0.1	10
3	3.0	0.2	10	2.5	0.1	10
5	3.0	0.3	10	2.2	0.2	10
8	3.1	0.2	10	2.5	0.1	10
10	3.0	0.2	10	2.0	0.1	10
12	3.0	0.2	10	2.2	0.1	10
15	3.0	0.2	10	2.4	0.1	10
17	3.2	0.2	10	2.2	0.3	10
19	2.5	0.2	10	2.2	0.1	10
22	3.1	0.3	10	2.4	0.2	10
24	3.1	0.3	10	2.4	0.1	10
26	2.9	0.2	10	2.8	0.2	10

Supplementary Table 27: Statistical analysis of blood lactate between male and female the first and last days of the diet intervention

Blood Lacate, mM			
$Male_{D1}$	$Female_{D1}$	$Male_{D26}$	$Female_{D26}$
4.6	2.6	3.5	2.1
2.8	2.4	3.8	2.6
2.3	2.2	2.3	2.9
2.8	3	2.5	2.5
3.1	2.2	3	4.2
2.7	2	3.6	3.3
2.2	1.8	2.8	2.6
2.6	2	3.3	3.5
3.1	2.5	4.3	2.6
2.1	2.4	4.1	2.3
4.9	2.1		
3	2.9		
1.9	2.4		
2.4	2.8		
2.7	2.3		
2.8	3.1		
2.4	2.1		
3.7	2.7		
2.9	2.6		
2.1	1.9		

**Type of Test**	Mann Whitney test
Data organization	$Male_{D1}$ vs $Female_{D1}$
Number of values	20 vs 20
P value	0.04
Exact or approximate P value?	Exact
P value summary	*
Significantly different (P < 0.05)?	Yes
One- or two-tailed P value?	Two-tailed
Sum of ranks in column A,B	487.5 . 332.5
Mann-Whitney U	122.5

**Difference between medians**	
Median of column A	2.750. n=20
Median of column B	2.400. n=20
Difference: Actual	-0.35
Difference: Hodges-Lehmann	-0.3

**F test to compare variances**	
F, DFn, Dfd	4.322. 19. 19
P value	0.002
P value summary	**
Significantly different (P < 0.05)?	Yes

**Type of Test**	Unpaired T-test
Data organization	$Male_{D26}$ vs $Female_{D26}$
Number of values	10 vs 10
P value	0.13
P value summary	ns
Significantly different (P < 0.05)?	No
One- or two-tailed P value?	Two-tailed
t, df	t=1.58. df=18

**How big is the difference?**	
Mean of column C	3.32
Mean of column D	2.86
Difference between means (D - C) ± SEM	-0.460 ± 0.291
95% confidence interval	-1.07 to 0.151
R squared (eta squared)	0.122

**F test to compare variances**	
F, DFn, Dfd	1.10. 9. 9
P value	0.89
P value summary	ns
Significantly different (P < 0.05)?	No

**Normality of Residuals**	$Male_{D1}$	$Female_{D1}$	$Male_{D26}$	$Female_{D26}$
Test name: D'Agostino-Pearson omnibus (K2)				
Statistics	11.0	1.1	0.7	3.3
P value	0.0	0.6	0.7	0.19
Passed normality test (alpha=0,05)?	No	Yes	Yes	Yes
P value summary	**	ns	ns	ns

**Descriptive statistics**	$Male_{D1}$	$Female_{D1}$	$Male_{D26}$	$Female_{D26}$
Number of values	20	20	10	10
Minimum	1.9	1.8	2.3	2.1
Maximum	4.9	3.1	4.3	4.2
Range	3	1.3	2.0	2.1
Mean	2.9	2.4	3.3	2.9
Std. Deviation	0.8	0.4	0.7	0.6
Std. Error of Mean	0.2	0.1	0.2	0.2

Supplementary Table 28: Statistical analysis of blood lactate rate between CD and KD male,and CD and KD female

**Type of Test**	Nonlinear regression: Two phase decay

**Best-fit values**	CD Male	KD Male	CD Female	KD Female
Y0	3.4	3.1	-2.2	2.6
Plateau	2.8	2.4	8.5	2.2
PercentFast	Unstable	Unstable	Unstable	Unstable
KFast	0.1	0.2	2.1	0.5
KSlow	4.9E-32	0.2	4.9E-32	4.9E-32
Half Life (Slow)	1.4E+31	3.1	1.4E+31	1.4E+31
Half Life (Fast)	6.0	3.1	0.3	1.3
Tau (slow)	2.0E+31	4.5	2.0E+31	2.0E+31
Tau (fast)	8.7	4.5	0.5	1.8
Rate constant ratio	2.3E+30	1.0	4.2E+31	1.1E+31

Supplementary Table 29: Statistical analysis of blood lacate variation between CD and KD male,and CD and KD female

		Blood lactate change (%)			
Cohort	Mouse n°	CD Male	KD Male	CD Female	KD Female
A	1	-24	-55	-19	14
	2	36	-37	8	0
	3	0	0	32	33
B	1	-11	33	-17	-11
	2	-3	-4	67	4
	3	33	-21	43	-10
C	1	27	13	44	43
	2	27	-27	75	37
	3	39	-10	4	12
	4	95	19	-4	-5

Test for normal distribution

**D'Agostino & Pearson test**	CD Male	KD Male	CD Female	KD Female
K2	3.1	0.1	1.6	1.8
P value	0.2	1.0	0.5	0.4
Passed normality test (alpha=0.05)?	Yes	Yes	Yes	Yes
P value summary	ns	ns	ns	ns

**Type of Test**	ANOVA test
F	2.61
P value	0.07
P value summary	ns
Significant diff. among means (P < 0.05)?	No
R squared	0.179

**Tukey's multiple comparisons test**	**Mean Diff.**	**95.00% CI of diff.**	**Below threshold?**	**Summary**	**Adjusted P Value**
CD Male vs. KD Male	30.9	-4.23 to 66.0	No	ns	0.1
CD Male vs. CD Female	-1.41	-36.5 to 33.7	No	ns	>0.99
CD Male vs. KD Female	10.3	-24.8 to 45.4	No	ns	0.86
KD Male vs. CD Female	-32.3	-67.4 to 2.82	No	ns	0.08
KD Male vs. KD Female	-20.6	-55.7 to 14.5	No	ns	0.4
CD Female vs. KD Female	11.7	-23.4 to 46.8	No	ns	0.81

**Test details**	**Mean 1**	**Mean 2**	**Mean Diff.**	**SE of diff.**	**n1**	**n2**	**q**	**DF**
CD Male vs. KD Male	21.9	-8.98	30.9	13	10	10	3.35	36
CD Male vs. CD Female	21.9	23.3	-1.41	13	10	10	0.15	36
CD Male vs. KD Female	21.9	11.6	10.3	13	10	10	1.12	36
KD Male vs. CD Female	-8.98	23.3	-32.3	13	10	10	3.50	36
KD Male vs. KD Female	-8.98	11.6	-20.6	13	10	10	2.23	36
CD Female vs. KD Female	23.3	11.6	11.7	13	10	10	1.27	36

**Descriptive statistics**	CD Male	KD Male	CD Female	KD Female
Number of values	10	10	10	10
Mean	21.9	-9.0	23.3	11.6
Std. Deviation	33.9	26.9	33.8	19.8
Std. Error of Mean	10.7	8.5	10.7	6.3

Supplementary Table 30: Multiple linear regression analysis of GFAP and ALDH1L1 positive and negative cells

Sample	Total cell number
CL220405_Sample1_ROI1	18
CL220405_Sample1_ROI2	15
CL220405_Sample1_ROI3	16
CL220405_Sample1_ROI4	12
CL220405_Sample2_ROI1	9
CL220405_Sample2_ROI2	22
CL220405_Sample2_ROI3	18
CL220405_Sample2_ROI4	10

GFAP data			
Sample	ROI	GFAP	Cells (n)
CL220405_Sample1	ROI1	GFAP+	16
CL220405_Sample1	ROI1	GFAP-	2
CL220405_Sample1	ROI2	GFAP+	11
CL220405_Sample1	ROI2	GFAP-	4
CL220405_Sample1	ROI3	GFAP+	8
CL220405_Sample1	ROI3	GFAP-	8
CL220405_Sample1	ROI4	GFAP+	8
CL220405_Sample1	ROI4	GFAP-	4
CL220405_Sample2	ROI1	GFAP+	7
CL220405_Sample2	ROI1	GFAP-	2
CL220405_Sample2	ROI2	GFAP+	16
CL220405_Sample2	ROI2	GFAP-	6
CL220405_Sample2	ROI3	GFAP+	13
CL220405_Sample2	ROI3	GFAP-	5
CL220405_Sample2	ROI4	GFAP+	6
CL220405_Sample2	ROI4	GFAP-	3

GFAP data			
Sample	ROI	GFAP	Cells (%)
CL220405_Sample1	ROI1	GFAP+	89
CL220405_Sample1	ROI1	GFAP-	11
CL220405_Sample1	ROI2	GFAP+	73
CL220405_Sample1	ROI2	GFAP-	27
CL220405_Sample1	ROI3	GFAP+	50
CL220405_Sample1	ROI3	GFAP-	50
CL220405_Sample1	ROI4	GFAP+	67
CL220405_Sample1	ROI4	GFAP-	33
CL220405_Sample2	ROI1	GFAP+	78
CL220405_Sample2	ROI1	GFAP-	22
CL220405_Sample2	ROI2	GFAP+	73
CL220405_Sample2	ROI2	GFAP-	27
CL220405_Sample2	ROI3	GFAP+	72
CL220405_Sample2	ROI3	GFAP-	28
CL220405_Sample2	ROI4	GFAP+	60
CL220405_Sample2	ROI4	GFAP-	30

ALDH1L1 data			
Sample	ROI	ALDH1L1	Cells (n)
CL220405_Sample1	ROI1	ALDH1L1+	13
CL220405_Sample1	ROI1	ALDH1L1-	5
CL220405_Sample1	ROI2	ALDH1L1+	13
CL220405_Sample1	ROI2	ALDH1L1-	2
CL220405_Sample1	ROI3	ALDH1L1+	9
CL220405_Sample1	ROI3	ALDH1L1-	7
CL220405_Sample1	ROI4	ALDH1L1+	12
CL220405_Sample1	ROI4	ALDH1L1-	0
CL220405_Sample2	ROI1	ALDH1L1+	7
CL220405_Sample2	ROI1	ALDH1L1-	2
CL220405_Sample2	ROI2	ALDH1L1+	17
CL220405_Sample2	ROI2	ALDH1L1-	5
CL220405_Sample2	ROI3	ALDH1L1+	17
CL220405_Sample2	ROI3	ALDH1L1-	1
CL220405_Sample2	ROI4	ALDH1L1+	8
CL220405_Sample2	ROI4	ALDH1L1-	2

ALDH1L1 data			
Sample	ROI	ALDH1L1	Cells (%)
CL220405_Sample1	ROI1	ALDH1L1+	72
CL220405_Sample1	ROI1	ALDH1L1-	28
CL220405_Sample1	ROI2	ALDH1L1+	87
CL220405_Sample1	ROI2	ALDH1L1-	13
CL220405_Sample1	ROI3	ALDH1L1+	56
CL220405_Sample1	ROI3	ALDH1L1-	44
CL220405_Sample1	ROI4	ALDH1L1+	100
CL220405_Sample1	ROI4	ALDH1L1-	0
CL220405_Sample2	ROI1	ALDH1L1+	78
CL220405_Sample2	ROI1	ALDH1L1-	22
CL220405_Sample2	ROI2	ALDH1L1+	77
CL220405_Sample2	ROI2	ALDH1L1-	23
CL220405_Sample2	ROI3	ALDH1L1+	94
CL220405_Sample2	ROI3	ALDH1L1-	6
CL220405_Sample2	ROI4	ALDH1L1+	80
CL220405_Sample2	ROI4	ALDH1L1-	20

**Descriptives statistics**	$GFAP^{+}$ (%)	$GFAP^{-}$ (%)	$ALDH1l1^{+}$ (%)	$ALDH1l1^{-}$ (%)
Minimum	50	11	56	0
Maximum	89	50	100	44
Range	39	39	44	44
Mean	70	29	81	20
Std. Deviation	12	11	14	14
Std. Error of Mean	4	4	5	5

Multiple linear regression analysis of GFAP +/- cells

**Analysis of Variance**	**SS**	**DF**	**MS**	**F (DFn, DFd)**	**P value**
Regression	6997	5	1399	F (5, 10) = 7.91	P=0.003
Sample	6.25	1	6.25	F (1, 10) = 0.0353	P=0.85
ROI	18.8	3	6.25	F (3, 10) = 0.0353	P>0.99
GFAP	6972	1	6972	F (1, 10) = 39.4	P<0.001
Residual	1769	10	177		
Total	8766	15			

**Parameter estimates**	**Variable**	**Estimate**	**Standard error**	**95% CI (asymptotic)**	**|t|**	**P value**	**P value summary**
β0	Intercept	71.5	8.14	53.4 to 89.6	8.78	<0.001	***
β1	Sample[CL220405_Sample2]	-1.25	6.65	-16.1 to 13.6	0.188	0.85	ns
β2	ROI[ROI2]	-5.88E-15	9.4	-21.0 to 21.0	6.26E-16	>0.99	ns
β3	ROI[ROI3]	-3.55E-15	9.4	-21.0 to 21.0	3.78E-16	>0.99	ns
β4	ROI[ROI4]	-2.5	9.4	-23.5 to 18.5	0.266	0.8	ns
β5	GFAP[GFAP-]	-41.8	6.65	-56.6 to -26.9	6.28	<0.001	***

**Goodness of Fit**	
Degrees of Freedom	10
R squared	0.798

**Multicollinearity**	**Variable**	**VIF**	**R2 with other variables**
β0	Intercept		
β1	Sample[CL220405_Sample2]	1	0
β2	ROI[ROI2]	1.5	0.333
β3	ROI[ROI3]	1.5	0.333
β4	ROI[ROI4]	1.5	0.333
β5	GFAP[GFAP-]	1	0

**Normality of Residuals**	**Statistics**	**P value**	**Passed normality test (alpha=0.05)?**	**P value summary**
D'Agostino-Pearson omnibus (K2)	0.749	0.69	Yes	ns

Multiple linear regression analysis of ALDH1L1 +/- cells

**Analysis of Variance**	**SS**	**DF**	**MS**	**F (DFn, DFd)**	**P value**
Regression	14884	5	2977	F (5, 10) = 11.5	P<0.001
Sample	0	1	0	F (1, 10) = 0.00	P>0.99
ROI	0	3	0	F (3, 10) = 0.00	P>0.99
GFAP	14884	1	14884	F (1, 10) = 57.4	P<0.001
Residual	2592	10	259		
Total	17476	15			

**Parameter estimates**	**Variable**	**Estimate**	**Standard error**	**95% CI (asymptotic)**	**|t|**	**P value**	**P value summary**
β0	Intercept	80.5	9.86	58.5 to 102	8.17	<0.001	***
β1	Sample[CL220405_Sample2]	0	08.05	-17.9 to 17.9	0	>0.99	ns
β2	ROI[ROI2]	0	11.4	-25.4 to 25.4	0	>0.99	ns
β3	ROI[ROI3]	0	11.4	-25.4 to 25.4	0	>0.99	ns
β4	ROI[ROI4]	0	11.4	-25.4 to 25.4	0	>0.99	ns
β5	ALDH1L1[ALDH1l1-]	-61	08.05	-78.9 to -43.1	7.58	<0.001	***

**Goodness of Fit**	
Degrees of Freedom	10
R squared	0.852

**Multicollinearity**	**Variable**	**VIF**	**R2 with other variables**
β0	Intercept		
β1	Sample[CL220405_Sample2]	1	0
β2	ROI[ROI2]	1.5	0.333
β3	ROI[ROI3]	1.5	0.333
β4	ROI[ROI4]	1.5	0.333
β5	ALDH1L1[ALDH1l1-]	1	0

**Normality of Residuals**	**Statistics**	**P value**	**Passed normality test (alpha=0.05)?**	**P value summary**
D'Agostino-Pearson omnibus (K2)	0.0249	0.99	Yes	ns

Supplementary Table 31: Statistical analysis of astrocyte and neuron lactate between CD and KD males,and CD and KD females

Brain sample ID	Astrocyte's lactate (µM)	Sex	Diet	Sample protein (µg/mL)	Normalized astrocyte's lactate (µM/(µg/mL))
BC061	0.6	Male	CD	108.3	5.5E-03
BC067	1.0	Male	CD	431.0	2.3E-03
BC073	0.9	Male	CD	27.0	3.3E-02
BC063	0.5	Male	KD	146.7	3.4E-03
BC069	0.9	Male	KD	160.3	5.6E-03
BC075	0.5	Male	KD	66.7	7.5E-03
BC095	0.4	Female	CD	84.0	4.8E-03
BC101	0.0	Female	CD	39.7	0.0E+00
BC107	1.1	Female	CD	134.3	8.2E-03
BC097	1.1	Female	KD	220.7	5.0E-03
BC103	0.0	Female	KD	36.0	0.0E+00
BC109	1.4	Female	KD	123.0	1.1E-02

Brain sample ID	Neuron's lactate (µM)	Sex	Diet	Sample protein (µg/mL)	Normalized neuron's lactate (µM/(µg/mL))
BC064	0.02	Male	CD	184.3	9.5E-05
BC070	0.2	Male	CD	361.0	6.8E-04
BC076-N1	0.5	Male	CD	747.0	6.5E-04
BC066	0.2	Male	KD	268.0	8.2E-04
BC072	1.2	Male	KD	545.0	2.1E-03
BC098	0.2	Female	CD	97.3	1.7E-03
BC104	0.2	Female	CD	213.3	1.1E-03
BC110	1.4	Female	CD	453.0	3.1E-03
BC100	0.8	Female	KD	410.3	2.0E-03
BC106	0.3	Female	KD	145.7	2.2E-03
BC112	1.2	Female	KD	567.0	2.2E-03

**Kruskal-Wallis test**	Astrocyte					
P value	0.9					
Exact or approximate P value?	Exact					
P value summary	ns					
Do the medians vary signif. (P < 0.05)?	No					
Number of groups	4.0					
Kruskal-Wallis statistic	0.5					
**Dunn's multiple comparisons test**	**Mean rank diff.**	**Significant?**	**Summary**	**Adjusted P Value**		
CDM vs. KDM	0.7	No	ns	>0.99		
CDM vs. CDF	1.8	No	ns	>0.99		
CDM vs. KDF	1.5	No	ns	>0.99		
KDM vs. CDF	1.2	No	ns	>0.99		
KDM vs. KDF	0.8	No	ns	>0.99		
CDF vs. KDF	-0.3	No	ns	>0.99		
**Test details**	**Mean rank 1**	**Mean rank 2**	**Mean rank diff.**	**n1**	**n2**	**Z**
CDM vs. KDM	7.5	6.8	0.7	3.0	3	0.2
CDM vs. CDF	7.5	5.7	1.8	3.0	3	0.6
CDM vs. KDF	7.5	6.0	1.5	3.0	3	0.5
KDM vs. CDF	6.8	5.7	1.2	3.0	3	0.4
KDM vs. KDF	6.8	6.0	0.8	3.0	3	0.3
CDF vs. KDF	5.7	6.0	-0.3	3.0	3	0.1

**Kruskal-Wallis test**	Neuron					
P value	0.04					
Exact or approximate P value?	Exact					
P value summary	*					
Do the medians vary signif. (P < 0.05)?	Yes					
Number of groups	4					
Kruskal-Wallis statistic	6.85					
**Dunn's multiple comparisons test**	**Mean rank diff.**	**Significant?**	**Summary**	**Adjusted P Value**		
CDM vs. KDM	-4	No	ns	>0.99		
CDM vs. CDF	-5.3	No	ns	0.3		
CDM vs. KDF	-6.7	No	ns	0.1		
KDM vs. CDF	-1.3	No	ns	>0.99		
KDM vs. KDF	-2.7	No	ns	>0.99		
CDF vs. KDF	-1.3	No	ns	>0.99		
**Test details**	**Mean rank 1**	**Mean rank 2**	**Mean rank diff.**	**n1**	**n2**	**Z**
CDM vs. KDM	2.0	6.0	-4.0	3	2	1.3
CDM vs. CDF	2.0	7.3	-5.3	3	3	2.0
CDM vs. KDF	2.0	8.7	-6.7	3	3	2.5
KDM vs. CDF	6.0	7.3	-1.3	2	3	0.4
KDM vs. KDF	6.0	8.7	-2.7	2	3	0.9
CDF vs. KDF	7.3	8.7	-1.3	3	3	0.5

	Astrocyte				Neuron			
	Male		Female		Male		Female	
**Descriptive statistics**	CD	KD	CD	KD	CD	KD	CD	KD
Number of values	3	3	3	3	3	3	3	3
Minimum	2.0E-03	3.0E-03	0.0E+00	0.0E+00	1.0E-04	8.0E-04	1.1E-03	2.0E-03
Maximum	3.2E-02	7.0E-03	8.0E-03	1.1E-02	7.0E-04	2.1E-03	3.1E-03	2.2E-03
Range	3.0E-02	4.0E-03	8.0E-03	1.1E-02	6.0E-04	1.3E-03	2.0E-03	2.0E-04
Mean	1.3E-02	5.3E-03	4.3E-03	5.3E-03	5.0E-04	1.5E-03	2.0E-03	2.1E-03
Std. Deviation	1.6E-02	2.1E-03	4.0E-03	5.5E-03	3.5E-04	9.2E-04	1.0E-03	1.2E-04
Std. Error of Mean	9.4E-03	1.2E-03	2.3E-03	3.2E-03	2.0E-04	6.5E-04	5.9E-04	6.7E-05

Supplementary Table 32: Statistical analysis of neuron glucose and brain glycogen between CD and KD male,and CD and KD female

Brain sample ID	Neuron's glucose (µM)	Sex	Diet	Sample protein (µg/mL)	Normalized neuron's glucose (µM/(µg/mL))
BC064	91.7	Male	CD	184.3	0.5
BC070	113.2	Male	CD	361.0	0.3
BC076-N1	131.9	Male	CD	747.0	0.2
BC066	89.2	Male	KD	268.0	0.3
BC072	125.1	Male	KD	545.0	0.2
BC098	41.3	Female	CD	97.3	0.4
BC104	111.8	Female	CD	213.3	0.5
BC110	127.8	Female	CD	453.0	0.3
BC100	113.2	Female	KD	410.3	0.3
BC106	101.8	Female	KD	145.7	0.7
BC112	117.8	Female	KD	567.0	0.2

**Descriptive statistics**	CD Male	KD Male	CD Female	KD Female
Number of values	3	3	3	3
Minimum	0.2	0.2	0.3	0.2
Maximum	0.5	0.3	0.5	0.7
Range	0.3	0.1	0.2	0.5
Mean	0.3	0.3	0.4	0.4
Std. Deviation	0.2	0.1	0.1	0.3
Std. Error of Mean	0.1	0.1	0.1	0.2

**Kruskal-Wallis test**	Neuron					
P value	0.85					
Exact or approximate P value?	Exact					
P value summary	ns					
Do the medians vary signif. (P < 0.05)?	No					
Number of groups	4					
Kruskal-Wallis statistic	0.9285					
**Dunn's multiple comparisons test**	**Mean rank diff.**	**Significant?**	**Summary**	**Adjusted P Value**		
CDM vs. KDM	0.3	No	ns	>0.99		
CDM vs. CDF	-2.2	No	ns	>0.99		
CDM vs. KDF	-0.5	No	ns	>0.99		
KDM vs. CDF	-2.5	No	ns	>0.99		
KDM vs. KDF	-0.8	No	ns	>0.99		
CDF vs. KDF	1.7	No	ns	>0.99		
**Test details**	**Mean rank 1**	**Mean rank 2**	**Mean rank diff.**	**n1**	**n2**	**Z**
CDM vs. KDM	5.3	5.0	0.3	3	2	0.1
CDM vs. CDF	5.3	7.5	-2.2	3	3	0.8
CDM vs. KDF	5.3	5.8	-0.5	3	3	0.2
KDM vs. CDF	5.0	7.5	-2.5	2	3	0.8
KDM vs. KDF	5.0	5.8	-0.8	2	3	0.3
CDF vs. KDF	7.5	5.8	1.7	3	3	0.6

Brain sample ID	Brain glycogen (µM)	Sex	Diet	Sample protein (µg/mL)	Normalized brain glycogen (µM/(µg/mL))
#10.1	23.3	Male	CD	1744.0	0.01
#10.2	28.4	Male	CD	1658.0	0.02
#10.3	14.5	Male	CD	1171.0	0.01
#30.1	11.7	Male	KD	1043.4	0.01
#30.2	10.1	Male	KD	1187.6	0.01
#30.3	21.0	Male	KD	1607.4	0.01
#10.1	12.6	Female	CD	1161.4	0.01
#10.2	10.5	Female	CD	1836.4	0.01
#10.3	6.3	Female	CD	1202.2	0.01
#30.1	18.5	Female	KD	1349.4	0.01
#30.2	17.8	Female	KD	1696.2	0.01
#30.3	14.1	Female	KD	1515.2	0.01

**Descriptive statistics**	CD Male	KD Male	CD Female	KD Female
Number of values	3	3	3	3
Minimum	1.2E-02	8.0E-03	5.0E-03	9.0E-03
Maximum	1.7E-02	1.3E-02	1.1E-02	1.4E-02
Range	5.0E-03	5.0E-03	6.0E-03	5.0E-03
Mean	1.4E-02	1.1E-02	7.3E-03	1.1E-02
Std. Deviation	2.6E-03	2.5E-03	3.2E-03	2.5E-03
Std. Error of Mean	1.5E-03	1.5E-03	1.9E-03	1.5E-03

**Kruskal-Wallis test**	Brain					
P value	0.13					
Exact or approximate P value?	Exact					
P value summary	ns					
Do the medians vary signif. (P < 0.05)?	No					
Number of groups	4					
Kruskal-Wallis statistic	5.572					
**Dunn's multiple comparisons test**	**Mean rank diff.**	**Significant?**	**Summary**	**Adjusted P Value**		
CDM vs. KDM	3.7	No	ns	>0.99		
CDM vs. CDF	6.8	No	ns	0.1		
CDM vs. KDF	2.8	No	ns	>0.99		
KDM vs. CDF	3.2	No	ns	>0.99		
KDM vs. KDF	-0.8	No	ns	>0.99		
CDF vs. KDF	-4.0	No	ns	>0.99		
**Test details**	**Mean rank 1**	**Mean rank 2**	**Mean rank diff.**	**n1**	**n2**	**Z**
CDM vs. KDM	9.8	6.2	3.7	3	3	1.3
CDM vs. CDF	9.8	3.0	6.8	3	3	2.3
CDM vs. KDF	9.8	7.0	2.8	3	3	1.0
KDM vs. CDF	6.2	3.0	3.2	3	3	1.1
KDM vs. KDF	6.2	7.0	-0.8	3	3	0.3
CDF vs. KDF	3.0	7.0	-4.0	3	3	1.4

Supplementary Table 33: Statistical analysis of astrocyte glutamine between CD and KD males,and CD and KD females

Brain sample ID	Astrocyte's glutamine (µM)	Sex	Diet	Sample protein (µg/mL)	Normalized astrocyte's glutamine (µM/(µg/mL))
BC061	0.0	Male	CD	108.3	0.0E+00
BC067	0.0	Male	CD	431.0	0.0E+00
BC073	0.0	Male	CD	27.0	0.0E+00
BC063	0.4	Male	KD	146.7	2.7E-03
BC069	0.3	Male	KD	160.3	2.1E-03
BC075	0.3	Male	KD	66.7	4.4E-03
BC095	0.1	Female	CD	84.0	1.7E-03
BC101	0.1	Female	CD	39.7	0.0E+00
BC107	0.4	Female	CD	134.3	2.7E-03
BC097	0.1	Female	KD	220.7	5.8E-04
BC103	0.01	Female	KD	36.0	2.6E-04
BC109	0.1	Female	KD	123.0	1.1E-03

**Descriptive statistics**	CD Male	KD Male	CD Female	KD Female
Number of values	3	3	3	3
Minimum	0	2.1E-03	1.7E-03	3.0E-04
Maximum	0	4.4E-03	2.7E-03	1.1E-03
Range	0	2.3E-03	1.0E-03	8.0E-04
Mean	0	3.1E-03	2.0E-03	6.7E-04
Std. Deviation	0	1.2E-03	5.8E-04	4.0E-04
Std. Error of Mean	0	6.9E-04	3.3E-04	2.3E-04

**Kruskal-Wallis test**						
P value	0.003					
Exact or approximate P value?	Approximate					
P value summary	**					
Do the medians vary signif. (P < 0.05)?	Yes					
Number of groups	8					
Kruskal-Wallis statistic	21.65					
**Dunn's multiple comparisons test**	**Mean rank diff.**	**Significant?**	**Summary**	**Adjusted P Value**		
KDM vs. CDF	2.0	No	ns	>0.99		
KDM vs. KDF	5.5	No	ns	>0.99		
CDF vs. KDF	3.5	No	ns	>0.99		
**Test details**	**Mean rank 1**	**Mean rank 2**	**Mean rank diff.**	**n1**	**n2**	**Z**
KDM vs. CDF	21.5	19.5	2	3	3	0.4
KDM vs. KDF	21.5	16	5.5	3	3	1.1
CDF vs. KDF	19.5	16	3.5	3	3	0.7

Supplementary Table 34: Statistical analysis of astrocyte and neuron glutamate between CD and KD male,and CD and KD female

Brain sample ID	Astrocyte's glutamate (μM)	Sex	Diet	Sample protein (μg/mL)	Normalized astrocyte's glutamate (μM/(μg/mL))
BC061	1.5	Male	CD	108.3	1.4E-02
BC067	2.4	Male	CD	431.0	5.6E-03
BC073	1.7	Male	CD	27.0	6.5E-02
BC063	1.3	Male	KD	146.7	8.8E-03
BC069	1.6	Male	KD	160.3	9.8E-03
BC075	1.0	Male	KD	66.7	1.5E-02
BC095	0.9	Female	CD	84.0	1.0E-02
BC101	0.3	Female	CD	39.7	0.0E+00
BC107	2.5	Female	CD	134.3	1.8E-02
BC097	1.3	Female	KD	220.7	5.9E-03
BC103	0.2	Female	KD	36.0	6.7E-03
BC109	1.3	Female	KD	123.0	1.0E-02

**Descriptive statistics**	CD Male	KD Male	CD Female	KD Female
Number of values	3	3	3	3
Minimum	5.6E-03	8.8E-03	0.0E+00	5.9E-03
Maximum	6.5E-02	1.5E-02	1.8E-02	1.1E-02
Range	5.9E-02	6.5E-03	1.8E-02	4.6E-03
Mean	2.8E-02	1.1E-02	9.6E-03	7.7E-03
Std. Deviation	3.2E-02	3.5E-03	9.2E-03	2.5E-03
Std. Error of Mean	1.8E-02	2.0E-03	5.3E-03	1.4E-03

**Kruskal-Wallis test**	Astrocyte
P value	0.86
Exact or approximate P value?	Exact
P value summary	ns
Do the medians vary signif. (P < 0.05)?	No
Number of groups	4
Kruskal-Wallis statistic	0.8974

**Dunn's multiple comparisons test**	**Mean rank diff.**	**Significant?**	**Summary**	**Adjusted P Value**
CDM vs. KDM	0.7	No	ns	>0.99
CDM vs. CDF	1.3	No	ns	>0.99
CDM vs. KDF	2.7	No	ns	>0.99
KDM vs. CDF	0.7	No	ns	>0.99
KDM vs. KDF	2.0	No	ns	>0.99
CDF vs. KDF	1.3	No	ns	>0.99

**Test details**	**Mean rank 1**	**Mean rank 2**	**Mean rank diff.**	**n1**	**n2**	**Z**
CDM vs. KDM	7.7	7.0	0.7	3	3	0.2
CDM vs. CDF	7.7	6.3	1.3	3	3	0.5
CDM vs. KDF	7.7	5.0	2.7	3	3	0.9
KDM vs. CDF	7.0	6.3	0.7	3	3	0.2
KDM vs. KDF	7.0	5.0	2.0	3	3	0.7
CDF vs. KDF	6.3	5.0	1.3	3	3	0.5

Brain sample ID	Neuron's glutamate (μM)	Sex	Diet	Sample protein (μg/mL)	Normalized neuron's glutamate (μM/(μg/mL))
BC064	0.4	Male	CD	184.3	2.2E-03
BC070	0.7	Male	CD	361.0	1.8E-03
BC076-N1	1.2	Male	CD	747.0	1.7E-03
BC066	1.2	Male	KD	268.0	4.3E-03
BC072	1.7	Male	KD	545.0	3.2E-03
BC098	0.5	Female	CD	97.3	4.7E-03
BC104	0.1	Female	CD	213.3	4.7E-04
BC110	2.3	Female	CD	453.0	5.0E-03
BC100	1.5	Female	KD	410.3	3.6E-03
BC106	0.1	Female	KD	145.7	3.9E-04
BC112	1.6	Female	KD	567.0	2.8E-03

**Descriptive statistics**	CD Male	KD Male	CD Female	KD Female
Number of values	3	2	3	3
Minimum	1.7E-03	3.2E-03	5.0E-04	4.0E-04
Maximum	2.2E-03	4.3E-03	5.0E-03	3.6E-03
Range	5.0E-04	1.1E-03	4.5E-03	3.2E-03
Mean	1.9E-03	3.8E-03	3.4E-03	2.3E-03
Std. Deviation	2.6E-04	7.8E-04	2.5E-03	1.7E-03
Std. Error of Mean	1.5E-04	5.5E-04	1.5E-03	9.6E-04

**Kruskal-Wallis test**	Neuron
P value	0.47
Exact or approximate P value?	Exact
P value summary	ns
Do the medians vary signif. (P < 0.05)?	No
Number of groups	4
Kruskal-Wallis statistic	2.848

**Dunn's multiple comparisons test**	**Mean rank diff.**	**Significant?**	**Summary**	**Adjusted P Value**
CDM vs. KDM	-4.0	No	ns	>0.99
CDM vs. CDF	-3.7	No	ns	>0.99
CDM vs. KDF	-1.0	No	ns	>0.99
KDM vs. CDF	0.3	No	ns	>0.99
KDM vs. KDF	3.0	No	ns	>0.99
CDF vs. KDF	2.7	No	ns	>0.99

**Test details**	**Mean rank 1**	**Mean rank 2**	**Mean rank diff.**	**n1**	**n2**	**Z**
CDM vs. KDM	4.0	8.0	-4.0	3	2	1.3
CDM vs. CDF	4.0	7.7	-3.7	3	3	1.4
CDM vs. KDF	4.0	5.0	-1.0	3	3	0.4
KDM vs. CDF	8.0	7.7	0.3	2	3	0.1
KDM vs. KDF	8.0	5.0	3.0	2	3	1.0
CDF vs. KDF	7.7	5.0	2.7	3	3	1.0

Supplementary Table 35: PCR primers

Gene	Strand	Primer sequence	Cell-type
ALDH1L1	forward	GCAGGTACTTCTGGGTTGCT	Astrocyte
NM_027406.2	reverse	GGAAGGCACCCAAGGTCAAA	
GFAP	forward	AGAAAGGTTGAATCGCTGGA	Astrocyte
NM_010277.3	reverse	CGGCGATAGTCGTTAGCTTC	
STMN2	forward	TGCGTGCACATCCCTACAAT	Neuron
NM_025285.2	reverse	TGCTTCACCTCCATGTCGTC	
SYN1	forward	TCTGACCAATGCCTTCAACCT	Neuron
NM_013680.4	reverse	GAGAAGAGGCTGGCGAAAGA	
