# Supplementary material for: Sex-specific systemic and brain metabolic responses to a standardized ketogenic diet in mice
Source: Lab Anim (NY). 2026 May 12;55(6):230–40. doi: 10.1038/s41684-026-01732-7 (PMC13233310; doi:10.1038/s41684-026-01732-7)
Supplement: Supplementary file 2 — Reporting Summary [file 41684_2026_1732_MOESM2_ESM.pdf]

nature portfolio

Corresponding author(s): DBPR, LABAN-A02158

Last updated by author(s): Oct 4, 2024

Reporting Summary

Nature Portfolio wishes to improve the reproducibility of the work that we publish. This form provides structure for consistency and transparency in reporting. For further information on Nature Portfolio policies, see our Editorial Policies and the Editorial Policy Checklist.

Statistics

For all statistical analyses, confirm that the following items are present in the figure legend, table legend, main text, or Methods section.

n/a	Confirmed	
☐	☒	The exact sample size (*n*) for each experimental group/condition, given as a discrete number and unit of measurement
☐	☒	A statement on whether measurements were taken from distinct samples or whether the same sample was measured repeatedly
☐	☒	The statistical test(s) used AND whether they are one- or two-sided<br>*Only common tests should be described solely by name; describe more complex techniques in the Methods section.*
☐	☒	A description of all covariates tested
☐	☒	A description of any assumptions or corrections, such as tests of normality and adjustment for multiple comparisons
☐	☒	A full description of the statistical parameters including central tendency (e.g. means) or other basic estimates (e.g. regression coefficient) AND variation (e.g. standard deviation) or associated estimates of uncertainty (e.g. confidence intervals)
☐	☒	For null hypothesis testing, the test statistic (e.g. *F*, *t*, *r*) with confidence intervals, effect sizes, degrees of freedom and *P* value noted<br>*Give P values as exact values whenever suitable.*
☒	☐	For Bayesian analysis, information on the choice of priors and Markov chain Monte Carlo settings
☐	☒	For hierarchical and complex designs, identification of the appropriate level for tests and full reporting of outcomes
☐	☒	Estimates of effect sizes (e.g. Cohen's *d*, Pearson's *r*), indicating how they were calculated

Our web collection on statistics for biologists contains articles on many of the points above.

Software and code

Policy information about availability of computer code

Data collection	LASX (Leica Application Suite X),
Data analysis	Fiji-ImageJ, CytExpert Software (Beckman Coulter), Excel, GraphPad Prism, G*Power (Heinrich Heine Universität), R

For manuscripts utilizing custom algorithms or software that are central to the research but not yet described in published literature, software must be made available to editors and reviewers. We strongly encourage code deposition in a community repository (e.g. GitHub). See the Nature Portfolio guidelines for submitting code & software for further information.

Data

Policy information about availability of data

All manuscripts must include a data availability statement. This statement should provide the following information, where applicable:

- Accession codes, unique identifiers, or web links for publicly available datasets
- A description of any restrictions on data availability
- For clinical datasets or third party data, please ensure that the statement adheres to our policy

All data supporting the findings of this study are available within the article and its supplementary files.

Human research participants

Policy information about studies involving human research participants and Sex and Gender in Research.

Reporting on sex and gender	*Use the terms sex (biological attribute) and gender (shaped by social and cultural circumstances) carefully in order to avoid confusing both terms. Indicate if findings apply to only one sex or gender; describe whether sex and gender were considered in study design whether sex and/or gender was determined based on self-reporting or assigned and methods used. Provide in the source data disaggregated sex and gender data where this information has been collected, and consent has been obtained for sharing of individual-level data; provide overall numbers in this Reporting Summary. Please state if this information has not been collected. Report sex- and gender-based analyses where performed, justify reasons for lack of sex- and gender-based analysis.*
Population characteristics	*Describe the covariate-relevant population characteristics of the human research participants (e.g. age, genotypic information, past and current diagnosis and treatment categories). If you filled out the behavioural & social sciences study design questions and have nothing to add here, write "See above."*
Recruitment	*Describe how participants were recruited. Outline any potential self-selection bias or other biases that may be present and how these are likely to impact results.*
Ethics oversight	*Identify the organization(s) that approved the study protocol.*

Note that full information on the approval of the study protocol must also be provided in the manuscript.

Field-specific reporting

Please select the one below that is the best fit for your research. If you are not sure, read the appropriate sections before making your selection.

☒ Life sciences ☐ Behavioural & social sciences ☐ Ecological, evolutionary & environmental sciences

For a reference copy of the document with all sections, see nature.com/documents/nr-reporting-summary-flat.pdf

Life sciences study design

All studies must disclose on these points even when the disclosure is negative.

Sample size	The sample size was calculated on the blood ketone level difference between KD and KD ground found in literature. An alpha level of 0.05, a power of 0.8, and a large effect size of 0.8, as defined by Cohen's d, were used. According to G*Power software (Heinrich Heine Universität), 10 mice per group was the estimated number of animals per diet group to successfully achieve adequate statistical power and provide data for this project.
Data exclusions	No animals were excluded from the study.
Replication	To verify the reproducibility of the experimental findings, G*Power software estimated that 10 mice per group was the number of animals per diet group to successfully achieve adequate statistical power and provide reliable data for this project.
Randomization	The mice of equivalent weight were housed in groups of 3-4 per cage
Blinding	Cages were assigned identification numbers so that the experimenter could not deduce the diet of the mice. For the chemistry analysis, the experimenter was blind to the study design.

Reporting for specific materials, systems and methods

We require information from authors about some types of materials, experimental systems and methods used in many studies. Here, indicate whether each material, system or method listed is relevant to your study. If you are not sure if a list item applies to your research, read the appropriate section before selecting a response.

Materials & experimental systems

n/a	Involved in the study
☐	☒ Antibodies
☒	☐ Eukaryotic cell lines
☒	☐ Palaeontology and archaeology
☐	☒ Animals and other organisms
☒	☐ Clinical data
☒	☐ Dual use research of concern

Methods

n/a	Involved in the study
☒	☐ ChIP-seq
☐	☒ Flow cytometry
☒	☐ MRI-based neuroimaging

Antibodies

Antibodies used

mouse anti-GFAP (1:100; MAB360, Merck), and rabbit anti-Aldehyde ALDH1L1 (1:500; ab87117, Abcam), anti-mouse Alexa Fluor 488 (1:1000; A10680, Invitrogen), and donkey anti-rabbit Alexa Fluor 647 (1:1000; ab150075, Abcam), mouse anti-ACSA2 conjugated to PE (1:50, 130-116-244, Miltenyi).

Validation

MAB360: According to the manufacturer website: routinely evaluated by Western Blot on Mouse brain lysates, analysis: 1:1000 dilution of this lot detected GFAP on 10 µg of Mouse brain lysates.

ab87117: According to the manufacturer: The exact immunogen used to generate this antibody is proprietary information, cited in 74 publications, reacts with mouse and rats, Abcam antibodies are extensively validated in a wide range of species and applications.

A10680: According to the manufacturer: 274 references, These goat anti-mouse IgM/IgG (H+L) whole secondary antibodies have been affinity-purified and show minimum cross-reactivity. ross-adsorption or pre-adsorption is a purification step to increase specificity of the antibody resulting in higher sensitivity and less background staining. The secondary antibody solution is passed through a column matrix containing immobilized serum proteins from potentially cross-reactive species. Only the nonspecific-binding secondary antibodies are captured in the column, and the highly specific secondaries flow through. The benefits of this extra step are apparent in multiplexing/multicolor-staining experiments (e.g., flow cytometry) where there is potential cross-reactivity with other primary antibodies or in tissue/cell fluorescent staining experiments where there may be the presence of endogenous immunoglobulins. Alexa Fluor dyes are among the most trusted fluorescent dyes available today. Invitrogen™ Alexa Fluor 488 dye is a bright, green-fluorescent dye with excitation ideally suited to the 488 nm laser line.

ab150075: According to the manufacturer: 5 reviews, This antibody was isolated by affinity chromatography using antigen coupled to agarose beads. Affinity purification Immunogen.

130-116-244: According to the manufacturer: Clone REA969 recognizes the mouse ACSA-2 antigen (ACSA-2: astrocyte cell surface antigen-2), which has been developed for the detection of astrocytes from cell suspensions of mouse neural tissue based on the expression of the ACSA-2 protein. The ACSA-2 antigen is specifically expressed on GLAST (ACSA-1) positive astrocytes and is therefore a specific marker of astrocytes in the developing and neonatal mouse central nervous system (CNS). The percentage of ACSA-2 positive astrocytes differs according to the mouse age and the brain region. Additional information: Clone REA969 displays negligible binding to Fc receptors. 2 references.

Animals and other research organisms

Policy information about studies involving animals; ARRIVE guidelines recommended for reporting animal research, and Sex and Gender in Research

Laboratory animals

A total of 60 healthy C57BL/6J mice, males (N = 40) and females (N =20), of 8-week-old, were used to investigate the impact of the KD (Strain code: 632, Charles River Laboratories).

Wild animals

Provide details on animals observed in or captured in the field; report species and age where possible. Describe how animals were caught and transported and what happened to captive animals after the study (if killed, explain why and describe method; if released, say where and when) OR state that the study did not involve wild animals.

Reporting on sex

males (N = 40) and females (N =20),

Field-collected samples

The mice of equivalent weight were housed in groups of 3-4 per cage and maintained on a 12-hour light-dark cycle with ad libitum access to food and water. No animals were excluded from the study.
Mice were rapidly anesthetized with isoflurane (5% in oxygen) in a gas chamber and decapitated.

Ethics oversight

Registered in a license (national permit number 33876), all procedures were conducted following the Swiss Welfare Act, and the Swiss National Institutional and Veterinary Office guidelines in the Canton of Vaud on Animal Experimentation.

Note that full information on the approval of the study protocol must also be provided in the manuscript.

Flow Cytometry

Plots

Confirm that:

☒ The axis labels state the marker and fluorochrome used (e.g. CD4-FITC).

☒ The axis scales are clearly visible. Include numbers along axes only for bottom left plot of group (a 'group' is an analysis of identical markers).

☒ All plots are contour plots with outliers or pseudocolor plots.

☒ A numerical value for number of cells or percentage (with statistics) is provided.

Methodology

Sample preparation	Astrocytic fraction purity after MACS was assessed using mouse anti-ACSA2 conjugated to PE (1:50, 130-116-244, Miltenyi). After washes in PBS, the extract was centrifuged at 300 g for 10 min, resuspended in PBS.
Instrument	AutoMACS Pro Cell separator from Miltenyi Biotec.
Software	CytExpert Software from Beckman Coulter (V 2.5.0.77)
Cell population abundance	Cell viability was assessed with propidium iodide and analyzed in the phycoerythrin (PE) channel (585/42) using a 4-laser Cytoflex S (Beckman Coulter). The cell survival rate was estimated at 85%, on average (data not shown).
Gating strategy	Astrocytes population was defined using the positive ctrl for ACSA-2 PE positive population. Confirmation of the gating was done by analyze of the unstained to define background.<br>FSC & SSC was defined after running neg ctrl to visualize the background. Astrocytes population was confirmed by back gating the positive population (Astrocytes) of ACSA-2 PE positive on FSC & SSC dot plot.

☒ Tick this box to confirm that a figure exemplifying the gating strategy is provided in the Supplementary Information.
